# Transcriptome sequencing of the choroid plexus in schizophrenia

**DOI:** 10.1038/tp.2016.229

**Published:** 2016-11-29

**Authors:** S Kim, Y Hwang, D Lee, M J Webster

**Affiliations:** 1 Stanley Brain Research Laboratory, Stanley Medical Research Institute, Rockville, MD, USA; 2 Department of Bio and Brain Engineering, KAIST, Daejeon, Korea

## Abstract

The choroid plexus (CP) has a key role in maintaining brain homeostasis by producing cerebrospinal fluid (CSF), by mediating transport of nutrients and removing metabolic products from the central nervous system and by responding to peripheral inflammatory signals. Although abnormal markers of immune response and inflammation are apparent in individuals with schizophrenia, the CP of these individuals has not been characterized. We therefore sequenced mRNA from the CP from two independent collections of individuals with schizophrenia and unaffected controls. Genes related to immune function and inflammation were upregulated in both collections. In addition, a co-expression module related to immune/inflammation response that was generated by combining mRNA-Seq data from both collections was significantly associated with disease status. The immune/inflammation-related co-expression module was positively correlated with levels of C-reactive protein (CRP), cortisol and several immune modulator proteins in the serum of the same individuals and was also positively correlated with CRP, cortisol and pro-inflammatory cytokines in the frontal cortex of the same individuals. In addition, we found a substantial number of nodes (genes) that were common to our schizophrenia-associated immune/inflammation module from the pooled data and a module we generated from lippopolysaccharides-treated mouse model data. These results suggest that the CP of individuals with schizophrenia are responding to signals from the periphery by upregulating immune/inflammation-related genes to protect the brain and maintain the homeostasis but nevertheless fails to completely prevent immune/inflammation related changes in the brain.

## Introduction

Schizophrenia is a common psychiatric disorder that is believed to be caused by a complex interaction between multiple genetic and environmental factors. Although the pathophysiology of the disorder is not well understood, there are now numerous studies that show abnormalities in markers of the immune and inflammatory systems in the blood^1, 2, 3, 4, 5, 6^ and cerebrospinal fluid (CSF)^7, 8, 9^ of individuals with schizophrenia. There are also a number of studies showing abnormalities in markers of immune function and inflammation within the brains of schizophrenia cases,^10, 11, 12, 13, 14, 15, 16^ however, there is scant evidence for lymphocytic infiltration into the brain,^17^ and there is contradictory and inconclusive evidence for activation of the microglia or resident immune cells of the brain in schizophrenia cases.^14, 17, 18^ In contrast, our recent transcriptome sequencing data from the hippocampus^15^ indicated that the abnormally expressed molecules related to immune and inflammation pathways were more likely to be expressed in endothelial cells of blood vessels, in blood monocytes within the blood vessels and in the perivascular astrocytes in the schizophrenia cases, than in lymphocytes or microglia. Thus, the blood–brain-barrier (BBB) may be functioning abnormally in the schizophrenia cases or cells proximal to the BBB may be responding to aberrant factors in the blood of patients with schizophrenia that then causes abnormal function in neurons. Previous evidence for abnormalities in the BBB was provided by a laser capture microdissection study of cerebral vascular endothelial cells that found abnormal expression of genes relating to the inflammatory process in schizophrenia.^19^ An electron microscope study also found ultrastructural abnormalities in endothelial cells and astrocytic end-feet in the frontal cortex of patients with schizophrenia.^20^

In addition to the endothelial BBB, there is also the epithelial blood–cerebrospinal fluid-barrier (BCSFB), which together, protects the brain from invading microorganisms and maintain the homeostatic environment of the central nervous system (CNS). Whereas, the BBB is defined by the cerebral vasculature, the choroid plexus (CP) defines the BCSFB. The CP is a very specialized, highly vascular ependymal structure in the ventricles of the brain where CSF is produced. The CP is structured to restrict cellular and molecular traffic between the blood and CSF,^21^ and acts as a filtration system, removing metabolic waste, foreign substances and excess neurotransmitters from the CSF. An inflammatory response is induced in the CP after injection of a peripheral inflammatory stimulus such as the bacterial endotoxin lipopolysaccharide (LPS)^22^ or a parasite.^23^ This response may be expected given the tropism of various bacteria, parasites and viruses for the CP.^23, 24, 25^ Several studies have also shown changes in CP gene regulation after chronic stress^26^ and in response to treatment^27^ indicating that the CP may be an important site to monitor CNS response to environmental stimuli. Given the unique structural and functional position of the CP at the interface between two circulating fluid compartments, its ability to respond to environmental stimuli and its ability to influence the brain via immune and humoral signals, we sequenced the CP transcriptome of 29 subjects with schizophrenia and 26 unaffected controls from the Stanley Array Collection to further understand the molecular pathways that may be impacting the CNS of individuals with schizophrenia. As a replication study, we sequenced the CP transcriptome of 19 subjects with schizophrenia and 19 unaffected controls from the New Stanley Collection.

## Materials and methods

### CP samples

RNA for the sequencing study was extracted from post-mortem CP at the level of the posterior lateral ventricle from 30 subjects with schizophrenia and 26 unaffected controls that belong to the Stanley Array Collection (AC). It should be noted that one schizophrenia case that died from septicemia and acute pancreatitis was excluded from the downstream analysis because this factor could have directly contributed to elevated immune markers at time of death. The two groups were previously matched for age, gender, race, post-mortem interval (PMI), mRNA quality, brain pH and hemisphere. The subjects in this study have been included in numerous neuropathology studies, gene expression microarray studies and single-nucleotide polymorphism association studies. The data from these studies is available at http://sncid.stanleyresearch.org (SNCID). A replication sequencing study was conducted on 20 subjects with schizophrenia and 19 unaffected controls that belong to the New Stanley Collection. One schizophrenia case was excluded from downstream analysis because the patient had an autoimmune condition resulting in alopecia. Descriptive variables for samples from both tissue collections are listed in Supplementary Table 1. To compare continuous variables such as age, brain pH, PMI and RNA integrity number (RIN) between schizophrenia and controls we first performed a Shapiro-Wilk test for normality and found brain pH and PMI of the AC samples as well as PMI and RIN of the New Stanley Collection samples were not normally distributed (all *P*<0.05). Therefore, the nonparametric Kruskal–Wallis test was performed for group differences of continuous variables and Fisher's exact test performed for nominal variable.

### Library preparation and mRNA sequencing

Extracted total RNA was analyzed using an Agilent 2100 Bioanalyzer (Agilent, Palo Alto, CA, USA). Total RNA (1 μg) was subjected to two rounds of hybridization to oligo(dT) beads (Dynal, Waltham, MA, USA). The resulting mRNA was then used as a template for cDNA synthesis. The mRNA was randomly fragmented to between 200 and 700 bp by focused acoustic shearing (Covaris, Woburn, MA, USA) and converted to first strand cDNA using Superscript III (Invitrogen, Carlsbad, CA, USA), followed by second-strand cDNA synthesis using *Escherichia coli* DNA pol I (Invitrogen). The double stranded cDNA library was further processed by Illumina Genomic DNA Sample Prep kit (Illumina, San Diego, CA, USA). It involved end repair using T4 DNA polymerase, Klenow DNA polymerase and T4 polynucleotide kinase followed by a single <A> base addition using Klenow 3′ to 5′ exo- polymerase, and was ligated with Illumina's adaptor oligo mix using T4 DNA ligase. Adaptor-ligated library was size selected by electrophoretic separation on a 2% agarose gel and excising of the library smear at 500 bp. The library was PCR amplified for 18 cycles using Phusion polymerase and purified by Qiaquick PCR Purification Kit (Qiagen, Valencia, CA, USA) and quantified by Quant-iT picogreen dsDNA Assay Kit (Invitrogen) following the manufacturer's protocol. We then prepared HiSeq 2000 paired-end flow cell on the supplied Illumina cBot and generated 101- bp paired-end sequence reads on the Illumina HiSeq 2000 platform following the manufacturer's protocol. Images taken during the sequencing reactions were processed in five stages by Illumina's software: Image analysis is performed by the instrument control software (Hiseq Control Software, Illumina), base calling is performed by the instrument control software's Real Time Analysis (RTA).

### Reads mapping and differentially expressed genes

All reads were mapped to UCSC *H. sapiens* reference genome (build hg19) using TopHat v2.0.9 with UCSC refFlat gene model annotation file on the –*G* parameter.^28^ We used the expected mean inner distance between mate paired-ends as –*r* parameter. TopHat calls Bowtie v2.1.0 to perform the alignment with no more than two mismatches. We used the pre-built index files of UCSC *H. sapiens* hg19, which are downloaded from the TopHat homepage (http://tophat.cbcb.umd.edu/index.html). The quantification of gene expression was accomplished by HTseq v0.5.3p9 ^(ref. 29)^ and edgeR package.^30^ All mapped read counts of the genes were counted by htseq-count (subprogram of HTseq)^29^ with UCSC refFlat gene model annotation file, no strand specific option, and intersection-nonempty option. The differentially expressed genes between schizophrenia and controls were identified using edgeR^30^ and the false discovery rates <0.05 were considered significant.

### Mouse CP gene expression data

Microarray data from the CP of a previous mouse study^31^ was downloaded from the GEO database. The datasets (GSE23714) consist of normalized gene expression data from Illumina Whole-genome Mouseref-8 expression beadchips (Illumina). The data was from the CP of mice injected with LPS and killed 1, 3 and 72 h after the treatment. Control mice were injected with saline solution (*n*=10).^31^ The top 5000 probes from each treatment condition were filtered based on the coefficient of variation to select genes showing a high dynamic range of gene expression^32, 33^ and were then used as input for weighted correlation network analysis (WGCNA)^34^ to generate a network.

### Gene co-expression network analysis

We constructed co-expression networks using RNA-Seq data for all genes expressed in the CP and pooled the data from both schizophrenia and control groups. Surrogate variable analysis (SVA)^35^ was used to identify the potential confounding effects in the RNA-Seq data and then the confounding effects were adjusted using the resulting surrogate variables from the SVA. The standardized residuals from the linear regression were used to generate gene co-expression networks using WGCNA.^34^ To construct a weighted co-expression network we selected the power for which scale-free topology fitting index (*R*^2^) is ⩾0.9.^36^ Correlation analyses were performed between co-expression modules and traits such as diagnosis and demographic or clinical variables to identify modules that were associated with disorder and/or confounding factors. Multivariate permutation testing of correlations was performed to adjust for multiple testing using MPTCorr.r package^37^ (http://www.psych.umn.edu/faculty/waller/downloads/mpt/mptcorr.r). The statistical package was developed to perform multivariate permutation tests of the correlations between a single criterion variable and multiple predictor variables.^37^ We used a trait as a criterion variable and the eigengene values in all modules as multiple predictor variables. Adjusted *P*-values <0.05 were considered significant. The network connections were visualized using VisANT.^38^

### Network comparisons between schizophrenia and controls

Separate co-expression networks were generated for each group independently by using only schizophrenia data or only control data in the network. We could then compare the co-expression modules of the schizophrenia group to that of the unaffected control group. Module preservation was examined using a permutation test procedure implemented in the WGCNA.^39^ Summary preservation *Z* score (*Z*_summary_) was used to determine whether a co-expression module was preserved in a test network as compare with a reference network. Previous threshold guidelines were followed: if *Z*_summary_ <2, then there is no evidence that the module is preserved. If 2<*Z*_summary_<10, then there is weak to moderate evidence that the module is preserved. If *Z*_summary_>10, then there is strong evidence that the module is preserved.^39^

### Consensus module analysis

Co-expression modules from two independent datasets, the AC and the New Stanley Collection, were compared to identify the consensus modules. *P*-values of significance for each of the pairwise overlaps were obtained by permutation tests in *R*. As we performed 1 million permutations, the lowest possible *P*-value we can obtain from our analysis is 1e−05. The immune-related module from the combined data of the AC and the New Stanley Collection and the immune-related module from the mouse CP gene expression data were compared by the permutation test. The consensus network connections were identified and visualized using Cytoscape.^40^

### Correlation analysis between the immune/inflammation related co-expression module and markers in the serum and frontal cortex of the same cases

Correlation analyses were performed between the immune/inflammation related co-expression module and protein markers related to the same biological processes. The protein levels were measured in serum and frontal cortex of the same individuals from the AC using the DiscoveryMAP multiplexed immunoassay panel at Myriad-RBM (Austin, TX, USA).^41^ The raw data is available in the SNCID. We downloaded the raw data and used it as traits for the correlation analysis using WGCNA. *P*-values <0.05 were considered significant.

### Functional annotation

DAVID (http://david.abcc.ncifcrf.gov/home.jsp) was used to identify the biological processes that were significantly over-represented by differentially expressed genes between schizophrenia and normal controls as well as genes included in the co-expression modules.^42^ Gorilla (http://cbl-gorilla.cs.technion.ac.il/) was used to annotate significantly enriched biological processes in the top 5000 probes that were selected from data of mice 3 h post LPS treatment and controls,^43^ because DAVID does not allow more than 3000 genes or probes as input. *P*-values <0.05 were considered significant.

### Code availability

Codes used for permutation tests are available from the corresponding author.

## Results

### Differential gene expression in CP of individuals with schizophrenia

We analyzed RNA-Seq data from CP of 29 subjects with schizophrenia and 26 unaffected controls from the array collection. There were no significant differences of age, sex, PMI, RIN and brain pH between schizophrenia and controls (Supplementary Table 1). For each sample, we generated an average of 51 million 101-bp reads (Supplementary Table 2). Sequence reads were mapped to the human reference genome (hg19) and for each sample the average number of mapped reads was 36.6 million. We then quantified the aligned read counts and performed statistical analysis. Twenty-seven genes were significantly differentially expressed between schizophrenia and unaffected controls in the CP at false discovery rate<0.05 (Supplementary Table 3). The expression of 23 genes was upregulated, and 4 genes were down-regulated.

Although 25 biological processes were significantly enriched in the upregulated genes in the CP of schizophrenia patients (Supplementary Table 4), there was no significantly enriched biological processes in the down-regulated genes. Defense, immune and inflammatory responses and amino acid transport were the main biological processes that were significantly enriched in the differentially expressed genes (Supplementary Table 4). The genes involved in the defense/inflammatory/immune responses were predominantly upregulated in the CP of schizophrenic patients as compared with the unaffected controls. Although the functional annotation tool listed nine genes, *IL18R1*, *SLC11A1*, *SERPINA3*, *PLA2G*2A, *PTX3*, *THBS1*, *CD163*, *TNFSF14* and *CLEC4D*, as defense/inflammatory/immune response-related, our independent interrogation of the other differentially expressed genes revealed that two additional genes are also involved in immune response, for example, *LRG1* (leucine-rich alpha-2-glycoprotein 1) is involved in granulocyte differentiation^44^ and *STEAP4* (STEAP family member 4) is a metalloreductase involved in the inflammatory response.^45, 46^

### Gene co-expression network analysis

**Figure 1:**
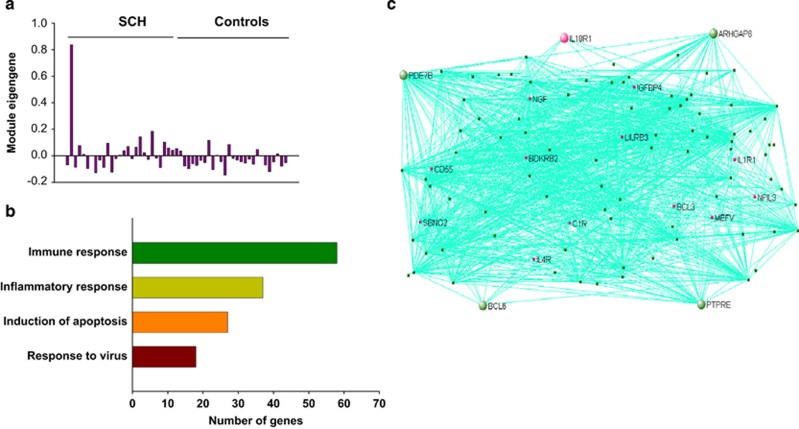
Co-expression module related to immune/inflammation response in choroid plexus. The eigengene values across samples in the S_M16 module (**a**); major biological processes (gene ontology) significantly enriched in the genes in the co-expression module (**b**) and visualization of the module (**c**). The network connections of top 100 genes with topological overlap above the threshold of 0.2 were visualized using VisANT.^38^ The hub genes are larger circles in the network. Genes related to immune/ inflammation responses are red.

An unsupervised co-expression network analyses was performed by combining the RNA-Seq data, which was adjusted for covariates using SVA from all schizophrenia cases and controls to identify the co-expression modules associated with schizophrenia. Of the 18 co-expression modules that were generated with this combined data, none of the modules were significantly associated with schizophrenia using the multivariate permutation testing of correlations (Supplementary Table 5). Among all modules, the S_M16 module was the most significantly associated with schizophrenia, however, the *P*-value did not reach statistical significant level (*r*=0.3, *P*_adj_=0.26). The S_M16 was a large module containing 629 genes. The eigengene values in this module were over expressed in schizophrenia cases as compared with controls (Figure 1a), and immune and inflammation response, apoptosis, response to virus and hypoxia were significantly enriched in this module (Figure 1b; Supplementary Table 6). *IL18R1* (interleukin 18 receptor 1), *PTPRE* (protein tyrosine phosphatase, receptor type E), *ARHGAP6* (Rho GTPase activating protein 6), *PDE7B* (phosphodiesterase 7B) and *BCL6* (B-cell CLL/lymphoma 6) are the top 5 hub genes in this module (Figure 1c).

### Comparing the separate co-expression networks of schizophrenia and unaffected controls

To further explore fundamental differences in gene–gene interactions in the CP of individuals with schizophrenia as compared with controls, we constructed co-expression networks for schizophrenia and controls separately. We then compared the co-expression modules for schizophrenia to those for controls, or vice versa and found that almost all the modules were conserved between the schizophrenia cases and controls indicating that there is no difference in the gene–gene interactions between cases and controls. However, two modules were only weakly conserved indicating that there are some possible differences in the gene–gene interactions between the cases and controls (Supplementary Table 7). One module, C_only1_M8 was only weakly conserved in schizophrenia as compared with controls and therefore more likely to be specific to controls, whereas the other module, SCH_only_M7 was only weakly conserved in controls as compared with schizophrenia (2<*Z*_summary_<10, Supplementary Table 7) and therefore more likely to be schizophrenia specific. The control-specific module, C_only1_M8, was negatively correlated with brain pH (Supplementary Table 8). Regulation of signal transduction, cell cycle and transcription were the main biological processes associated with this module (Supplementary Table 9). The schizophrenia specific module, SCH_only_M7, was not correlated with any covariates we tested (Supplementary Table 10) and contained 76 genes with *BAG3*, *DNAJB1*,*DNAJB4*, *HSPAH* and *HSPA6* the top 5 hub genes in this module (Supplementary Figure 1a). Response to organic substances, regulation of apoptosis and cell proliferation were the process significantly enriched in the co-expressed genes (Supplementary Figure 1b; Supplementary Table 11).

### Association between the immune/inflammation related co-expression module and markers in the serum and frontal cortex

A correlation analysis was performed to explore the association between the S_M16 module and the protein markers measured in the serum and frontal cortex of the same individuals. Thirty and twenty-three markers related to immune/inflammation response were significantly correlated with the S _M16 module in the serum and the frontal cortex, respectively (Table 1). Five markers, including CRP, cortisol, TIMP metallopeptidase inhibitor 1 (TIMP1), matrix metallopeptidase 9 (MMP9) and resistin (RETN) were positively correlated with the module in both the serum and the frontal cortex. Immune modulators such as IL1RN and IL25 were positively correlated with the module in the serum, whereas pro-inflammatory cytokines such as IL1B and IL6 were positively correlated with the module in the frontal cortex.

### Validation of RNA-seq results using an independent tissue collection

We used an independent tissue collection to replicate the differentially expressed genes and the co-expression modules associated with schizophrenia in the CP. There were no significant differences of age, brain pH, RIN and PMI between schizophrenia and controls in this cohort (Supplementary Table 1). However, sex was significantly different between schizophrenia and controls in this cohort (*P*=0.001; Supplementary Table 1). We generated an average of 99.9 million 101-bp reads and for each sample the average number of mapped reads was 90.9 million (Supplementary Table 12). Although there were 33 genes significantly differentially expressed between schizophrenia and unaffected controls in the CP at false discovery rate<0.05 (Supplementary Table 13), there was no significantly enriched biological processes in the genes. However, there was a significant overlap between the differentially expressed genes from the AC and the New Stanley Collection data (*P*<1e−05). Three genes, *SERPINA3*, *ADAMTS17* and *CISH* were differentially expressed in both collections.

**Figure 2:**
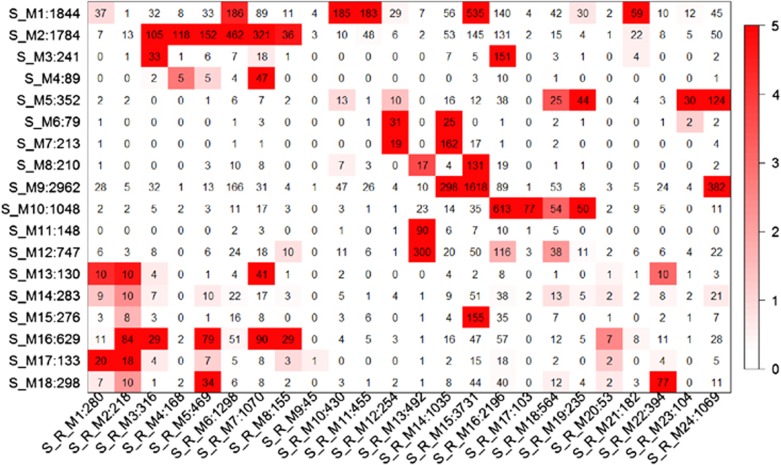
Pairwise comparisons of the modules between the AC data and the New Stanley data. The color code of the heatmap encodes −log (*P*-value). The *P*-values were calculated by permutation test for the overlap of the two modules. The numbers in the heatmap indicate gene counts in the intersection of two modules. The label to the left of the *y-*axis of a heat map indicates modules of the AC data and gene counts in the modules. The label to the bottom of the *x*-axis of the heatmap indicates modules of the New Stanley Collection data and gene counts in the modules. AC, Array Collection.

Twenty-four co-expression modules were built using the combined data from schizophrenia and controls and although one (S_R_M9) was significantly associated with schizophrenia, it was also highly significantly correlated with sex (Supplementary Table 14), and therefore excluded from downstream analysis. When comparing the nodes (genes) in the modules from the New Collection to those from the AC data, we found significant overlap of genes common to both datasets (Figure 2). Although most modules from the New Stanley Collection data have consensus with modules from the AC network (Figure 2), two modules (S_R_M9 and S_R_M20) did not have any consensus with AC modules. The immune-related module, S_M16 from the AC data had significant overlap of common nodes (genes) with five co-expression modules from the New Stanley Collection, S_R_M2, S_R_M3, S_R_M2, S_R_M5, S_R_M7 and S_R_M8. Moreover, S_M16 has the most significant overlap with the S_R_M2, where 84 nodes (38%) were also included in the S_M16 module (*P*<1e−05) (Figure 2). The module S_R_M2 contained 218 genes and cytokine-mediated signaling pathway, defense/ inflammatory responses and apoptosis were significantly enriched in this module (Supplementary Table 15).

Next, we built co-expression networks for schizophrenia and controls separately and then compared them to each other in an attempt to replicate the results from the AC samples. One module, C_R_only_M10, was weakly conserved in schizophrenia as compared to controls and therefore likely to be control specific. (Supplementary Table 16). The module was also significantly correlated with PMI (Supplementary Table 17) and only contained 30 genes that were enriched for gaseous exchange, regulation of cell migration and cell proliferation (Supplementary Table 18).

Of the 23 co-expression modules generated from the schizophrenia only data, two were not preserved at all (S_R_only_M12 and M18) and 3 (S_R_only_M1, M7 and M20) were only weakly preserved in controls and therefore all are likely to be specific to schizophrenia (Supplementary Table 16). The five modules were not significantly correlated with any confounding variables tested (Supplementary Table 19). The S_R_only_M1 module contained 106 genes enriched for immune response and response to virus (Supplementary Table 20). The S_R_only_M12 module was not preserved at all in controls and contained 38 genes enriched for negative regulation of cell proliferation and apoptotic mitochondrial change (Supplementary Table 21). The S_R_only_M18 module was also not preserved at all in controls and contained 116 genes enriched for response to nutrients and regulation of retinoic acid receptor signaling pathway (Supplementary Table 22). The remaining two modules, which were only weakly preserved in controls, contained genes significantly enriched for ion homeostasis and neuropeptide signaling pathways (Supplementary Table 23).

### Network analysis of the pooled data from both AC and new stanley collection

The co-expression module from the AC related to immune/inflammation response was successfully replicated in the independent RNA-seq data set. Although both of these modules were also the modules most significantly associated with schizophrenia, the *P*-values did not reach a statistical significant level in multivariate permutation testing of correlations. Because the sample size was relatively small for each analysis, we repeated the analysis by combining the two datasets to increase the sample size and to increase detection power.

**Figure 3:**
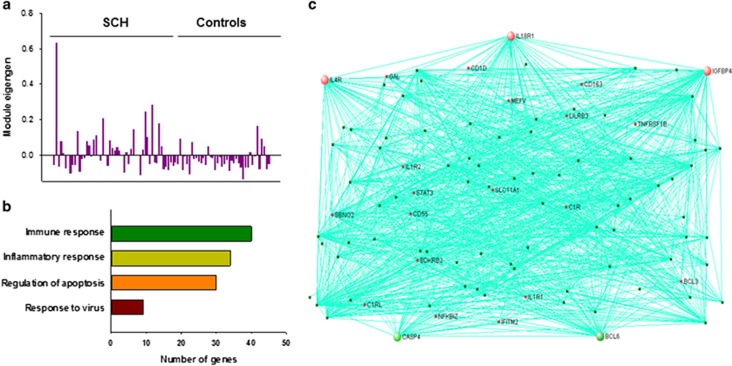
Co-expression module of the combined data associated with schizophrenia and related to immune/inflammation response in choroid plexus. The eigengene values across samples in the S_Co_M16 module (**a**); major biological processes (gene ontology) significantly enriched in the genes in the co-expression module (**b**) and visualization of the module (**c**). The network connections of top 100 genes with topological overlap above the threshold of 0.05 were visualized using VisANT.^38^ The hub genes are larger circles in the network. Genes related to immune/inflammation responses are red.

We generated 23 co-expression modules with the combined data. One module (S_Co_M16) was significantly associated with schizophrenia (*r*=0.311, *P*_adj_=0.03; Supplementary Table 25). The eigengene values in this module were over expressed in schizophrenia cases as compared with controls (Figure 3a), and immune and inflammation response, regulation of apoptosis and response to virus were significantly enriched in this module (Figure 3b; Supplementary Table 26). *IL18R1* (interleukin 18 receptor 1), *IL4R* (interleukin 4 receptor), *IGFBP4* (insulin-like growth factor binding protein 4), *CASP4* (caspase 4) and *BCL6* (B-cell CLL/lymphoma 6) are the top 5 hub genes in this module (Figure 3c).

### Comparison of modules from our human CP study and those from a mouse model

To explore the possible causes of the activated immune/inflammation modules in the CP of the schizophrenia cases, we compared the immune/inflammation module from the combined data to a co-expression module from a well-validated mouse model.^31^ First, we generated co-expression networks from data collected from the CP of LPS-treated mice at 1, 3 or 72 h post treatment and combined the data with that from the control mice. To generate a network using genes showing a high dynamic range of gene expression, the top 5000 probes were filtered based on the coefficient of variation. A total of 15 and 16 co-expression modules were generated with data from mice 1 and 72 h post LPS treatment and controls, respectively. None of the modules was correlated with LPS treatment. In contrast, the co-expression modules generated with data from mice 3 h post LPS treatment and controls did produce a module correlated with LPS treatment. The functional annotation of the top 5000 probes from the 3 h post LPS treatment indicated that immune system process and immune/inflammation response were highly enriched in the list (Supplementary Table 27). Of the 15 co-expression modules built, one module, LPS_3h_M2, was significantly positively associated with LPS treatment (*r*=0.99, *P*_adj_<0.001). Immune and inflammation response, regulation of gene expression and response to LPS were significantly enriched in this module (Supplementary Table 28). These results indicate that the most robust acute response occurred at 3 h post LPS injection, which is consistent with the results from the original study.^31^

**Figure 4:**
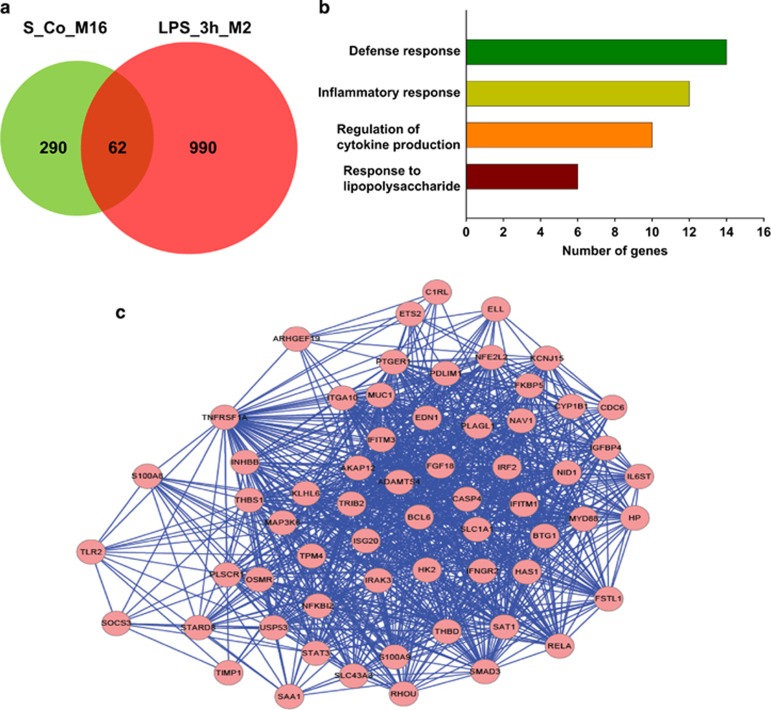
Comparative network analysis of the immune/inflammation related module, S_Co_M16, from the choroid plexus of schizophrenia and controls compared with mice 3 h post lippopolysaccharide treatment. Venn diagram shows the number of common and unique genes between the schizophrenia-associated module S_Co_M16 and the mouse LPS_3h_M2 module (**a**). Major biological processes (gene ontology) significantly enriched in the genes common to both co-expression modules (**b**) and visualization of the network connections of the common genes between the two modules using Cytoscape (**c**).^40^

We then compared the nodes (genes) in this module to those in the schizophrenia-associated module, S_Co_M16 and found a significant number of nodes (62) that were common to both LPS_3h_M2 and the schizophrenia-associated module (*P*<1e−05; Figure 4a). Immune and inflammation response, regulation of cytokine production and response to LPS were significantly enriched in the common genes (Supplementary Table 29; Figures 4b and c).

## Discussion

The CP not only produces CSF, but is also a physical and ‘biochemical' barrier that maintains the internal microenvironment of the CNS.^47^ To maintain this homeostasis the CP is modulated by, and must respond to peripheral inflammatory signals. The hypothesis that neuroimmune mechanisms are having a role in the eitiology and pathophysiology of psychotic disorders is generating renewed interest.^48^ Consequently considerable evidence is accumulating from both the periphery^3^ and brain^10, 11, 12, 13, 14, 15, 49, 50, 51^ to support this hypothesis, and networks constructed from candidate genes for schizophrenia and carcinoma show that immune-related genes are associated with both schizophrenia and tumor suppression.^52^ Few studies have investigated the BBB^19, 20^ or BCSFB/CP^53, 54^ in schizophrenia. Here, we investigate the genes and related biological processes that are dysregulated in the CP of individuals with schizophrenia as compared with unaffected controls. Genome-wide expression profiles of the CP, comparing the schizophrenia group to the matched controls, were generated from two independent tissue collections. We built co-expression networks from the combined gene expression data of both tissue collections and found a module related to immune/inflammation response over expressed in schizophrenia as compared with unaffected controls in both tissue collections. In addition, we found a schizophrenia specific co-expression module related to apoptosis in both tissue collections.

The majority of differentially expressed genes were upregulated in schizophrenia in both collections and many of the genes are involved in immune function and inflammation including the three genes common to both collections, *SERPINA3*, *CISH* and *ADAMTS17*. SERPINA3 (Serpin Peptidase Inhibitor, Clade A (Alpha-1 Antiproteinase, Antitrypsin), Member 3) also known as alpha-1-antichymotrypsin, is a serine protease inhibitor and an acute phase protein that is induced during inflammation. However, SERPINA3 inhibits the activity of proteases, such as cathepsin G thereby limiting inflammation, inhibiting apoptosis and protecting tissues.^55^ Endothelial and epithelial cells, macrophages and astrocytes express SERPINA3. Numerous studies of prefrontal cortex have consistently found *SERPINA3* mRNA expression to be upregulated in schizophrenia^10, 11, 14, 16, 56^ and here we also find it upregulated in the CP of two independent collections. Moreover, alpha-1-antichymotrypsin protein levels are elevated in the frontal cortex and serum of these same cases (SNCID) and the immune/inflammation related co-expression module, S_M16 was positively correlated with alpha-1-antichymotrypsin protein levels in the frontal cortex of the same individuals (Table 1). Thus, it appears that the widespread and consistent upregulation of *SERPINA3* mRNA and protein in schizophrenia may be an attempt to maintain the homeostasis of the CNS in response to a peripheral stimulus. Likewise, CISH (Cytokine Inducible SH2-Containing Protein) inhibits IL-2 and IL-3 signaling and through a feedback mechanism has an important role in controlling the magnitude and duration of the cellular response to extracellular stimuli.^57^ Polymorphisms in the CISH gene are associated with susceptibility to various infectious pathogens.^58^ Finally, ADAMTS17 (ADAM metallopeptidase with thrombospondin type 1 motif, 17) is a zinc-dependent protease and although the precise function of ADAMTS17 is not yet known, the general function of such enzymes in normal tissue remodeling and wound healing may be relevant to maintaining the integrity of the CP as it responds to external stimuli. Together, the upregulation of these three genes in both cohorts indicates that the CP may be mounting a protective stance in response to a peripheral stimulus in the schizophrenia patients.

Both tissue collections each produced a co-expression module, S_M16 and S_R_M2 that were related to immune/inflammation response. The *P*-values for significance of associations between the modules and schizophrenia were the lowest among modules, but did not reach a statistical significant level after adjusting for multiple testing. None of the modules generated from either RNA-Seq data set were significantly associated with schizophrenia, indicating that the sample size may not have been adequate for the network analysis. Therefore, we performed a network analysis with the pooled data of the two tissue collections and identified an immune-related module, S_Co_M16 that was significantly associated with schizophrenia. All three modules associated with schizophrenia were also related to immune/ inflammation response and contained four interleukin receptor genes, *IL1R1*, *IL4R*, *IL15RA* and *IL18R1*. The genes IL1R1 and IL18R1 are receptors for IL1 and IL18, which are both pro-inflammatory cytokines that induce and maintain the inflammatory cascade.^59, 60^ Studies of IL1B serum levels have found consistent abnormalities in schizophrenia^3^ and the co-expression module, S_M16 was positively correlated with IL1B protein levels in the frontal cortex of these same individuals (Table 1). Similarly IL18 serum levels are consistently upregulated in patients with schizophrenia^61, 62, 63, 64, 65^ and IL18 protein levels are significantly increased in the frontal cortex of subjects with schizophrenia (SNCID). IL18 serum levels are also associated with hippocampal volume in schizophrenia^65^ and peripheral inflammatory insult in rats will lead to increased levels of IL18 in the hippocampus and behavioral modifications.^66^ Polymorphisms in the *IL18* and IL18 receptor genes have also been associated with schizophrenia.^67, 68^

The cytokine receptor IL4R binds IL4, which has been referred to as the ‘prototypic immunoregulatory cytokine.' ^69^ The cytokine can activate T, B and natural killer cells but not monocytes. A meta-analysis of serum cytokine levels shows that IL4 levels are not significantly altered in the serum of patients with schizophrenia as compared to controls,^3^ however, RNA levels of IL4R are significantly increased in the hippocampus of patients with schizophrenia.^15^ A recent study has found that in response to sublethal ischemia, IL-4 can be produced and secreted by neurons, resulting in the upregulation of IL4R on microglia and consequently propose this as an endogenous defense mechanism in the brain.^70^ The interleukin 15 (IL15) receptor consists of three subunits, IL2R beta, IL2R gamma and IL15R alpha.^71^ *IL15RA* encodes the alpha subunit that contributes to the high-affinity binding of IL15, which has a broad range of biological functions as an inflammatory and immune modulator responding to microbial infections and parasites.^71^ IL-15 is upregulated in the serum of first episode, antipsychotic-naive patients with schizophrenia.^72, 73^

In addition to these genes common to both collections, we also found genes related to response to virus, such as *MX1* (myxovirus (influenza virus) resistance 1, interferon-inducible) and *MX2* (myxovirus (influenza virus) resistance 2) and genes related to response to molecules of bacterial origin such as *IL6R*, *TLR2* and *STAT1* that were significantly enriched in the S_M16 module. Interleukin 6 (IL6) that binds to IL6R is consistently elevated in the serum of patients with schizophrenia^3^ and the co-expression module, S_M16 was positively correlated with IL6 protein levels in the frontal cortex of the same individuals (Table 1). Toll-like receptor 2 (*TLR2)* expression is induced in the CP during the acute phase of the LPS mouse model^31^ and genes related to IL-2, IL-4, IL-6 and IL-10 signaling were also increased in the CP of mice that received LPS once every 2 weeks for 3 months.^74^ We also found a substantial numbers of genes that were common to the co-expression modules of both the LPS mouse model (LPS_3h_M2) and the schizophrenia-associated immune/inflammation response module (S_Co_M16). Recent large scale genome wide association studies have shown the most significant association between schizophrenia and common variants is on the major histocompatibility complex (MHC) region of chromosome 6.^75, 76, 77^ This result suggests that abnormal immune/inflammation response may be involved in the etiology of schizophrenia. HLA class I genes such as *HLA-A*, *HLA-B* and *HLA-C* are located in the MHC region and are included in the schizophrenia specific module, S_R_only_M1, from our replication study. MHC class I molecules bind to fragments of viral proteins and present them to the cell surface for recognition by CD8^+^ T cells.^78^ In addition, the co-expression module, S_M16 was also positively correlated with CRP levels in both the serum and frontal cortex of the same individuals (Table 1). CRP levels can be increased by infection and/or inflammation,^79^ so taken together, these results suggest that a peripheral stimulus, perhaps viral and/or bacterial, may activate the co-expression module related to immune/inflammation responses in the CP of patients with schizophrenia. Furthermore, these results suggest the possible causal relationship between activated immune/inflammation response-related genes and schizophrenia.

Although antibodies to various infectious agents have been identified in the serum of patients with schizophrenia and are often associated with increased risk for the disorder,^80^ robust hallmarks of neuropathological inflammation in the brain of people with schizophrenia have not been reported. This indicates that if infectious agents are involved in schizophrenia it is likely a mild chronic infection rather than an acute infection. In addition, maternal immune activation (MIA) also may contribute significantly to the increased risk of developing schizophrenia in the offspring,^81^ and it is proposed that once the acute inflammation subsides an abnormal ‘immune memory' may continue to be active in the offspring that then interferes with brain development into adulthood.^82^ The abnormal cytokine and CRP levels and the immune/inflammation co-expression module in the CP of people with schizophrenia may all be signatures of this abnormal ‘immune memory'. Although animal models of MIA often show an increase in cytokine levels in serum and brain of the adult offspring,^83, 84^ other similar studies did not show these effects.^85, 86^ However, MIA appears to have a different effect on cytokine levels at different development stages in the offspring (infancy, adolescence and adulthood), and in different brain regions.^83^ The timing of immune activation, the intensity of immune stimulus and the genetic background may also contribute to the differential effects in the offspring. Interestingly, animals that receive immune activation as adults show changes in mRNA expression of immune-related genes in the frontal cortex that reflects changes seen in the frontal cortex of people with schizophrenia.^84^

The co-expression module, S_M16 was also positively correlated with cortisol protein levels in the serum and in the frontal cortex of the same individuals (Table 1), indicating that the module could be activated in response to chronic stress, which is known to modulate the immune/inflammation response.^87^ Cortisol is a steroid hormone released in response to stress and levels are elevated in serum^88, 89^ and in frontal cortex^41^ of individuals with schizophrenia. Although both human and animal studies show that chronic stress increases inflammatory cytokines in the blood and the brain,^87^ it is also apparent that cytokines such as IL1 and IL6 can act as potent activators of the HPA axis.^88, 90, 91^ Thus, although chronic stress may be contributing to the activated immune/inflammation module in the CP of schizophrenia it is likely part of a complex interaction.

A recent genetic study proposed complement component 4 (C4) as a possible candidate gene in the MHC region of chromosome 6, for the pathogenesis of schizophrenia.^92^ C4, located near the strongest peak of association with schizophrenia at the MHC region,^92^ has two isotypes of the gene, *C4A* and *C4B*, in humans. Besides a key role in innate immune response, the complement system is implicated in synaptic pruning.^93^ C4 protein mediates synaptic plasticity in the mouse model and C4A mRNA expression is significantly higher in individuals with schizophrenia as compared with the unaffected controls.^92^ Three complement genes *C1R*, *C1RL* and *C3* were included in the S_M16 module in this study and *C1R*, *C1RL*, *C1S* and *C1QL* were included in the immune module from the hippocampus RNA-seq data from schizophrenia and controls from our previous study.^94^ These results suggest a possible mechanism for mediating the excess synaptic pruning that may occur in the brain of schizophrenia patients by the activated immune/inflammation related modules of co-expressed genes.

Gene co-expression network analysis is now a standard statistical approach for identifying differentially expressed genes at the systems level when comparing disease cases and controls.^34^ This systems approach may identify gene–gene interactions (that is, connectivity between genes) that contribute to the pathophysiology of the disease.^39^ In this study, we not only performed single-gene comparison and gene co-expression analysis but also performed independent co-expression analysis for cases and controls separately to detect gene–gene interactions that differed between disease cases and unaffected controls. We identified 27 differentially expressed genes in our first datasets and 33 in the replication dataset but only three genes overlapped. In contrast, the co-expression network analysis from the replication dataset yielded results that were more consistent with those from the first dataset. The immune-related module, S-R_M2, from the replication dataset included 84 genes (~40%), which were also included in the immune-related module, S_M_16, from the first dataset. This indicates that reproducibility of the co-expression network analyses may be higher than the classical single-gene comparison analysis. Although none of the modules were significantly associated with schizophrenia in the separate analysis, one immune-related module was significantly associated with schizophrenia in the analysis of the combined datasets. Thus, a larger sample size may be necessary to detect modules associated with schizophrenia in the network analysis.

The co-expression network shows that genes relating to immune function and inflammation are co-expressed in both cases and controls, in both tissue collections, however, the overall expression of the network seemed to be upregulated in schizophrenia as compared with controls. When we built independent co-expression modules for schizophrenia and controls separately and then compared the co-expression modules for schizophrenia to those for controls, or vice versa, we found that almost all the modules were conserved between the schizophrenia cases and controls indicating that there is generally no difference in the gene–gene interactions between cases and controls. However, there were several co-expression networks that were only weakly conserved in controls in both collections and therefore likely to be unique to schizophrenia, however, these were also enriched for genes related to immune response as well as apoptosis. Thus, although there is a network of immune/inflammation related genes in the CP that is responding to stimuli from the periphery in both schizophrenia cases and controls, the response is significantly upregulated in schizophrenia. This upregulated network of genes may then upregulate an additional network of genes related to immune defense and apoptosis that is unique to the CP in the schizophrenia cases.

Genome-wide gene expression profiling studies such as microarray and mRNA sequencing (RNA-Seq) have been widely used as an unbiased approach to identify the pathophysiology of schizophrenia.^10, 11, 12, 13, 14, 15, 95, 96^ Although microarray studies have identified many genes associated with the etiology of schizophrenia,^10, 11, 12, 13, 95, 96^ the results need to be validated using independent methods, such as RT-PCR, because of the high levels of noise in the gene expression measurements.^97^ However, advances in massively parallel sequencing methodology such as RNA-Seq can provide more accurate, sensitive and reliable gene expression data than both microarrays and RT-PCR.^98^ Thus, here we performed a replication and validation study by using RNA-seq on a second independent cohort rather than doing RT-PCR on the same cohort.

In conclusion, the CP of individuals with schizophrenia appears to be responding to peripheral stimuli by upregulating genes related to immune function and inflammation. The significant overlap of co-expression networks found in the CP of schizophrenia patients and of the LPS-treated mouse model indicates that there may be a peripheral challenge from the immune system that is affecting the brain in individuals with schizophrenia. These results also support our previous results that showed that peripheral infection and stress may synergistically underlie the pathophysiology of schizophrenia.^15^ It is apparent in animal models that changes in peripheral immunity can affect higher cognitive brain function,^98, 99^ but it remains to be determined exactly how the peripheral immune system can regulate brain function and behavior.

### Note

The data analysis procedures are outlined in Supplementary Figure 2. Gene symbols for each co-expression module are listed in Supplementary Table S30. The top 5000 probes and corresponding gene symbols that were selected from the data from mice 3 h post LPS treatment and controls are listed in Supplementary Table S31. Both RNA-Seq raw data (FASTQ files), read count data and the protein markers measured in serum and frontal cortex of the individuals from the AC are publicly available for free download at http://sncid.stanleyresearch.org (SNCID).

## Figures and Tables

**Table 1:** Correlation between the co-expression module (S_M16) associated with the immune/inflammation response in the choroid plexus and protein markers in serum and frontal cortex of the same individuals

*Serum*			*Frontal cortex*		
*Marker*	*r*	P*-value*	*Marker*	*r*	P*-value*
TGFB3 (transforming growth factor, beta 3)	0.72	6.86E−10	IL1B (interleukin 1, beta)	0.77	8.39E−12
CSF2 (colony stimulating factor 2 (granulocyte-macrophage)	0.68	1.29E−08	IL6 (interleukin 6)	0.5	0.0001
NGF (nerve growth factor (beta polypeptide))	0.65	7.64E−08	***TIMP1 (TIMP metallopeptidase inhibitor 1)***	0.49	0.0001
Proinsulin (intact)	0.64	1.35E−07	***Cortisol***	0.46	0.0004
Proinsulin (total)	0.63	2.24E−07	PAPPA (pregnancy-associated plasma protein A, pappalysin 1)	0.46	0.0005
***Cortisol***	0.62	4.41E−07	IFNG (interferon, gamma)	0.44	0.0008
Plasminogen activator inhibitor type 1	0.54	2.07E−05	SERPINE1 (serpin peptidase inhibitor, clade E (nexin, plasminogen activator inhibitor type 1), member 1)	0.39	0.0032
B2M (beta-2-microglobulin)	0.51	6.48E−05	IGF1 (insulin-like growth factor 1 (somatomedin C))	0.38	0.0037
CXCL13 (chemokine (C-X-C motif) ligand 13)	0.51	6.76E−05	SERPINA3 (serpin peptidase inhibitor, clade A (alpha-1 antiproteinase, antitrypsin), member 3	0.38	0.0041
***RETN (resistin)***	0.5	9.86E−05	PROS1 (Protein S (alpha))	0.38	0.0043
***CRP (C-reactive protein)***	0.4	0.0023	CD40 (CD40 molecule, TNF receptor superfamily member 5)	0.36	0.0077
IL1RN (interleukin 1 receptor antagonist)	0.36	0.0064	MPO (myeloperoxidase)	0.34	0.0109
***TIMP1 (TIMP metallopeptidase inhibitor 1)***	0.32	0.0169	S100A12 (S100 calcium binding protein A12)	0.34	0.0112
TFF3 (trefoil factor 3)	0.31	0.0207	APCS (amyloid P component, serum)	0.34	0.0123
CSF1 (colony stimulating factor 1 (macrophage))	0.31	0.0211	CCL2 (chemokine (C-C motif) ligand 2)	0.31	0.0193
LCN2 (lipocalin 2)	0.31	0.0229	***RETN (resistin)***	0.31	0.0198
IL12B (interleukin 12B)	0.3	0.0253	TNFRSF1B (tumor necrosis factor receptor superfamily, member 1B)	0.3	0.0266
HSPD1 (heat shock 60kDa protein 1 (chaperonin))	0.3	0.0273	CXCL1 (chemokine (C motif) ligand 1)	0.3	0.0274
***MMP9 (matrix metallopeptidase 9)***	0.29	0.0330	***CRP (C-reactive protein)***	0.29	0.0296
CXCL1 (chemokine (C-X-C motif) ligand 1 (melanoma growth stimulating activity, alpha))	0.28	0.0368	***MMP9 (matrix metallopeptidase 9)***	0.29	0.0339
VWF (von Willebrand factor)	0.28	0.0379	SERPINA1(serpin peptidase inhibitor, clade A (alpha-1 antiproteinase, antitrypsin), member 1)	0.28	0.0353
CST3 (cystatin C)	0.28	0.0398	CSF3 (colony stimulating factor 3 (granulocyte))	0.27	0.0496
IL25 (interleukin 25)	0.27	0.0481	VEGFA (vascular endothelial growth factor A)	−0.3	0.0273
F7 (coagulation factor VII (serum prothrombin conversion accelerator))	−0.27	0.0451			
APOB (apolipoprotein B)	−0.28	0.0379			
IL13 (interleukin 13)	−0.3	0.0255			
TTRE (transthyretin)	−0.32	0.0182			
CXCL1 (chemokine (C motif) ligand 1)	−0.35	0.0098			
IL2 (interleukin 2)	−0.64	1.51E−07			
GCG (glucagon)	−0.71	1.85E−09			
