# Supplementary material for: Transcriptome sequencing of the choroid plexus in schizophrenia
Source: Transl Psychiatry. 2016 Nov 29;6(11):e964–. doi: 10.1038/tp.2016.229 (PMC5290353; doi:10.1038/tp.2016.229)
Supplement: Supplementary Tables 1–29 [file tp2016229x1.docx]

**Supplementary Materials**

**Transcriptome Sequencing of the Choroid Plexus**

**in Schizophrenia**

**Supplementary Table 1**. **Demographic and clinical variables for samples from two tissue collections used in this study.**

|  | **AC samples N=55** | | | **NC samples N=38** | | |
| --- | --- | --- | --- | --- | --- | --- |
|  | Schizophrenia (n=29) | Normal Controls (n=26) | P value* | Schizophrenia (n=19) | Normal Controls (n=19) | P value* |
| Age | 41.3±8.6 | 45.6±7.7 | 0.06 | 46.7±9.2 | 51.4±8.7 | 0.13 |
| Sex (M/F) | 21/8 | 20/6 | 0.76 | 10/9 | 19/0 | 0.001 |
| Brain pH | 6.5±0.2 | 6.6±0.3 | 0.20 | 6.5±0.2 | 6.6±0.2 | 0.15 |
| PMI | 33.2±15.5 | 28.4± 13.4 | 0.22 | 45.8±34.4 | 30.1± 18.4 | 0.07 |
| RIN | 8.1±0.43 | 8.0± 0.42 | 0.30 | 6.9±0.57 | 7.0± 0.72 | 0.28 |
| Antipsychotic | 69831.0±80638.0 | 0 | NA | 52394.7±47330.0 | 0 | NA |

Values are mean ± S.D. The nonparametric Kruskal-Wallis test was performed for group differences of continuous variables and Fisher’s exact test performed for nominal variable, sex.

PMI, post-mortem interval; RIN, RNA integrity number; Antipsychotic, normalized as fluphenazine gram equivalent

**Supplementary Table 2. RIN and RNA-Sequencing mapping statistics**

| **Sample** | **Profile** | **RIN** | **Read length (bp)** | **Total Read** | **Mapped Read^1^** |
| --- | --- | --- | --- | --- | --- |
| A3 | Schizophrenia | 7.4 | 101 | 51782750 | 36711715 |
| A7 | Schizophrenia | 7.9 | 101 | 49413471 | 36396018 |
| A9 | Schizophrenia | 8.1 | 101 | 57426795 | 40567067 |
| A10 | Schizophrenia | 8.1 | 101 | 53929192 | 39648635 |
| A11 | Schizophrenia | 7.2 | 101 | 53765299 | 37471275 |
| A12 | Schizophrenia | 8.3 | 101 | 55111788 | 39799711 |
| A17 | Schizophrenia | 8.8 | 101 | 58874349 | 43434160 |
| A22 | Schizophrenia | 8.4 | 101 | 54292194 | 39634189 |
| A26 | Schizophrenia | 8.2 | 101 | 48423475 | 34615427 |
| A31 | Schizophrenia | 7.4 | 101 | 57424759 | 42374645 |
| A37 | Schizophrenia | 8.5 | 101 | 49643721 | 33275712 |
| A39 | Schizophrenia | 8.3 | 101 | 53920089 | 37328264 |
| A40 | Schizophrenia | 8.4 | 101 | 41198725 | 29150679 |
| A44 | Schizophrenia | 8.6 | 101 | 42540847 | 29792708 |
| A45 | Schizophrenia | 8 | 101 | 51846859 | 34561811 |
| A47 | Schizophrenia | 7.9 | 101 | 47585975 | 32761070 |
| A68 | Schizophrenia | 8.3 | 101 | 55700469 | 40773312 |
| A73 | Schizophrenia | 7.6 | 101 | 49917408 | 35360545 |
| A78 | Schizophrenia | 8.3 | 101 | 54358390 | 39339190 |
| A81 | Schizophrenia | 8.6 | 101 | 43237814 | 29716763 |
| A82 | Schizophrenia | 7.9 | 101 | 42808428 | 30372069 |
| A85 | Schizophrenia | 8.3 | 101 | 51357479 | 35214983 |
| A87 | Schizophrenia | 8.4 | 101 | 42160239 | 29970947 |
| A88 | Schizophrenia | 8.2 | 101 | 56863970 | 43316852 |
| A92 | Schizophrenia | 7.4 | 101 | 44356460 | 31161734 |
| A93 | Schizophrenia | 8 | 101 | 51094747 | 37564119 |
| A94 | Schizophrenia | 7.7 | 101 | 52358816 | 37201708 |
| A97 | Schizophrenia | 7.4 | 101 | 48108867 | 33030855 |
| A100 | Schizophrenia | 8.6 | 101 | 50454082 | 38256895 |
| A15 | Normal | 8.3 | 101 | 57428290 | 41539201 |
| A19 | Normal | 8.4 | 101 | 52592402 | 38266030 |
| A24 | Normal | 7.8 | 101 | 56597930 | 40482830 |
| A27 | Normal | 7.8 | 101 | 58393700 | 41961409 |
| A29 | Normal | 8.4 | 101 | 67489727 | 51000499 |
| A30 | Normal | 7.6 | 101 | 54904122 | 38756549 |
| A33 | Normal | 7.3 | 101 | 59406292 | 43879509 |
| A35 | Normal | 7.7 | 101 | 53266199 | 37031092 |
| A38 | Normal | 7.7 | 101 | 53622333 | 37682313 |
| A43 | Normal | 7.9 | 101 | 56981674 | 42743670 |
| A56 | Normal | 8.6 | 101 | 59231221 | 38816232 |
| A59 | Normal | 7.9 | 101 | 49938999 | 35761353 |
| A60 | Normal | 8 | 101 | 50023948 | 32716541 |
| A65 | Normal | 8 | 101 | 43192561 | 31632848 |
| A67 | Normal | 7.6 | 101 | 47717198 | 34227278 |
| A70 | Normal | 7.8 | 101 | 41893833 | 31014064 |
| A71 | Normal | 7.3 | 101 | 47381589 | 34358426 |
| A80 | Normal | 8.6 | 101 | 47438149 | 34376615 |
| A83 | Normal | 8 | 101 | 37944489 | 27664574 |
| A84 | Normal | 8.4 | 101 | 40426876 | 29997783 |
| A86 | Normal | 7.7 | 101 | 44907368 | 33518889 |
| A91 | Normal | 8.1 | 101 | 55468625 | 40807284 |
| A95 | Normal | 8.4 | 101 | 62110770 | 45977263 |
| A96 | Normal | 8.5 | 101 | 49323872 | 35073381 |
| A104 | Normal | 8.1 | 101 | 47045703 | 36524823 |
| A105 | Normal | 7 | 101 | 41597000 | 32706820 |
|  |  |  | **Mean** | 51023315 | 36678552 |

^1^Mapping to a reference genome (hg19)

**Supplementary Table 3.** Genes differentially expressed between schizophrenia and controls in the choroid plexus

| **Gene** | **log FC^1^** | **log CPM^2^** | **P Value** | **FDR** |
| --- | --- | --- | --- | --- |
| *ADAMTS17* | 1.302319 | 3.269767 | 1.70E-08 | 0.000287 |
| *STEAP4* | 2.086322 | 6.676885 | 5.02E-08 | 0.000425 |
| *PTX3* | 2.847839 | 2.560459 | 1.04E-07 | 0.000494 |
| *SPOCD1* | 3.133771 | 0.550966 | 1.17E-07 | 0.000494 |
| *PLA2G2A* | 3.37964 | 4.52676 | 9.18E-07 | 0.003104 |
| *GPR87* | 3.450984 | 0.191461 | 1.27E-06 | 0.003566 |
| *CYP1B1* | 1.737682 | 6.87264 | 2.14E-06 | 0.005163 |
| *CLEC4G* | 1.851606 | 0.298014 | 3.23E-06 | 0.006829 |
| *SERPINA3* | 1.716174 | 7.368375 | 4.26E-06 | 0.007042 |
| *LRG1* | 1.839762 | -0.02212 | 4.36E-06 | 0.007042 |
| *SLC11A1* | 1.479476 | 4.15724 | 4.58E-06 | 0.007042 |
| *OSMR* | 1.069304 | 5.613205 | 1.16E-05 | 0.016181 |
| *SLC1A1* | 1.044165 | 4.670346 | 1.24E-05 | 0.016181 |
| *CALCB* | -1.73738 | -0.67107 | 1.44E-05 | 0.01736 |
| *CD163* | 1.372911 | 6.54001 | 1.55E-05 | 0.017501 |
| *LOX* | 1.26659 | 4.459728 | 1.89E-05 | 0.019798 |
| *ADAMTSL4* | 0.728821 | 5.428531 | 1.99E-05 | 0.019798 |
| *NEXN-AS1* | -1.34331 | 0.147018 | 2.15E-05 | 0.019936 |
| *SIGLEC12* | 2.15361 | -1.46837 | 2.24E-05 | 0.019936 |
| *CLEC4D* | 2.422148 | -1.65313 | 2.76E-05 | 0.023313 |
| *TNFSF14* | 1.9194 | 0.523175 | 2.91E-05 | 0.02344 |
| *THBS1* | 1.777968 | 6.154868 | 3.18E-05 | 0.024442 |
| *TMEM260* | -0.47755 | 6.637162 | 3.51E-05 | 0.025369 |
| *OTOG* | 2.130089 | -0.9318 | 3.60E-05 | 0.025369 |
| *CISH* | 0.608832 | 2.562709 | 5.94E-05 | 0.040189 |
| *IL18R1* | 1.347234 | 2.153799 | 6.24E-05 | 0.040565 |
| *MARCO* | -1.50303 | 3.140391 | 6.55E-05 | 0.040989 |

logFC, log2 fold change ; logCPM, log2 counts-per-million

**Supplementary Table 4.**  Biological processes significantly enriched in the upregulated genes in CP of schizophrenia

| **Term** | **Count** | **PValue** |
| --- | --- | --- |
| GO:0006952~defense response | 7 | 2.6E-04 |
| GO:0009611~response to wounding | 6 | 1.1E-03 |
| GO:0006954~inflammatory response | 5 | 1.4E-03 |
| GO:0006909~phagocytosis | 3 | 2.5E-03 |
| GO:0002604~regulation of dendritic cell antigen processing and presentation | 2 | 3.1E-03 |
| GO:0002577~regulation of antigen processing and presentation | 2 | 3.1E-03 |
| GO:0006955~immune response | 6 | 3.5E-03 |
| GO:0032103~positive regulation of response to external stimulus | 3 | 4.4E-03 |
| GO:0048584~positive regulation of response to stimulus | 4 | 5.5E-03 |
| GO:0043090~amino acid import | 2 | 1.2E-02 |
| GO:0043092~L-amino acid import | 2 | 1.2E-02 |
| GO:0050766~positive regulation of phagocytosis | 2 | 1.8E-02 |
| GO:0010033~response to organic substance | 5 | 2.3E-02 |
| GO:0050764~regulation of phagocytosis | 2 | 2.5E-02 |
| GO:0032101~regulation of response to external stimulus | 3 | 2.5E-02 |
| GO:0015807~L-amino acid transport | 2 | 3.5E-02 |
| GO:0042098~T cell proliferation | 2 | 4.1E-02 |
| GO:0006826~iron ion transport | 2 | 4.4E-02 |
| GO:0045807~positive regulation of endocytosis | 2 | 4.4E-02 |
| GO:0010324~membrane invagination | 3 | 4.5E-02 |
| GO:0006897~endocytosis | 3 | 4.5E-02 |
| GO:0050729~positive regulation of inflammatory response | 2 | 4.6E-02 |
| GO:0051050~positive regulation of transport | 3 | 4.6E-02 |
| GO:0045860~positive regulation of protein kinase activity | 3 | 4.6E-02 |
| GO:0033674~positive regulation of kinase activity | 3 | 4.9E-02 |

**Supplementary Table 5.** Correlation coefficients between co-expression modules and descriptive variables

| **Module** | **Schizophrenia** | **Age** | **Sex** | **PMI** | **Brain pH** | **Antipsy**  **-chotics** | **RIN** |
| --- | --- | --- | --- | --- | --- | --- | --- |
| S_M1 | ns | ns | ns | ns | ns | ns | -0.4  (P_adj_=0.02) |
| S_M2 | ns | ns | ns | ns | ns | ns | ns |
| S_M3 | ns | ns | ns | ns | ns | ns | ns |
| S_M4 | ns | ns | ns | ns | ns | ns | ns |
| S_M5 | ns | ns | ns | ns | ns | ns | ns |
| S_M6 | ns | ns | ns | ns | ns | ns | ns |
| S_M7 | ns | ns | ns | ns | ns | ns | ns |
| S_M8 | ns | ns | ns | ns | ns | ns | ns |
| S_M9 | ns | ns | ns | ns | ns | ns | 0.5  (P_adj_=0.002) |
| _M10 | ns | ns | ns | ns | ns | ns | ns |
| S_M11 | ns | ns | ns | ns | ns | ns | ns |
| S_M12 | ns | ns | ns | ns | ns | ns | ns |
| S_M13 | ns | ns | ns | ns | ns | ns | ns |
| S_M14 | ns | ns | ns | ns | ns | ns | ns |
| S_M15 | ns | ns | ns | ns | ns | ns | ns |
| S_M16 | 0.3  (P_adj_=0.26) | ns | ns | ns | ns | ns | ns |
| S_M17 | ns | ns | ns | ns | ns | ns | ns |
| S_M18 | ns | ns | ns | ns | ns | ns | ns |

**Supplementary Table 6.**  Biological processes enriched in the genes in the S_M16 module

| **Term** | **Count** | **PValue** |
| --- | --- | --- |
| GO:0006952~defense response | 57 | 4.25E-11 |
| GO:0009611~response to wounding | 52 | 4.59E-11 |
| GO:0006954~inflammatory response | 37 | 9.23E-10 |
| GO:0006955~immune response | 58 | 1.18E-09 |
| GO:0043065~positive regulation of apoptosis | 39 | 1.52E-07 |
| GO:0043068~positive regulation of programmed cell death | 39 | 1.82E-07 |
| GO:0010942~positive regulation of cell death | 39 | 2.05E-07 |
| GO:0009615~response to virus | 18 | 2.26E-07 |
| GO:0042127~regulation of cell proliferation | 56 | 6.80E-07 |
| GO:0002684~positive regulation of immune system process | 26 | 9.85E-07 |
| GO:0010033~response to organic substance | 51 | 2.88E-06 |
| GO:0048584~positive regulation of response to stimulus | 25 | 2.88E-06 |
| GO:0002683~negative regulation of immune system process | 14 | 5.73E-06 |
| GO:0042981~regulation of apoptosis | 54 | 6.32E-06 |
| GO:0043067~regulation of programmed cell death | 54 | 8.26E-06 |
| GO:0010941~regulation of cell death | 54 | 9.20E-06 |
| GO:0001501~skeletal system development | 28 | 2.18E-05 |
| GO:0002237~response to molecule of bacterial origin | 13 | 4.32E-05 |
| GO:0031349~positive regulation of defense response | 12 | 4.35E-05 |
| GO:0050778~positive regulation of immune response | 17 | 4.84E-05 |
| GO:0006917~induction of apoptosis | 27 | 6.19E-05 |
| GO:0012502~induction of programmed cell death | 27 | 6.53E-05 |
| GO:0008283~cell proliferation | 33 | 6.62E-05 |
| GO:0001817~regulation of cytokine production | 19 | 6.67E-05 |
| GO:0002252~immune effector process | 16 | 7.03E-05 |
| GO:0002460~adaptive immune response based on somatic recombination of immune receptors built from immunoglobulin superfamily domains | 12 | 7.19E-05 |
| GO:0002250~adaptive immune response | 12 | 7.19E-05 |
| GO:0045596~negative regulation of cell differentiation | 21 | 7.42E-05 |
| GO:0034097~response to cytokine stimulus | 12 | 9.14E-05 |
| GO:0045087~innate immune response | 16 | 9.85E-05 |
| GO:0009967~positive regulation of signal transduction | 25 | 1.16E-04 |
| GO:0019724~B cell mediated immunity | 10 | 1.29E-04 |
| GO:0051270~regulation of cell motion | 19 | 1.52E-04 |
| GO:0008285~negative regulation of cell proliferation | 28 | 1.80E-04 |
| GO:0010647~positive regulation of cell communication | 26 | 2.44E-04 |
| GO:0051329~interphase of mitotic cell cycle | 13 | 2.52E-04 |
| GO:0030334~regulation of cell migration | 17 | 2.95E-04 |
| GO:0051094~positive regulation of developmental process | 23 | 3.27E-04 |
| GO:0051325~interphase | 13 | 3.30E-04 |
| GO:0032496~response to lipopolysaccharide | 11 | 3.39E-04 |
| GO:0032695~negative regulation of interleukin-12 production | 4 | 4.06E-04 |
| GO:0002253~activation of immune response | 12 | 4.36E-04 |
| GO:0001558~regulation of cell growth | 18 | 4.79E-04 |
| GO:0016064~immunoglobulin mediated immune response | 9 | 5.40E-04 |
| GO:0002822~regulation of adaptive immune response based on somatic recombination of immune receptors built from immunoglobulin superfamily domains | 9 | 6.13E-04 |
| GO:0002526~acute inflammatory response | 12 | 6.24E-04 |
| GO:0002455~humoral immune response mediated by circulating immunoglobulin | 7 | 6.26E-04 |
| GO:0051726~regulation of cell cycle | 25 | 6.37E-04 |
| GO:0002819~regulation of adaptive immune response | 9 | 6.93E-04 |
| GO:0000082~G1/S transition of mitotic cell cycle | 9 | 6.93E-04 |
| GO:0008284~positive regulation of cell proliferation | 29 | 7.03E-04 |
| GO:0002449~lymphocyte mediated immunity | 10 | 7.18E-04 |
| GO:0050678~regulation of epithelial cell proliferation | 10 | 7.98E-04 |
| GO:0002443~leukocyte mediated immunity | 11 | 8.27E-04 |
| GO:0040008~regulation of growth | 25 | 9.65E-04 |
| GO:0010810~regulation of cell-substrate adhesion | 8 | 0.001015 |
| GO:0045785~positive regulation of cell adhesion | 9 | 0.001105 |
| GO:0040012~regulation of locomotion | 17 | 0.001193 |
| GO:0045597~positive regulation of cell differentiation | 19 | 0.001202 |
| GO:0009725~response to hormone stimulus | 26 | 0.001218 |
| GO:0050865~regulation of cell activation | 16 | 0.001268 |
| GO:0070482~response to oxygen levels | 14 | 0.00136 |
| GO:0007242~intracellular signaling cascade | 65 | 0.001394 |
| GO:0001944~vasculature development | 20 | 0.001406 |
| GO:0022610~biological adhesion | 41 | 0.001607 |
| GO:0007155~cell adhesion | 41 | 0.00162 |
| GO:0009894~regulation of catabolic process | 11 | 0.001932 |
| GO:0043470~regulation of carbohydrate catabolic process | 5 | 0.001936 |
| GO:0043471~regulation of cellular carbohydrate catabolic process | 5 | 0.001936 |
| GO:0009991~response to extracellular stimulus | 18 | 0.001952 |
| GO:0045786~negative regulation of cell cycle | 10 | 0.002054 |
| GO:0010605~negative regulation of macromolecule metabolic process | 42 | 0.002078 |
| GO:0051893~regulation of focal adhesion formation | 4 | 0.002098 |
| GO:0051249~regulation of lymphocyte activation | 14 | 0.002105 |
| GO:0002694~regulation of leukocyte activation | 15 | 0.002117 |
| GO:0051052~regulation of DNA metabolic process | 12 | 0.002173 |
| GO:0009719~response to endogenous stimulus | 27 | 0.002233 |
| GO:0010740~positive regulation of protein kinase cascade | 15 | 0.002239 |
| GO:0001503~ossification | 12 | 0.002329 |
| GO:0051240~positive regulation of multicellular organismal process | 19 | 0.002435 |
| GO:0070555~response to interleukin-1 | 5 | 0.002462 |
| GO:0001568~blood vessel development | 19 | 0.002545 |
| GO:0001666~response to hypoxia | 13 | 0.002623 |
| GO:0006928~cell motion | 30 | 0.00263 |
| GO:0002706~regulation of lymphocyte mediated immunity | 8 | 0.002642 |
| GO:0051250~negative regulation of lymphocyte activation | 8 | 0.002642 |
| GO:0007243~protein kinase cascade | 25 | 0.002872 |
| GO:0060341~regulation of cellular localization | 19 | 0.002902 |
| GO:0006958~complement activation, classical pathway | 6 | 0.00306 |
| GO:0001914~regulation of T cell mediated cytotoxicity | 4 | 0.003066 |
| GO:0051605~protein maturation by peptide bond cleavage | 10 | 0.003106 |
| GO:0048545~response to steroid hormone stimulus | 16 | 0.00314 |
| GO:0030155~regulation of cell adhesion | 13 | 0.003151 |
| GO:0006956~complement activation | 7 | 0.003222 |
| GO:0009617~response to bacterium | 16 | 0.003299 |
| GO:0002695~negative regulation of leukocyte activation | 8 | 0.003605 |
| GO:0002541~activation of plasma proteins involved in acute inflammatory response | 7 | 0.003636 |
| GO:0007584~response to nutrient | 13 | 0.003761 |
| GO:0032101~regulation of response to external stimulus | 14 | 0.003931 |
| GO:0060348~bone development | 12 | 0.003934 |
| GO:0032680~regulation of tumor necrosis factor production | 6 | 0.004134 |
| GO:0001953~negative regulation of cell-matrix adhesion | 4 | 0.004267 |
| GO:0030308~negative regulation of cell growth | 10 | 0.004883 |
| GO:0050727~regulation of inflammatory response | 9 | 0.00501 |
| GO:0045089~positive regulation of innate immune response | 7 | 0.005115 |
| GO:0048660~regulation of smooth muscle cell proliferation | 7 | 0.005115 |
| GO:0045926~negative regulation of growth | 11 | 0.005212 |
| GO:0002703~regulation of leukocyte mediated immunity | 8 | 0.005279 |
| GO:0050866~negative regulation of cell activation | 8 | 0.005279 |
| GO:0031329~regulation of cellular catabolic process | 8 | 0.005279 |
| GO:0006357~regulation of transcription from RNA polymerase II promoter | 40 | 0.00529 |
| GO:0010812~negative regulation of cell-substrate adhesion | 4 | 0.005716 |
| GO:0010558~negative regulation of macromolecule biosynthetic process | 32 | 0.005843 |
| GO:0016485~protein processing | 11 | 0.005914 |
| GO:0018212~peptidyl-tyrosine modification | 7 | 0.00632 |
| GO:0009890~negative regulation of biosynthetic process | 33 | 0.006493 |
| GO:0032844~regulation of homeostatic process | 11 | 0.006687 |
| GO:0051174~regulation of phosphorus metabolic process | 29 | 0.006725 |
| GO:0019220~regulation of phosphate metabolic process | 29 | 0.006725 |
| GO:0010627~regulation of protein kinase cascade | 18 | 0.006886 |
| GO:0006198~cAMP catabolic process | 3 | 0.007014 |
| GO:0051272~positive regulation of cell motion | 10 | 0.007365 |
| GO:0042060~wound healing | 15 | 0.007399 |
| GO:0014068~positive regulation of phosphoinositide 3-kinase cascade | 4 | 0.007425 |
| GO:0042325~regulation of phosphorylation | 28 | 0.007469 |
| GO:0045792~negative regulation of cell size | 10 | 0.007858 |
| GO:0031327~negative regulation of cellular biosynthetic process | 32 | 0.008214 |
| GO:0016477~cell migration | 19 | 0.008715 |
| GO:0002697~regulation of immune effector process | 10 | 0.00892 |
| GO:0051172~negative regulation of nitrogen compound metabolic process | 30 | 0.009028 |
| GO:0007167~enzyme linked receptor protein signaling pathway | 22 | 0.009375 |
| GO:0014066~regulation of phosphoinositide 3-kinase cascade | 4 | 0.009404 |
| GO:0031667~response to nutrient levels | 15 | 0.009594 |
| GO:0001818~negative regulation of cytokine production | 6 | 0.009999 |
| GO:0051101~regulation of DNA binding | 11 | 0.010026 |
| GO:0045444~fat cell differentiation | 7 | 0.010216 |
| GO:0007166~cell surface receptor linked signal transduction | 84 | 0.010464 |
| GO:0051604~protein maturation | 11 | 0.010592 |
| GO:0030198~extracellular matrix organization | 10 | 0.010711 |
| GO:0043388~positive regulation of DNA binding | 8 | 0.011098 |
| GO:0019221~cytokine-mediated signaling pathway | 8 | 0.011098 |
| GO:0007613~memory | 6 | 0.011148 |
| GO:0045088~regulation of innate immune response | 7 | 0.011162 |
| GO:0009968~negative regulation of signal transduction | 16 | 0.011321 |
| GO:0051895~negative regulation of focal adhesion formation | 3 | 0.011419 |
| GO:0030335~positive regulation of cell migration | 9 | 0.012682 |
| GO:0001775~cell activation | 19 | 0.012685 |
| GO:0000122~negative regulation of transcription from RNA polymerase II promoter | 18 | 0.012839 |
| GO:0045934~negative regulation of nucleobase, nucleoside, nucleotide and nucleic acid metabolic process | 29 | 0.013341 |
| GO:0001819~positive regulation of cytokine production | 9 | 0.013508 |
| GO:0001952~regulation of cell-matrix adhesion | 5 | 0.013779 |
| GO:0010648~negative regulation of cell communication | 17 | 0.014251 |
| GO:0030336~negative regulation of cell migration | 7 | 0.014372 |
| GO:0045787~positive regulation of cell cycle | 7 | 0.014372 |
| GO:0007565~female pregnancy | 10 | 0.015088 |
| GO:0022405~hair cycle process | 6 | 0.015125 |
| GO:0022404~molting cycle process | 6 | 0.015125 |
| GO:0001942~hair follicle development | 6 | 0.015125 |
| GO:0012501~programmed cell death | 33 | 0.015318 |
| GO:0010811~positive regulation of cell-substrate adhesion | 5 | 0.015641 |
| GO:0050867~positive regulation of cell activation | 10 | 0.01593 |
| GO:0042303~molting cycle | 6 | 0.016636 |
| GO:0042633~hair cycle | 6 | 0.016636 |
| GO:0009214~cyclic nucleotide catabolic process | 3 | 0.016733 |
| GO:0051797~regulation of hair follicle development | 3 | 0.016733 |
| GO:0042634~regulation of hair cycle | 3 | 0.016733 |
| GO:0002755~MyD88-dependent toll-like receptor signaling pathway | 3 | 0.016733 |
| GO:0002521~leukocyte differentiation | 11 | 0.016828 |
| GO:0048514~blood vessel morphogenesis | 15 | 0.016928 |
| GO:0051091~positive regulation of transcription factor activity | 7 | 0.018174 |
| GO:0002699~positive regulation of immune effector process | 6 | 0.018245 |
| GO:0001932~regulation of protein amino acid phosphorylation | 13 | 0.018776 |
| GO:0010629~negative regulation of gene expression | 28 | 0.01882 |
| GO:0051098~regulation of binding | 12 | 0.018829 |
| GO:0051099~positive regulation of binding | 8 | 0.019304 |
| GO:0051384~response to glucocorticoid stimulus | 8 | 0.019304 |
| GO:0030097~hemopoiesis | 16 | 0.0195 |
| GO:0040013~negative regulation of locomotion | 7 | 0.01958 |
| GO:0045859~regulation of protein kinase activity | 21 | 0.019581 |
| GO:0043434~response to peptide hormone stimulus | 12 | 0.019663 |
| GO:0043467~regulation of generation of precursor metabolites and energy | 5 | 0.019821 |
| GO:0002824~positive regulation of adaptive immune response based on somatic recombination of immune receptors built from immunoglobulin superfamily domains | 5 | 0.019821 |
| GO:0002221~pattern recognition receptor signaling pathway | 4 | 0.020158 |
| GO:0032655~regulation of interleukin-12 production | 4 | 0.020158 |
| GO:0006959~humoral immune response | 8 | 0.020565 |
| GO:0006915~apoptosis | 32 | 0.020591 |
| GO:0048534~hemopoietic or lymphoid organ development | 17 | 0.02132 |
| GO:0040017~positive regulation of locomotion | 9 | 0.021581 |
| GO:0018108~peptidyl-tyrosine phosphorylation | 6 | 0.021763 |
| GO:0002821~positive regulation of adaptive immune response | 5 | 0.022145 |
| GO:0051338~regulation of transferase activity | 22 | 0.022153 |
| GO:0045619~regulation of lymphocyte differentiation | 7 | 0.022612 |
| GO:0051271~negative regulation of cell motion | 7 | 0.022612 |
| GO:0032494~response to peptidoglycan | 3 | 0.022888 |
| GO:0001916~positive regulation of T cell mediated cytotoxicity | 3 | 0.022888 |
| GO:0051674~localization of cell | 19 | 0.023438 |
| GO:0048870~cell motility | 19 | 0.023438 |
| GO:0050869~negative regulation of B cell activation | 4 | 0.023574 |
| GO:0045321~leukocyte activation | 16 | 0.023888 |
| GO:0048585~negative regulation of response to stimulus | 9 | 0.02404 |
| GO:0032868~response to insulin stimulus | 9 | 0.02404 |
| GO:0032103~positive regulation of response to external stimulus | 7 | 0.02424 |
| GO:0005996~monosaccharide metabolic process | 15 | 0.025146 |
| GO:0042330~taxis | 12 | 0.025256 |
| GO:0006935~chemotaxis | 12 | 0.025256 |
| GO:0051046~regulation of secretion | 14 | 0.025735 |
| GO:0050670~regulation of lymphocyte proliferation | 8 | 0.026183 |
| GO:0043405~regulation of MAP kinase activity | 11 | 0.026562 |
| GO:0043549~regulation of kinase activity | 21 | 0.027018 |
| GO:0045639~positive regulation of myeloid cell differentiation | 5 | 0.027273 |
| GO:0002758~innate immune response-activating signal transduction | 4 | 0.027283 |
| GO:0002218~activation of innate immune response | 4 | 0.027283 |
| GO:0033273~response to vitamin | 7 | 0.027728 |
| GO:0046883~regulation of hormone secretion | 7 | 0.027728 |
| GO:0070663~regulation of leukocyte proliferation | 8 | 0.027737 |
| GO:0032944~regulation of mononuclear cell proliferation | 8 | 0.027737 |
| GO:0051090~regulation of transcription factor activity | 9 | 0.028088 |
| GO:0043062~extracellular structure organization | 12 | 0.028448 |
| GO:0031960~response to corticosteroid stimulus | 8 | 0.029351 |
| GO:0042698~ovulation cycle | 7 | 0.029589 |
| GO:0008361~regulation of cell size | 14 | 0.029978 |
| GO:0015718~monocarboxylic acid transport | 6 | 0.030052 |
| GO:0002708~positive regulation of lymphocyte mediated immunity | 5 | 0.030081 |
| GO:0002705~positive regulation of leukocyte mediated immunity | 5 | 0.030081 |
| GO:0050830~defense response to Gram-positive bacterium | 4 | 0.031283 |
| GO:0019048~virus-host interaction | 4 | 0.031283 |
| GO:0032651~regulation of interleukin-1 beta production | 4 | 0.031283 |
| GO:0032570~response to progesterone stimulus | 4 | 0.031283 |
| GO:0050864~regulation of B cell activation | 6 | 0.032396 |
| GO:0002696~positive regulation of leukocyte activation | 9 | 0.032585 |
| GO:0010906~regulation of glucose metabolic process | 5 | 0.033053 |
| GO:0006793~phosphorus metabolic process | 46 | 0.033214 |
| GO:0006796~phosphate metabolic process | 46 | 0.033214 |
| GO:0016051~carbohydrate biosynthetic process | 9 | 0.034187 |
| GO:0002520~immune system development | 17 | 0.03449 |
| GO:0001525~angiogenesis | 11 | 0.03542 |
| GO:0002709~regulation of T cell mediated immunity | 4 | 0.035573 |
| GO:0043029~T cell homeostasis | 4 | 0.035573 |
| GO:0045637~regulation of myeloid cell differentiation | 7 | 0.035659 |
| GO:0032675~regulation of interleukin-6 production | 5 | 0.036191 |
| GO:0052200~response to host defenses | 3 | 0.037462 |
| GO:0009125~nucleoside monophosphate catabolic process | 3 | 0.037462 |
| GO:0044003~modification by symbiont of host morphology or physiology | 3 | 0.037462 |
| GO:0052173~response to defenses of other organism during symbiotic interaction | 3 | 0.037462 |
| GO:0075136~response to host | 3 | 0.037462 |
| GO:0010717~regulation of epithelial to mesenchymal transition | 3 | 0.037462 |
| GO:0010038~response to metal ion | 10 | 0.037544 |
| GO:0019318~hexose metabolic process | 13 | 0.037967 |
| GO:0045682~regulation of epidermis development | 4 | 0.04015 |
| GO:0045930~negative regulation of mitotic cell cycle | 4 | 0.04015 |
| GO:0022604~regulation of cell morphogenesis | 10 | 0.04079 |
| GO:0006006~glucose metabolic process | 11 | 0.042886 |
| GO:0051592~response to calcium ion | 6 | 0.042887 |
| GO:0010675~regulation of cellular carbohydrate metabolic process | 5 | 0.042965 |
| GO:0006468~protein amino acid phosphorylation | 33 | 0.043087 |
| GO:0006470~protein amino acid dephosphorylation | 10 | 0.044222 |
| GO:0032870~cellular response to hormone stimulus | 10 | 0.044222 |
| GO:0016311~dephosphorylation | 11 | 0.044499 |
| GO:0051216~cartilage development | 7 | 0.044919 |
| GO:0006071~glycerol metabolic process | 4 | 0.045008 |
| GO:0032652~regulation of interleukin-1 production | 4 | 0.045008 |
| GO:0045622~regulation of T-helper cell differentiation | 3 | 0.04576 |
| GO:0032925~regulation of activin receptor signaling pathway | 3 | 0.04576 |
| GO:0070227~lymphocyte apoptosis | 3 | 0.04576 |
| GO:0032583~regulation of gene-specific transcription | 10 | 0.046008 |
| GO:0045892~negative regulation of transcription, DNA-dependent | 20 | 0.046384 |
| GO:0006109~regulation of carbohydrate metabolic process | 5 | 0.046601 |
| GO:0051053~negative regulation of DNA metabolic process | 5 | 0.046601 |
| GO:0051223~regulation of protein transport | 9 | 0.046907 |
| GO:0008219~cell death | 35 | 0.047302 |
| GO:0046649~lymphocyte activation | 13 | 0.047669 |
| GO:0034330~cell junction organization | 6 | 0.048815 |
| GO:0016265~death | 35 | 0.048828 |

**Supplementary Table 7.** Module preservation statistics for schizophrenia and controls

| CTL to schizophrenia | | schizophrenia to CTL | |
| --- | --- | --- | --- |
| Module | Z _summary_ | Module | Z _summary_ |
| C_only1_M1 | 20 | SCH_only_M1 | 18 |
| C_only1_M2 | 50 | SCH_only_M2 | 48 |
| C_only1_M3 | 26 | SCH_only_M3 | 11 |
| C_only1_M4 | 19 | SCH_only_M4 | 21 |
| C_only1_M5 | 14 | SCH_only_M5 | 17 |
| C_only1_M6 | 15 | SCH_only_M6 | 65 |
| C_only1_M7 | 13 | SCH_only_M7 | **5.5** |
| C_only1_M8 | **5.5** |  |  |
| C_only1_M9 | 67 |  |  |
| C_only1_M10 | 22 |  |  |

**Supplementary Table 8.** Correlation coefficients between co-expression modules and descriptive variables.

| **Module** | **Age** | **Sex** | **PMI** | **Brain pH** | **RIN** |
| --- | --- | --- | --- | --- | --- |
| C_only1_M1 | ns | ns | ns | ns | ns |
| C_only1_M2 | ns | ns | ns | ns | ns |
| C_only1_M3 | ns | ns | ns | ns | ns |
| C_only1_M4 | ns | ns | ns | ns | ns |
| C_only1_M5 | ns | ns | ns | ns | ns |
| C_only1_M6 | ns | ns | ns | ns | ns |
| C_only1_M7 | ns | ns | ns | ns | 0.58 (Padj=0.01) |
| C_only1_M8 | ns | ns | ns | -0.49 (Padj=0.01) | ns |
| C_only1_M9 | ns | ns | ns | ns | ns |
| C_only1_M10 | ns | ns | ns | ns | ns |

**Supplementary Table 9.**  Biological processes enriched in the genes in the C_only1_M8 module

| **Term** | **Count** | **PValue** |
| --- | --- | --- |
| GO:0030278~regulation of ossification | 4 | 0.001148 |
| GO:0045944~positive regulation of transcription from RNA polymerase II promoter | 6 | 0.002827 |
| GO:0045667~regulation of osteoblast differentiation | 3 | 0.005806 |
| GO:0051094~positive regulation of developmental process | 5 | 0.006129 |
| GO:0042325~regulation of phosphorylation | 6 | 0.007412 |
| GO:0009967~positive regulation of signal transduction | 5 | 0.007536 |
| GO:0045893~positive regulation of transcription, DNA-dependent | 6 | 0.00816 |
| GO:0051254~positive regulation of RNA metabolic process | 6 | 0.008444 |
| GO:0019220~regulation of phosphate metabolic process | 6 | 0.008736 |
| GO:0051174~regulation of phosphorus metabolic process | 6 | 0.008736 |
| GO:0010740~positive regulation of protein kinase cascade | 4 | 0.009787 |
| GO:0010647~positive regulation of cell communication | 5 | 0.01096 |
| GO:0051726~regulation of cell cycle | 5 | 0.011188 |
| GO:0006357~regulation of transcription from RNA polymerase II promoter | 7 | 0.011551 |
| GO:0007167~enzyme linked receptor protein signaling pathway | 5 | 0.012498 |
| GO:0045941~positive regulation of transcription | 6 | 0.016019 |
| GO:0010628~positive regulation of gene expression | 6 | 0.017999 |
| GO:0009968~negative regulation of signal transduction | 4 | 0.020665 |
| GO:0045597~positive regulation of cell differentiation | 4 | 0.022673 |
| GO:0001934~positive regulation of protein amino acid phosphorylation | 3 | 0.023326 |
| GO:0008284~positive regulation of cell proliferation | 5 | 0.023519 |
| GO:0045935~positive regulation of nucleobase, nucleoside, nucleotide and nucleic acid metabolic process | 6 | 0.023722 |
| GO:0033138~positive regulation of peptidyl-serine phosphorylation | 2 | 0.026304 |
| GO:0051173~positive regulation of nitrogen compound metabolic process | 6 | 0.026748 |
| GO:0042327~positive regulation of phosphorylation | 3 | 0.027369 |
| GO:0010648~negative regulation of cell communication | 4 | 0.027858 |
| GO:0010627~regulation of protein kinase cascade | 4 | 0.028147 |
| GO:0010557~positive regulation of macromolecule biosynthetic process | 6 | 0.028351 |
| GO:0060324~face development | 2 | 0.028897 |
| GO:0042517~positive regulation of tyrosine phosphorylation of Stat3 protein | 2 | 0.028897 |
| GO:0045937~positive regulation of phosphate metabolic process | 3 | 0.028954 |
| GO:0010562~positive regulation of phosphorus metabolic process | 3 | 0.028954 |
| GO:0007389~pattern specification process | 4 | 0.033626 |
| GO:0031328~positive regulation of cellular biosynthetic process | 6 | 0.033709 |
| GO:0009891~positive regulation of biosynthetic process | 6 | 0.035565 |
| GO:0060322~head development | 2 | 0.036636 |
| GO:0033135~regulation of peptidyl-serine phosphorylation | 2 | 0.036636 |
| GO:0006355~regulation of transcription, DNA-dependent | 10 | 0.038863 |
| GO:0051252~regulation of RNA metabolic process | 10 | 0.043985 |
| GO:0042516~regulation of tyrosine phosphorylation of Stat3 protein | 2 | 0.044315 |
| GO:0035239~tube morphogenesis | 3 | 0.044764 |
| GO:0048511~rhythmic process | 3 | 0.0454 |
| GO:0044057~regulation of system process | 4 | 0.048415 |
| GO:0048008~platelet-derived growth factor receptor signaling pathway | 2 | 0.049401 |

**Supplementary Table 10.** Correlation coefficients between co-expression modules and descriptive variables.

| **Module** | **Age** | **Sex** | **PMI** | **Brain pH** | **LifetimeAntipsychotics** | **RIN** |
| --- | --- | --- | --- | --- | --- | --- |
| SCH_only_M1 | ns | ns | ns | ns | ns | ns |
| SCH_only_M2 | ns | ns | ns | -0.5 (Padj=0.03) | ns | ns |
| SCH_only_M3 | ns | ns | ns | ns | ns | ns |
| SCH_only_M4 | ns | ns | ns | ns | ns | -0.5 (Padj=0.02) |
| SCH_only_M5 | ns | ns | ns | ns | ns | ns |
| SCH_only_M6 | ns | ns | ns | ns | ns | ns |
| SCH_only_M7 | ns | ns | ns | ns | ns | ns |

**Supplementary Table 11.** Biological processes enriched in the genes in the SCH_only_M7

| **Term** | **Count** | **PValue** |
| --- | --- | --- |
| GO:0006986~response to unfolded protein | 13 | 2.20E-18 |
| GO:0051789~response to protein stimulus | 13 | 3.91E-16 |
| GO:0006457~protein folding | 13 | 1.86E-13 |
| GO:0010033~response to organic substance | 16 | 2.58E-09 |
| GO:0010941~regulation of cell death | 11 | 1.85E-04 |
| GO:0042981~regulation of apoptosis | 10 | 8.01E-04 |
| GO:0043067~regulation of programmed cell death | 10 | 8.60E-04 |
| GO:0008285~negative regulation of cell proliferation | 7 | 9.17E-04 |
| GO:0043066~negative regulation of apoptosis | 6 | 0.005076 |
| GO:0043069~negative regulation of programmed cell death | 6 | 0.005384 |
| GO:0060548~negative regulation of cell death | 6 | 0.005447 |
| GO:0010942~positive regulation of cell death | 6 | 0.011837 |
| GO:0043623~cellular protein complex assembly | 4 | 0.014656 |
| GO:0042026~protein refolding | 2 | 0.015795 |
| GO:0051131~chaperone-mediated protein complex assembly | 2 | 0.022044 |
| GO:0043281~regulation of caspase activity | 3 | 0.026052 |
| GO:0045786~negative regulation of cell cycle | 3 | 0.027288 |
| GO:0006916~anti-apoptosis | 4 | 0.027488 |
| GO:0052548~regulation of endopeptidase activity | 3 | 0.027915 |
| GO:0009628~response to abiotic stimulus | 5 | 0.028886 |
| GO:0052547~regulation of peptidase activity | 3 | 0.030479 |
| GO:0042127~regulation of cell proliferation | 7 | 0.036917 |
| GO:0006915~apoptosis | 6 | 0.041022 |
| GO:0012501~programmed cell death | 6 | 0.04328 |
| GO:0043065~positive regulation of apoptosis | 5 | 0.046906 |
| GO:0043068~positive regulation of programmed cell death | 5 | 0.047907 |
| GO:0006458~'de novo' protein folding | 2 | 0.04969 |

**Supplementary Table 12. RNA-Seq mapping statistics for replication study**

| **Sample** | **Profile** | **RIN** | **Read length (101bp)** | **Total Read** | **Mapped Read** |
| --- | --- | --- | --- | --- | --- |
| L49 | Schizophrenia | 7.7 | 101 | 99414812 | 88497075 |
| L46 | Schizophrenia | 7.4 | 101 | 108175080 | 99755218 |
| L43 | Schizophrenia | 6.2 | 101 | 94730314 | 86495039 |
| L42 | Schizophrenia | 6.9 | 101 | 98725408 | 90508820 |
| L38 | Schizophrenia | 7.7 | 101 | 103735282 | 93076110 |
| L36 | Schizophrenia | 7.1 | 101 | 92614614 | 83280025 |
| L31 | Schizophrenia | 6.7 | 101 | 101662934 | 93347865 |
| L27 | Schizophrenia | 7.5 | 101 | 119141852 | 104858332 |
| L26 | Schizophrenia | 7 | 101 | 85301092 | 80115220 |
| L23 | Schizophrenia | 6 | 101 | 111360754 | 103662677 |
| L19 | Schizophrenia | 7.1 | 101 | 94645114 | 88667205 |
| L16 | Schizophrenia | 7.1 | 101 | 95436446 | 89740232 |
| L13 | Schizophrenia | 6.9 | 101 | 101965064 | 93294962 |
| L12 | Schizophrenia | 6.2 | 101 | 94906952 | 87246707 |
| L54 | Schizophrenia | 7.5 | 101 | 103086018 | 92961825 |
| L7 | Schizophrenia | 6.8 | 101 | 106555568 | 97464321 |
| L6 | Schizophrenia | 6.2 | 101 | 104086130 | 94832024 |
| L3 | Schizophrenia | 6.9 | 101 | 103954742 | 93527656 |
| L53 | Schizophrenia | 5.8 | 101 | 120009010 | 108370810 |
| L58 | Normal | 7.9 | 101 | 99558004 | 88294108 |
| L45 | Normal | 7.3 | 101 | 94804430 | 85186370 |
| L44 | Normal | 6.4 | 101 | 95698864 | 87881660 |
| L57 | Normal | 7.9 | 101 | 111653742 | 99877946 |
| L37 | Normal | 7.3 | 101 | 104629630 | 93866065 |
| L32 | Normal | 7.1 | 101 | 96657918 | 86033764 |
| L56 | Normal | 7.9 | 101 | 95243046 | 86550559 |
| L29 | Normal | 5.5 | 101 | 101692946 | 94808238 |
| L55 | Normal | 6.1 | 101 | 102802696 | 93368225 |
| L18 | Normal | 7.7 | 101 | 97276802 | 91034715 |
| L15 | Normal | 6.3 | 101 | 92919280 | 86582264 |
| L11 | Normal | 7 | 101 | 95215996 | 85190852 |
| L10 | Normal | 7.3 | 101 | 97365460 | 87324557 |
| L9 | Normal | 7.2 | 101 | 100468836 | 88902014 |
| L5 | Normal | 7.5 | 101 | 98025736 | 89223898 |
| L4 | Normal | 6.5 | 101 | 90769770 | 83121205 |
| L52 | Normal | 7 | 101 | 100954354 | 93276695 |
| L51 | Normal | 7.9 | 101 | 87812690 | 78309769 |
| L50 | Normal | 6 | 101 | 95031666 | 86746200 |
|  |  |  | **Mean** | 99949711.9 | 90928453 |

^1^Mapping to a reference genome (hg19)

**Supplementary Table 13.** Genes differentially expressed between schizophrenia and controls in the choroid plexus in the replication study

| **Gene** | **log FC^1^** | **log CPM^2^** | **P Value** | **FDR** |
| --- | --- | --- | --- | --- |
| *XIST* | 9.227403 | 3.403135 | 1.55E-14 | 2.54E-10 |
| *HMGCS2* | 3.197773 | 3.720924 | 3.68E-09 | 3.02E-05 |
| *WFIKKN2* | 2.385341 | 9.326031 | 1.52E-07 | 0.000834 |
| *ADAMTS17* | 1.572082 | 3.209952 | 3.32E-07 | 0.001361 |
| *RASL10B* | 1.552194 | 3.554337 | 1.14E-06 | 0.003755 |
| *SERPINA3* | 2.186212 | 7.535142 | 1.98E-06 | 0.005413 |
| *SLC2A14* | -3.28767 | -1.33308 | 2.82E-06 | 0.006604 |
| *PEBP4* | 1.166706 | 4.217983 | 6.29E-06 | 0.011859 |
| *CXCL3* | -2.35161 | 1.320776 | 6.53E-06 | 0.011859 |
| *SPON2* | -0.82455 | 3.886455 | 7.63E-06 | 0.011859 |
| *SEMA3G* | -1.71167 | 4.825646 | 7.94E-06 | 0.011859 |
| *GEMIN8* | 0.581731 | 4.553101 | 1.10E-05 | 0.015029 |
| *MYBPH* | 1.713219 | -1.07348 | 1.49E-05 | 0.018855 |
| *FAM131C* | 1.61723 | 2.441921 | 1.96E-05 | 0.022958 |
| *MT1X* | 1.574849 | 5.021611 | 2.83E-05 | 0.029172 |
| *TG* | 1.854611 | 0.698963 | 2.94E-05 | 0.029172 |
| *MT1A* | 1.902079 | 1.716791 | 3.03E-05 | 0.029172 |
| *SULT1E1* | 2.246524 | 3.453127 | 3.20E-05 | 0.029172 |
| *TMEM106C* | 0.436822 | 6.573537 | 3.69E-05 | 0.031882 |
| *KIFC1* | -1.59848 | -0.18613 | 4.36E-05 | 0.033511 |
| *AADACL4* | 1.966971 | -0.12723 | 4.41E-05 | 0.033511 |
| *FAM198B* | -0.72886 | 5.028681 | 5.00E-05 | 0.033511 |
| *ADSSL1* | 1.020954 | 4.665504 | 5.12E-05 | 0.033511 |
| *SPOCD1* | 1.673903 | -0.67384 | 5.27E-05 | 0.033511 |
| *RPS16P5* | -1.71244 | -1.19468 | 5.47E-05 | 0.033511 |
| *GLS* | -0.41238 | 7.334856 | 5.50E-05 | 0.033511 |
| *HBG2* | 2.488107 | 1.512986 | 5.70E-05 | 0.033511 |
| *CISH* | 0.717218 | 2.616684 | 5.71E-05 | 0.033511 |
| *RARRES1* | 1.851676 | 4.185503 | 6.39E-05 | 0.035487 |
| *FUOM* | 0.710765 | 3.929618 | 6.48E-05 | 0.035487 |
| *SYTL5* | -1.08218 | 0.778456 | 6.87E-05 | 0.036396 |
| *PRKX* | 0.872779 | 5.529497 | 7.43E-05 | 0.038151 |
| *GOLT1A* | 0.937179 | -0.07864 | 8.97E-05 | 0.044651 |

logFC, log2 fold change ; logCPM, log2 counts-per-million

**Supplementary Table 14.** Correlation coefficients between co-expression modules and descriptive variables for the replication study

| **Module** | **Schizophrenia** | **Age** | **Sex** | **PMI** | **Brain pH** | **Antipsychotics** | **RIN** |
| --- | --- | --- | --- | --- | --- | --- | --- |
| S_R_M1 | ns | ns | ns | ns | ns | ns | ns |
| S_R_M2 | 0.32 (Padj=0.37) | ns | ns | ns | -0.52 (Padj=0.04) | ns | ns |
| S_R_M3 | ns | ns | ns | ns | ns | ns | ns |
| S_R_M4 | ns | ns | ns | ns | ns | ns | ns |
| S_R_M5 | ns | ns | ns | ns | ns | ns | ns |
| S_R_M6 | ns | ns | ns | ns | ns | ns | ns |
| S_R_M7 | ns | ns | ns | ns | ns | ns | ns |
| S_R_M8 | ns | ns | ns | ns | ns | ns | ns |
| S_R_M9 | 0.58 (Padj<0.001) | ns | 0.98 (Padj<0.001) | ns | ns | ns | ns |
| S_R_M10 | ns | ns | ns | ns | ns | ns | -0.5 (Padj=0.002) |
| S_R_M11 | ns | ns | 0.49 (Padj=0.03) | ns | ns | ns | -0.55 (Padj=0.005) |
| S_R_M12 | ns | ns | ns | ns | ns | ns | ns |
| S_R_M13 | ns | ns | ns | ns | ns | ns | ns |
| S_R_M14 | ns | ns | ns | ns | ns | ns | ns |
| S_R_M15 | ns | ns | -0.5 (Padj=0.02) | ns | ns | ns | 0.55 (Padj=0.005) |
| S_R_M16 | ns | ns | ns | ns | ns | ns | ns |
| S_R_M17 | ns | ns | ns | ns | ns | ns | ns |
| S_R_M18 | ns | ns | ns | ns | ns | ns | ns |
| S_R_M19 | ns | ns | ns | ns | ns | ns | ns |
| S_R_M20 | ns | ns | ns | ns | ns | ns | ns |
| S_R_M21 | ns | ns | ns | ns | ns | ns | ns |
| S_R_M22 | ns | ns | ns | ns | ns | ns | ns |
| S_R_M23 | ns | ns | ns | ns | ns | ns | ns |
| S_R_M24 | ns | ns | ns | ns | ns | ns | ns |

**Supplementary Table 15.**  Biological processes enriched in the genes in the S_R_M2 module

| **Term** | **Count** | **PValue** |
| --- | --- | --- |
| GO:0019221~cytokine-mediated signaling pathway | 10 | 2.03E-07 |
| GO:0042127~regulation of cell proliferation | 28 | 1.55E-06 |
| GO:0051094~positive regulation of developmental process | 16 | 2.10E-06 |
| GO:0008284~positive regulation of cell proliferation | 19 | 4.27E-06 |
| GO:0042325~regulation of phosphorylation | 19 | 2.14E-05 |
| GO:0048545~response to steroid hormone stimulus | 12 | 2.91E-05 |
| GO:0045597~positive regulation of cell differentiation | 13 | 3.04E-05 |
| GO:0051174~regulation of phosphorus metabolic process | 19 | 3.62E-05 |
| GO:0019220~regulation of phosphate metabolic process | 19 | 3.62E-05 |
| GO:0034097~response to cytokine stimulus | 8 | 5.71E-05 |
| GO:0032570~response to progesterone stimulus | 5 | 9.74E-05 |
| GO:0007167~enzyme linked receptor protein signaling pathway | 15 | 9.96E-05 |
| GO:0010033~response to organic substance | 22 | 2.57E-04 |
| GO:0042517~positive regulation of tyrosine phosphorylation of Stat3 protein | 4 | 2.94E-04 |
| GO:0008285~negative regulation of cell proliferation | 14 | 6.06E-04 |
| GO:0009612~response to mechanical stimulus | 6 | 6.55E-04 |
| GO:0001501~skeletal system development | 13 | 6.71E-04 |
| GO:0009611~response to wounding | 17 | 9.89E-04 |
| GO:0042516~regulation of tyrosine phosphorylation of Stat3 protein | 4 | 0.001145 |
| GO:0007259~JAK-STAT cascade | 5 | 0.001375 |
| GO:0001932~regulation of protein amino acid phosphorylation | 9 | 0.001485 |
| GO:0043405~regulation of MAP kinase activity | 8 | 0.001952 |
| GO:0043627~response to estrogen stimulus | 7 | 0.00202 |
| GO:0031349~positive regulation of defense response | 6 | 0.002172 |
| GO:0009725~response to hormone stimulus | 13 | 0.002219 |
| GO:0016477~cell migration | 11 | 0.002468 |
| GO:0006928~cell motion | 15 | 0.002548 |
| GO:0001525~angiogenesis | 8 | 0.002569 |
| GO:0050727~regulation of inflammatory response | 6 | 0.002593 |
| GO:0042531~positive regulation of tyrosine phosphorylation of STAT protein | 4 | 0.002823 |
| GO:0010647~positive regulation of cell communication | 12 | 0.002838 |
| GO:0051270~regulation of cell motion | 9 | 0.002937 |
| GO:0002684~positive regulation of immune system process | 10 | 0.003035 |
| GO:0002645~positive regulation of tolerance induction | 3 | 0.003127 |
| GO:0002643~regulation of tolerance induction | 3 | 0.003127 |
| GO:0032101~regulation of response to external stimulus | 8 | 0.003824 |
| GO:0046427~positive regulation of JAK-STAT cascade | 4 | 0.004032 |
| GO:0032355~response to estradiol stimulus | 5 | 0.004573 |
| GO:0002694~regulation of leukocyte activation | 8 | 0.004839 |
| GO:0009719~response to endogenous stimulus | 13 | 0.004904 |
| GO:0030335~positive regulation of cell migration | 6 | 0.005118 |
| GO:0051674~localization of cell | 11 | 0.005238 |
| GO:0048870~cell motility | 11 | 0.005238 |
| GO:0030334~regulation of cell migration | 8 | 0.00531 |
| GO:0043069~negative regulation of programmed cell death | 12 | 0.005461 |
| GO:0060548~negative regulation of cell death | 12 | 0.005574 |
| GO:0007566~embryo implantation | 4 | 0.00607 |
| GO:0050729~positive regulation of inflammatory response | 4 | 0.00607 |
| GO:0050865~regulation of cell activation | 8 | 0.006422 |
| GO:0006470~protein amino acid dephosphorylation | 7 | 0.006472 |
| GO:0009968~negative regulation of signal transduction | 9 | 0.006603 |
| GO:0042509~regulation of tyrosine phosphorylation of STAT protein | 4 | 0.00666 |
| GO:0007243~protein kinase cascade | 12 | 0.006803 |
| GO:0007169~transmembrane receptor protein tyrosine kinase signaling pathway | 9 | 0.007153 |
| GO:0042327~positive regulation of phosphorylation | 6 | 0.007339 |
| GO:0030036~actin cytoskeleton organization | 9 | 0.007507 |
| GO:0051272~positive regulation of cell motion | 6 | 0.007657 |
| GO:0040017~positive regulation of locomotion | 6 | 0.007657 |
| GO:0006954~inflammatory response | 11 | 0.007719 |
| GO:0006939~smooth muscle contraction | 4 | 0.007938 |
| GO:0030324~lung development | 6 | 0.007985 |
| GO:0010562~positive regulation of phosphorus metabolic process | 6 | 0.008323 |
| GO:0045937~positive regulation of phosphate metabolic process | 6 | 0.008323 |
| GO:0032103~positive regulation of response to external stimulus | 5 | 0.008347 |
| GO:0030323~respiratory tube development | 6 | 0.009027 |
| GO:0002675~positive regulation of acute inflammatory response | 3 | 0.009431 |
| GO:0043407~negative regulation of MAP kinase activity | 4 | 0.010109 |
| GO:0046425~regulation of JAK-STAT cascade | 4 | 0.010109 |
| GO:0040012~regulation of locomotion | 8 | 0.010447 |
| GO:0002696~positive regulation of leukocyte activation | 6 | 0.010557 |
| GO:0051249~regulation of lymphocyte activation | 7 | 0.010687 |
| GO:0030029~actin filament-based process | 9 | 0.010893 |
| GO:0045449~regulation of transcription | 46 | 0.011213 |
| GO:0060541~respiratory system development | 6 | 0.011385 |
| GO:0045859~regulation of protein kinase activity | 11 | 0.011449 |
| GO:0051240~positive regulation of multicellular organismal process | 9 | 0.011497 |
| GO:0050867~positive regulation of cell activation | 6 | 0.012708 |
| GO:0010648~negative regulation of cell communication | 9 | 0.012725 |
| GO:0033135~regulation of peptidyl-serine phosphorylation | 3 | 0.012793 |
| GO:0016311~dephosphorylation | 7 | 0.012813 |
| GO:0048705~skeletal system morphogenesis | 6 | 0.013171 |
| GO:0043066~negative regulation of apoptosis | 11 | 0.013517 |
| GO:0001944~vasculature development | 9 | 0.013584 |
| GO:0043549~regulation of kinase activity | 11 | 0.014265 |
| GO:0006916~anti-apoptosis | 8 | 0.015087 |
| GO:0051384~response to glucocorticoid stimulus | 5 | 0.016392 |
| GO:0048514~blood vessel morphogenesis | 8 | 0.016804 |
| GO:0009628~response to abiotic stimulus | 11 | 0.017276 |
| GO:0014070~response to organic cyclic substance | 6 | 0.017861 |
| GO:0051338~regulation of transferase activity | 11 | 0.01848 |
| GO:0009749~response to glucose stimulus | 4 | 0.018519 |
| GO:0050670~regulation of lymphocyte proliferation | 5 | 0.020136 |
| GO:0009266~response to temperature stimulus | 5 | 0.020136 |
| GO:0002683~negative regulation of immune system process | 5 | 0.020136 |
| GO:0035295~tube development | 8 | 0.020623 |
| GO:0050731~positive regulation of peptidyl-tyrosine phosphorylation | 4 | 0.020784 |
| GO:0009746~response to hexose stimulus | 4 | 0.020784 |
| GO:0034284~response to monosaccharide stimulus | 4 | 0.020784 |
| GO:0032944~regulation of mononuclear cell proliferation | 5 | 0.020942 |
| GO:0070663~regulation of leukocyte proliferation | 5 | 0.020942 |
| GO:0031960~response to corticosteroid stimulus | 5 | 0.021769 |
| GO:0002237~response to molecule of bacterial origin | 5 | 0.022615 |
| GO:0048008~platelet-derived growth factor receptor signaling pathway | 3 | 0.023079 |
| GO:0001934~positive regulation of protein amino acid phosphorylation | 5 | 0.025273 |
| GO:0001817~regulation of cytokine production | 7 | 0.026065 |
| GO:0010941~regulation of cell death | 18 | 0.026167 |
| GO:0040008~regulation of growth | 10 | 0.0275 |
| GO:0002673~regulation of acute inflammatory response | 3 | 0.027886 |
| GO:0048584~positive regulation of response to stimulus | 8 | 0.028893 |
| GO:0031399~regulation of protein modification process | 9 | 0.031966 |
| GO:0009967~positive regulation of signal transduction | 9 | 0.031966 |
| GO:0042060~wound healing | 7 | 0.032701 |
| GO:0009408~response to heat | 4 | 0.032771 |
| GO:0006776~vitamin A metabolic process | 3 | 0.033056 |
| GO:0051251~positive regulation of lymphocyte activation | 5 | 0.033248 |
| GO:0001568~blood vessel development | 8 | 0.03414 |
| GO:0002526~acute inflammatory response | 5 | 0.034337 |
| GO:0006796~phosphate metabolic process | 20 | 0.03477 |
| GO:0006793~phosphorus metabolic process | 20 | 0.03477 |
| GO:0070102~interleukin-6-mediated signaling pathway | 2 | 0.037014 |
| GO:0032224~positive regulation of synaptic transmission, cholinergic | 2 | 0.037014 |
| GO:0045669~positive regulation of osteoblast differentiation | 3 | 0.038568 |
| GO:0009743~response to carbohydrate stimulus | 4 | 0.039034 |
| GO:0010604~positive regulation of macromolecule metabolic process | 18 | 0.039453 |
| GO:0007242~intracellular signaling cascade | 24 | 0.040041 |
| GO:0051329~interphase of mitotic cell cycle | 5 | 0.040091 |
| GO:0010817~regulation of hormone levels | 6 | 0.040984 |
| GO:0031100~organ regeneration | 3 | 0.041446 |
| GO:0051325~interphase | 5 | 0.043792 |
| GO:0010035~response to inorganic substance | 7 | 0.043821 |
| GO:0045765~regulation of angiogenesis | 4 | 0.044101 |
| GO:0006952~defense response | 14 | 0.044636 |
| GO:0045471~response to ethanol | 4 | 0.045859 |
| GO:0003006~reproductive developmental process | 8 | 0.0464 |
| GO:0043067~regulation of programmed cell death | 17 | 0.04733 |
| GO:0048754~branching morphogenesis of a tube | 4 | 0.047652 |
| GO:0007565~female pregnancy | 5 | 0.049015 |
| GO:0070498~interleukin-1-mediated signaling pathway | 2 | 0.049047 |
| GO:0048861~leukemia inhibitory factor signaling pathway | 2 | 0.049047 |
| GO:0032222~regulation of synaptic transmission, cholinergic | 2 | 0.049047 |
| GO:0002548~monocyte chemotaxis | 2 | 0.049047 |

**Supplementary Table 16.** Module preservation statistics for schizophrenia and controls for the replication study

| CTL to schizophrenia | | schizophrenia to CTL | |
| --- | --- | --- | --- |
| Module | Z _summary_ | Module | Z _summary_ |
| C_R_only_M1 | 28 | S_R_only_M1 | **5.8** |
| C_R_only_M2 | 37 | S_R_only_M2 | 43 |
| C_R_only_M3 | 48 | S_R_only_M3 | 25 |
| C_R_only_M4 | 11 | S_R_only_M4 | 15 |
| C_R_only_M5 | 57 | S_R_only_M5 | 13 |
| C_R_only_M6 | 33 | S_R_only_M6 | 11 |
| C_R_only_M7 | 14 | S_R_only_M7 | **7.8** |
| C_R_only_M8 | 10 | S_R_only_M8 | 39 |
| C_R_only_M9 | 11 | S_R_only_M9 | 18 |
| C_R_only_M10 | **4.9** | S_R_only_M10 | 13 |
| C_R_only_M11 | 33 | S_R_only_M11 | 16 |
| C_R_only_M12 | 13 | S_R_only_M12 | **0.47** |
| C_R_only_M13 | 29 | S_R_only_M13 | 15 |
| C_R_only_M14 | 32 | S_R_only_M14 | 28 |
| C_R_only_M15 | 32 | S_R_only_M15 | 14 |
| C_R_only_M16 | 20 | S_R_only_M16 | 17 |
| C_R_only_M17 | 13 | S_R_only_M17 | 23 |
| C_R_only_M18 | 45 | S_R_only_M18 | **-0.29** |
| C_R_only_M19 | 34 | S_R_only_M19 | 13 |
|  |  | S_R_only_M20 | **8.8** |
|  |  | S_R_only_M21 | 21 |
|  |  | S_R_only_M22 | 44 |
|  |  | S_R_only_M23 | 28 |

**Supplementary Table 17.** Correlation coefficients between co-expression modules and descriptive variables.

| **Module** | **Age** | **PMI** | **Brain pH** | **RIN** |
| --- | --- | --- | --- | --- |
| C_R_only1_M1 | ns | ns | ns | ns |
| C_R_only1_M2 | ns | ns | ns | ns |
| C_R_only1_M3 | ns | ns | ns | ns |
| C_R_only1_M4 | ns | ns | ns | -0.63 (Padj=0.04) |
| C_R_only1_M5 | ns | ns | ns | ns |
| C_R_only1_M6 | ns | ns | ns | ns |
| C_R_only1_M7 | ns | ns | ns | ns |
| C_R_only1_M8 | ns | ns | ns | ns |
| C_R_only1_M9 | ns | ns | ns | ns |
| C_R_only1_M10 | ns | 0.8 (Padj=0.015) | ns | ns |
| C_R_only1_M11 | ns | ns | ns | ns |
| C_R_only1_M12 | ns | ns | ns | ns |
| C_R_only1_M13 | ns | ns | ns | ns |
| C_R_only1_M14 | ns | ns | ns | ns |
| C_R_only1_M15 | ns | ns | ns | ns |
| C_R_only1_M16 | ns | ns | ns | ns |
| C_R_only1_M17 | ns | ns | ns | ns |
| C_R_only1_M18 | ns | ns | ns | ns |
| C_R_only1_M19 | ns | ns | ns | ns |

**Supplementary Table 18.**  Biological processes enriched in the genes in the C_R_only_M10 module

| **Term** | **Count** | **PValue** |
| --- | --- | --- |
| GO:0007585~respiratory gaseous exchange | 4 | 1.37E-05 |
| GO:0030334~regulation of cell migration | 4 | 0.001361 |
| GO:0040012~regulation of locomotion | 4 | 0.001963 |
| GO:0051270~regulation of cell motion | 4 | 0.001992 |
| GO:0044057~regulation of system process | 4 | 0.007467 |
| GO:0042127~regulation of cell proliferation | 5 | 0.018078 |
| GO:0003012~muscle system process | 3 | 0.020588 |
| GO:0002685~regulation of leukocyte migration | 2 | 0.026296 |
| GO:0051046~regulation of secretion | 3 | 0.029013 |
| GO:0051240~positive regulation of multicellular organismal process | 3 | 0.041001 |
| GO:0060341~regulation of cellular localization | 3 | 0.042228 |
| GO:0046887~positive regulation of hormone secretion | 2 | 0.046861 |
| GO:0019722~calcium-mediated signaling | 2 | 0.048133 |
| GO:0001818~negative regulation of cytokine production | 2 | 0.049403 |

**Supplementary Table 19.** Correlation coefficients between co-expression modules and descriptive variables.

| **Module** | **Age** | **Sex** | **PMI** | **Brain pH** | **Antipsychotics** | **RIN** |
| --- | --- | --- | --- | --- | --- | --- |
| S_R_only_M1 | ns | ns | ns | ns | ns | ns |
| S_R_only_M2 | ns | ns | 0.77 (Padj=0.01) | ns | ns | ns |
| S_R_only_M3 | ns | ns | ns | ns | ns | ns |
| S_R_only_M4 | ns | ns | ns | ns | ns | ns |
| S_R_only_M5 | ns | ns | ns | ns | ns | ns |
| S_R_only_M6 | ns | -0.61 (Padj=0.05) | ns | ns | ns | 0.63 (Padj=0.04) |
| S_R_only_M7 | ns | ns | ns | ns | ns | ns |
| S_R_only_M8 | ns | ns | ns | ns | ns | -0.63 (Padj=0.04) |
| S_R_only_M9 | ns | ns | ns | ns | ns | ns |
| S_R_only_M10 | ns | ns | ns | ns | ns | ns |
| S_R_only_M11 | ns | ns | ns | ns | ns | -0.72 (Padj=0.005) |
| S_R_only_M12 | ns | ns | ns | ns | ns | ns |
| S_R_only_M13 | ns | ns | ns | ns | ns | -0.72 (Padj=0.006) |
| S_R_only_M14 | ns | ns | ns | ns | ns | ns |
| S_R_only_M15 | ns | ns | ns | ns | ns | ns |
| S_R_only_M16 | ns | ns | ns | ns | ns | ns |
| S_R_only_M17 | ns | ns | ns | ns | ns | ns |
| S_R_only_M18 | ns | ns | ns | ns | ns | ns |
| S_R_only_M19 | ns | ns | ns | ns | ns | ns |
| S_R_only_M20 | ns | ns | ns | ns | ns | ns |
| S_R_only_M21 | ns | ns | ns | ns | ns | ns |
| S_R_only_M22 | ns | ns | ns | ns | ns | ns |
| S_R_only_M23 | ns | ns | ns | ns | ns | ns |

**Supplementary Table 20.**  Biological processes enriched in the genes in the S_R_only_M1 module

| **Term** | **Count** | **PValue** |
| --- | --- | --- |
| GO:0006955~immune response | 22 | 2.78E-12 |
| GO:0009615~response to virus | 10 | 1.95E-09 |
| GO:0019882~antigen processing and presentation | 6 | 4.42E-05 |
| GO:0048002~antigen processing and presentation of peptide antigen | 4 | 3.04E-04 |
| GO:0006952~defense response | 10 | 0.002301 |
| GO:0002474~antigen processing and presentation of peptide antigen via MHC class I | 3 | 0.002862 |
| GO:0032020~ISG15-protein conjugation | 2 | 0.023435 |
| GO:0045580~regulation of T cell differentiation | 3 | 0.02421 |
| GO:0045619~regulation of lymphocyte differentiation | 3 | 0.03577 |
| GO:0030325~adrenal gland development | 2 | 0.04633 |

**Supplementary Table 21.**  Biological processes enriched in the genes in the S_R_only_M12 module

| **Term** | **Count** | **PValue** |
| --- | --- | --- |
| GO:0001836~release of cytochrome c from mitochondria | 3 | 6.74E-04 |
| GO:0008637~apoptotic mitochondrial changes | 3 | 0.001475 |
| GO:0044057~regulation of system process | 5 | 0.002311 |
| GO:0043086~negative regulation of catalytic activity | 4 | 0.013999 |
| GO:0044092~negative regulation of molecular function | 4 | 0.022948 |
| GO:0007005~mitochondrion organization | 3 | 0.026589 |
| GO:0008285~negative regulation of cell proliferation | 4 | 0.028064 |
| GO:0006936~muscle contraction | 3 | 0.032162 |
| GO:0003012~muscle system process | 3 | 0.038155 |
| GO:0008015~blood circulation | 3 | 0.045865 |
| GO:0003013~circulatory system process | 3 | 0.045865 |
| GO:0051289~protein homotetramerization | 2 | 0.046997 |
| GO:0045428~regulation of nitric oxide biosynthetic process | 2 | 0.048762 |

**Supplementary Table 22.**  Biological processes enriched in the genes in the S_R_only_M18 module

| **Term** | **Count** | **PValue** |
| --- | --- | --- |
| GO:0007584~response to nutrient | 4 | 0.045585 |
| GO:0048385~regulation of retinoic acid receptor signaling pathway | 2 | 0.050091 |

**Supplementary Table 23.**  Biological processes enriched in the genes in the S_R_only_M7 module

| **Term** | **Count** | **PValue** |
| --- | --- | --- |
| GO:0006656~phosphatidylcholine biosynthetic process | 2 | 0.021525 |
| GO:0006936~muscle contraction | 3 | 0.023183 |
| GO:0003012~muscle system process | 3 | 0.027582 |
| GO:0046470~phosphatidylcholine metabolic process | 2 | 0.032121 |
| GO:0055074~calcium ion homeostasis | 3 | 0.033928 |
| GO:0055065~metal ion homeostasis | 3 | 0.039729 |
| GO:0010604~positive regulation of macromolecule metabolic process | 5 | 0.040374 |
| GO:0048878~chemical homeostasis | 4 | 0.043196 |

**Supplementary Table 24.**  Biological processes enriched in the genes in the S_R_only_M20 module

| **Term** | **Count** | **PValue** |
| --- | --- | --- |
| GO:0007218~neuropeptide signaling pathway | 3 | 0.022742 |
| GO:0006468~protein amino acid phosphorylation | 6 | 0.024407 |
| GO:0007018~microtubule-based movement | 3 | 0.032609 |
| GO:0016310~phosphorylation | 6 | 0.047872 |

**Supplementary Table 25.** Correlation coefficients between co-expression modules of the combined data and descriptive variables

| **Module** | **Schizophrenia** | **Age** | **Sex** | **PMI** | **Brain pH** | **Antipsychotics** | **RIN** |
| --- | --- | --- | --- | --- | --- | --- | --- |
| S_Co_M1 | ns | ns | ns | ns | ns | ns | ns |
| S_Co_M2 | ns | ns | ns | ns | ns | ns | ns |
| S_Co_M3 | ns | ns | ns | ns | ns | ns | ns |
| S_Co_M4 | ns | ns | ns | ns | ns | ns | ns |
| S_Co_M5 | ns | ns | ns | ns | ns | ns | ns |
| S_Co_M6 | ns | ns | ns | ns | -0.34 (Padj=0.02) | ns | ns |
| S_Co_M7 | ns | ns | -0.98 (Padj<0.001) | ns | ns | ns | ns |
| S_Co_M8 | ns | ns | ns | ns | ns | ns | ns |
| S_Co_M9 | ns | ns | ns | ns | ns | ns | ns |
| S_Co_M10 | ns | ns | ns | ns | ns | ns | ns |
| S_Co_M11 | ns | ns | ns | ns | ns | ns | ns |
| S_Co_M12 | ns | ns | ns | ns | ns | ns | ns |
| S_Co_M13 | ns | ns | ns | ns | ns | ns | ns |
| S_Co_M14 | ns | ns | ns | ns | ns | ns | ns |
| S_Co_M15 | ns | ns | ns | ns | ns | ns | ns |
| S_Co_M16 | 0.311 (Padj=0.03) | ns | ns | ns | -0.31 (Padj=0.05) | ns | ns |
| S_Co_M17 | ns | ns | ns | ns | ns | ns | ns |
| S_Co_M18 | ns | ns | ns | ns | ns | ns | ns |
| S_Co_M19 | ns | ns | ns | ns | ns | ns | ns |
| S_Co_M20 | ns | ns | ns | ns | ns | ns | ns |
| S_Co_M21 | ns | ns | ns | ns | ns | ns | -0.33 (Padj=0.02) |
| S_Co_M22 | ns | ns | ns | ns | ns | ns | 0.355 (Padj=0.01) |
| S_Co_M23 | ns | ns | ns | ns | ns | ns | ns |

**Supplementary Table 26.**  Biological processes significantly enriched in the genes in the S_Co_M16 module

| **Term** | **Count** | **PValue** |
| --- | --- | --- |
| GO:0006952~defense response | 47 | 1.13E-14 |
| GO:0006954~inflammatory response | 34 | 1.96E-14 |
| GO:0009611~response to wounding | 43 | 2.60E-14 |
| GO:0001817~regulation of cytokine production | 20 | 4.88E-09 |
| GO:0006955~immune response | 40 | 6.05E-09 |
| GO:0034097~response to cytokine stimulus | 13 | 5.98E-08 |
| GO:0031349~positive regulation of defense response | 12 | 2.34E-07 |
| GO:0002684~positive regulation of immune system process | 20 | 3.99E-07 |
| GO:0002237~response to molecule of bacterial origin | 12 | 1.27E-06 |
| GO:0010033~response to organic substance | 36 | 1.49E-06 |
| GO:0042127~regulation of cell proliferation | 38 | 1.56E-06 |
| GO:0048584~positive regulation of response to stimulus | 19 | 1.58E-06 |
| GO:0009617~response to bacterium | 17 | 2.05E-06 |
| GO:0032496~response to lipopolysaccharide | 11 | 3.38E-06 |
| GO:0001819~positive regulation of cytokine production | 11 | 1.40E-05 |
| GO:0045087~innate immune response | 13 | 2.45E-05 |
| GO:0045089~positive regulation of innate immune response | 8 | 3.61E-05 |
| GO:0051240~positive regulation of multicellular organismal process | 17 | 3.96E-05 |
| GO:0050778~positive regulation of immune response | 13 | 4.01E-05 |
| GO:0032570~response to progesterone stimulus | 6 | 4.04E-05 |
| GO:0045088~regulation of innate immune response | 8 | 1.04E-04 |
| GO:0008284~positive regulation of cell proliferation | 22 | 1.13E-04 |
| GO:0050727~regulation of inflammatory response | 9 | 1.50E-04 |
| GO:0051094~positive regulation of developmental process | 17 | 1.84E-04 |
| GO:0002221~pattern recognition receptor signaling pathway | 5 | 3.23E-04 |
| GO:0070555~response to interleukin-1 | 5 | 3.23E-04 |
| GO:0032680~regulation of tumor necrosis factor production | 6 | 3.69E-04 |
| GO:0032101~regulation of response to external stimulus | 12 | 4.06E-04 |
| GO:0002758~innate immune response-activating signal transduction | 5 | 5.09E-04 |
| GO:0002218~activation of innate immune response | 5 | 5.09E-04 |
| GO:0002449~lymphocyte mediated immunity | 8 | 5.34E-04 |
| GO:0019221~cytokine-mediated signaling pathway | 8 | 5.34E-04 |
| GO:0070482~response to oxygen levels | 11 | 6.10E-04 |
| GO:0016064~immunoglobulin mediated immune response | 7 | 7.61E-04 |
| GO:0019724~B cell mediated immunity | 7 | 9.25E-04 |
| GO:0002250~adaptive immune response | 8 | 9.50E-04 |
| GO:0002460~adaptive immune response based on somatic recombination of immune receptors built from immunoglobulin superfamily domains | 8 | 9.50E-04 |
| GO:0001818~negative regulation of cytokine production | 6 | 9.70E-04 |
| GO:0008285~negative regulation of cell proliferation | 18 | 0.001 |
| GO:0007265~Ras protein signal transduction | 9 | 0.001 |
| GO:0042592~homeostatic process | 29 | 0.001 |
| GO:0002683~negative regulation of immune system process | 8 | 0.001 |
| GO:0007243~protein kinase cascade | 18 | 0.002 |
| GO:0001666~response to hypoxia | 10 | 0.002 |
| GO:0009615~response to virus | 9 | 0.002 |
| GO:0010648~negative regulation of cell communication | 14 | 0.002 |
| GO:0045766~positive regulation of angiogenesis | 5 | 0.002 |
| GO:0002443~leukocyte mediated immunity | 8 | 0.002 |
| GO:0042060~wound healing | 12 | 0.002 |
| GO:0009968~negative regulation of signal transduction | 13 | 0.002 |
| GO:0042981~regulation of apoptosis | 30 | 0.002 |
| GO:0042742~defense response to bacterium | 9 | 0.002 |
| GO:0010740~positive regulation of protein kinase cascade | 11 | 0.002 |
| GO:0043067~regulation of programmed cell death | 30 | 0.002 |
| GO:0001816~cytokine production | 6 | 0.002 |
| GO:0010941~regulation of cell death | 30 | 0.002 |
| GO:0030005~cellular di-, tri-valent inorganic cation homeostasis | 13 | 0.002 |
| GO:0045597~positive regulation of cell differentiation | 13 | 0.003 |
| GO:0051480~cytosolic calcium ion homeostasis | 9 | 0.003 |
| GO:0032649~regulation of interferon-gamma production | 5 | 0.003 |
| GO:0002253~activation of immune response | 8 | 0.003 |
| GO:0032760~positive regulation of tumor necrosis factor production | 4 | 0.003 |
| GO:0014070~response to organic cyclic substance | 9 | 0.003 |
| GO:0015718~monocarboxylic acid transport | 6 | 0.003 |
| GO:0055066~di-, tri-valent inorganic cation homeostasis | 13 | 0.004 |
| GO:0002526~acute inflammatory response | 8 | 0.004 |
| GO:0032695~negative regulation of interleukin-12 production | 3 | 0.004 |
| GO:0006006~glucose metabolic process | 10 | 0.004 |
| GO:0048585~negative regulation of response to stimulus | 8 | 0.004 |
| GO:0032655~regulation of interleukin-12 production | 4 | 0.005 |
| GO:0019229~regulation of vasoconstriction | 5 | 0.005 |
| GO:0060341~regulation of cellular localization | 13 | 0.005 |
| GO:0051174~regulation of phosphorus metabolic process | 20 | 0.005 |
| GO:0019220~regulation of phosphate metabolic process | 20 | 0.005 |
| GO:0007242~intracellular signaling cascade | 40 | 0.005 |
| GO:0010627~regulation of protein kinase cascade | 13 | 0.005 |
| GO:0009595~detection of biotic stimulus | 4 | 0.005 |
| GO:0002819~regulation of adaptive immune response | 6 | 0.005 |
| GO:0001501~skeletal system development | 15 | 0.006 |
| GO:0002755~MyD88-dependent toll-like receptor signaling pathway | 3 | 0.006 |
| GO:0030003~cellular cation homeostasis | 13 | 0.006 |
| GO:0002696~positive regulation of leukocyte activation | 8 | 0.006 |
| GO:0048545~response to steroid hormone stimulus | 11 | 0.006 |
| GO:0002252~immune effector process | 9 | 0.006 |
| GO:0030036~actin cytoskeleton organization | 12 | 0.007 |
| GO:0050670~regulation of lymphocyte proliferation | 7 | 0.007 |
| GO:0002694~regulation of leukocyte activation | 10 | 0.007 |
| GO:0042325~regulation of phosphorylation | 19 | 0.007 |
| GO:0007565~female pregnancy | 8 | 0.007 |
| GO:0007204~elevation of cytosolic calcium ion concentration | 8 | 0.007 |
| GO:0070663~regulation of leukocyte proliferation | 7 | 0.007 |
| GO:0032944~regulation of mononuclear cell proliferation | 7 | 0.007 |
| GO:0050830~defense response to Gram-positive bacterium | 4 | 0.007 |
| GO:0005976~polysaccharide metabolic process | 8 | 0.007 |
| GO:0050867~positive regulation of cell activation | 8 | 0.007 |
| GO:0043405~regulation of MAP kinase activity | 9 | 0.008 |
| GO:0048878~chemical homeostasis | 20 | 0.009 |
| GO:0006953~acute-phase response | 5 | 0.009 |
| GO:0051052~regulation of DNA metabolic process | 8 | 0.009 |
| GO:0022610~biological adhesion | 25 | 0.009 |
| GO:0007155~cell adhesion | 25 | 0.009 |
| GO:0051092~positive regulation of NF-kappaB transcription factor activity | 5 | 0.009 |
| GO:0050865~regulation of cell activation | 10 | 0.010 |
| GO:0032103~positive regulation of response to external stimulus | 6 | 0.010 |
| GO:0040008~regulation of growth | 15 | 0.010 |
| GO:0007167~enzyme linked receptor protein signaling pathway | 15 | 0.010 |
| GO:0046942~carboxylic acid transport | 9 | 0.010 |
| GO:0030029~actin filament-based process | 12 | 0.010 |
| GO:0051893~regulation of focal adhesion formation | 3 | 0.011 |
| GO:0015849~organic acid transport | 9 | 0.011 |
| GO:0051249~regulation of lymphocyte activation | 9 | 0.011 |
| GO:0042531~positive regulation of tyrosine phosphorylation of STAT protein | 4 | 0.011 |
| GO:0032652~regulation of interleukin-1 production | 4 | 0.011 |
| GO:0016045~detection of bacterium | 3 | 0.013 |
| GO:0009894~regulation of catabolic process | 7 | 0.013 |
| GO:0010810~regulation of cell-substrate adhesion | 5 | 0.014 |
| GO:0051251~positive regulation of lymphocyte activation | 7 | 0.014 |
| GO:0055080~cation homeostasis | 13 | 0.014 |
| GO:0001775~cell activation | 13 | 0.015 |
| GO:0009991~response to extracellular stimulus | 11 | 0.015 |
| GO:0051272~positive regulation of cell motion | 7 | 0.015 |
| GO:0043065~positive regulation of apoptosis | 17 | 0.015 |
| GO:0050731~positive regulation of peptidyl-tyrosine phosphorylation | 5 | 0.015 |
| GO:0046427~positive regulation of JAK-STAT cascade | 4 | 0.015 |
| GO:0043068~positive regulation of programmed cell death | 17 | 0.016 |
| GO:0010942~positive regulation of cell death | 17 | 0.016 |
| GO:0070227~lymphocyte apoptosis | 3 | 0.017 |
| GO:0001953~negative regulation of cell-matrix adhesion | 3 | 0.017 |
| GO:0019318~hexose metabolic process | 10 | 0.017 |
| GO:0001952~regulation of cell-matrix adhesion | 4 | 0.017 |
| GO:0010647~positive regulation of cell communication | 14 | 0.017 |
| GO:0009581~detection of external stimulus | 6 | 0.017 |
| GO:0009967~positive regulation of signal transduction | 13 | 0.018 |
| GO:0050817~coagulation | 7 | 0.018 |
| GO:0007596~blood coagulation | 7 | 0.018 |
| GO:0009725~response to hormone stimulus | 15 | 0.018 |
| GO:0009719~response to endogenous stimulus | 16 | 0.019 |
| GO:0006875~cellular metal ion homeostasis | 10 | 0.019 |
| GO:0051241~negative regulation of multicellular organismal process | 9 | 0.019 |
| GO:0046636~negative regulation of alpha-beta T cell activation | 3 | 0.020 |
| GO:0032729~positive regulation of interferon-gamma production | 3 | 0.020 |
| GO:0010812~negative regulation of cell-substrate adhesion | 3 | 0.020 |
| GO:0002757~immune response-activating signal transduction | 5 | 0.021 |
| GO:0030278~regulation of ossification | 6 | 0.021 |
| GO:0016051~carbohydrate biosynthetic process | 7 | 0.022 |
| GO:0007264~small GTPase mediated signal transduction | 13 | 0.022 |
| GO:0050729~positive regulation of inflammatory response | 4 | 0.023 |
| GO:0007599~hemostasis | 7 | 0.023 |
| GO:0032355~response to estradiol stimulus | 5 | 0.024 |
| GO:0051250~negative regulation of lymphocyte activation | 5 | 0.024 |
| GO:0055065~metal ion homeostasis | 10 | 0.024 |
| GO:0045786~negative regulation of cell cycle | 6 | 0.025 |
| GO:0042509~regulation of tyrosine phosphorylation of STAT protein | 4 | 0.025 |
| GO:0002821~positive regulation of adaptive immune response | 4 | 0.025 |
| GO:0002455~humoral immune response mediated by circulating immunoglobulin | 4 | 0.025 |
| GO:0050878~regulation of body fluid levels | 8 | 0.025 |
| GO:0001932~regulation of protein amino acid phosphorylation | 9 | 0.025 |
| GO:0002822~regulation of adaptive immune response based on somatic recombination of immune receptors built from immunoglobulin superfamily domains | 5 | 0.025 |
| GO:0050671~positive regulation of lymphocyte proliferation | 5 | 0.025 |
| GO:0044087~regulation of cellular component biogenesis | 8 | 0.026 |
| GO:0070665~positive regulation of leukocyte proliferation | 5 | 0.027 |
| GO:0009612~response to mechanical stimulus | 5 | 0.027 |
| GO:0002764~immune response-regulating signal transduction | 5 | 0.027 |
| GO:0032946~positive regulation of mononuclear cell proliferation | 5 | 0.027 |
| GO:0010595~positive regulation of endothelial cell migration | 3 | 0.028 |
| GO:0002695~negative regulation of leukocyte activation | 5 | 0.028 |
| GO:0006869~lipid transport | 8 | 0.029 |
| GO:0051223~regulation of protein transport | 7 | 0.029 |
| GO:0043066~negative regulation of apoptosis | 14 | 0.029 |
| GO:0002224~toll-like receptor signaling pathway | 3 | 0.032 |
| GO:0043069~negative regulation of programmed cell death | 14 | 0.032 |
| GO:0050863~regulation of T cell activation | 7 | 0.032 |
| GO:0042493~response to drug | 10 | 0.033 |
| GO:0060548~negative regulation of cell death | 14 | 0.033 |
| GO:0008283~cell proliferation | 16 | 0.033 |
| GO:0006928~cell motion | 17 | 0.033 |
| GO:0045785~positive regulation of cell adhesion | 5 | 0.034 |
| GO:0051091~positive regulation of transcription factor activity | 5 | 0.034 |
| GO:0006874~cellular calcium ion homeostasis | 9 | 0.034 |
| GO:0005977~glycogen metabolic process | 4 | 0.034 |
| GO:0030335~positive regulation of cell migration | 6 | 0.035 |
| GO:0032956~regulation of actin cytoskeleton organization | 6 | 0.035 |
| GO:0050866~negative regulation of cell activation | 5 | 0.035 |
| GO:0046425~regulation of JAK-STAT cascade | 4 | 0.036 |
| GO:0031348~negative regulation of defense response | 4 | 0.036 |
| GO:0032675~regulation of interleukin-6 production | 4 | 0.036 |
| GO:0002263~cell activation during immune response | 4 | 0.036 |
| GO:0006073~cellular glucan metabolic process | 4 | 0.036 |
| GO:0044042~glucan metabolic process | 4 | 0.036 |
| GO:0002366~leukocyte activation during immune response | 4 | 0.036 |
| GO:0042116~macrophage activation | 3 | 0.036 |
| GO:0051098~regulation of binding | 8 | 0.037 |
| GO:0051101~regulation of DNA binding | 7 | 0.037 |
| GO:0070201~regulation of establishment of protein localization | 7 | 0.037 |
| GO:0005996~monosaccharide metabolic process | 10 | 0.038 |
| GO:0043434~response to peptide hormone stimulus | 8 | 0.038 |
| GO:0055074~calcium ion homeostasis | 9 | 0.038 |
| GO:0045765~regulation of angiogenesis | 5 | 0.039 |
| GO:0032970~regulation of actin filament-based process | 6 | 0.039 |
| GO:0031328~positive regulation of cellular biosynthetic process | 22 | 0.040 |
| GO:0034122~negative regulation of toll-like receptor signaling pathway | 2 | 0.040 |
| GO:0002577~regulation of antigen processing and presentation | 2 | 0.040 |
| GO:0002604~regulation of dendritic cell antigen processing and presentation | 2 | 0.040 |
| GO:0043471~regulation of cellular carbohydrate catabolic process | 3 | 0.041 |
| GO:0030104~water homeostasis | 3 | 0.041 |
| GO:0043470~regulation of carbohydrate catabolic process | 3 | 0.041 |
| GO:0010876~lipid localization | 8 | 0.041 |
| GO:0006873~cellular ion homeostasis | 14 | 0.043 |
| GO:0010605~negative regulation of macromolecule metabolic process | 23 | 0.044 |
| GO:0051053~negative regulation of DNA metabolic process | 4 | 0.044 |
| GO:0007166~cell surface receptor linked signal transduction | 49 | 0.045 |
| GO:0009891~positive regulation of biosynthetic process | 22 | 0.046 |
| GO:0032732~positive regulation of interleukin-1 production | 3 | 0.046 |
| GO:0042177~negative regulation of protein catabolic process | 3 | 0.046 |
| GO:0055082~cellular chemical homeostasis | 14 | 0.047 |
| GO:0042327~positive regulation of phosphorylation | 6 | 0.048 |
| GO:0043123~positive regulation of I-kappaB kinase/NF-kappaB cascade | 6 | 0.048 |
| GO:0031667~response to nutrient levels | 9 | 0.048 |
| GO:0040017~positive regulation of locomotion | 6 | 0.049 |
| GO:0031589~cell-substrate adhesion | 6 | 0.049 |
| GO:0050730~regulation of peptidyl-tyrosine phosphorylation | 5 | 0.049 |

**Supplementary Table 27.**  Top 20 Biological processes highly enriched in the top 5000 probes that were selected from data of mice 3 hrs post LPS treatment and controls

| **GO Term** | **Description** | **P-value** | **FDR q-value** | **Enrichment** |
| --- | --- | --- | --- | --- |
| GO:0002376 | immune system process | 3.40E-39 | 3.49E-35 | 3.43 |
| GO:0006952 | defense response | 1.11E-38 | 5.70E-35 | 4.18 |
| GO:0006955 | immune response | 3.23E-34 | 1.11E-30 | 4.29 |
| GO:0034097 | response to cytokine | 6.00E-34 | 1.54E-30 | 7.76 |
| GO:0050896 | response to stimulus | 6.25E-30 | 1.28E-26 | 1.98 |
| GO:0043207 | response to external biotic stimulus | 6.15E-29 | 1.05E-25 | 4.41 |
| GO:0009607 | response to biotic stimulus | 1.14E-27 | 1.66E-24 | 4.25 |
| GO:0006950 | response to stress | 2.45E-25 | 3.14E-22 | 2.31 |
| GO:0009605 | response to external stimulus | 3.59E-24 | 4.09E-21 | 3.4 |
| GO:0010033 | response to organic substance | 1.16E-23 | 1.19E-20 | 3.21 |
| GO:0042221 | response to chemical | 1.75E-22 | 1.63E-19 | 2.07 |
| GO:0051707 | response to other organism | 9.69E-22 | 8.28E-19 | 4.71 |
| GO:0002684 | positive regulation of immune system process | 2.94E-21 | 2.32E-18 | 2.79 |
| GO:0006954 | inflammatory response | 3.95E-21 | 2.89E-18 | 9.09 |
| GO:0071345 | cellular response to cytokine stimulus | 1.51E-20 | 1.03E-17 | 7.72 |
| GO:0002252 | immune effector process | 2.65E-20 | 1.70E-17 | 4.09 |
| GO:0031347 | regulation of defense response | 9.97E-20 | 6.01E-17 | 3.89 |
| GO:0048583 | regulation of response to stimulus | 2.20E-19 | 1.26E-16 | 1.92 |
| GO:0009615 | response to virus | 3.07E-19 | 1.65E-16 | 6.53 |
| GO:0002682 | regulation of immune system process | 4.41E-19 | 2.26E-16 | 2.59 |

Enrichment is defined as (the number of genes in the intersection/the number of the target set)/ (the total number of genes associated with a specific GO term/ the total number of genes)

**Supplementary Table 28.**  Biological processes enriched in the genes in the LPS_3h_M2 module

| **Term** | **Count** | **PValue** |
| --- | --- | --- |
| GO:0006955~immune response | 67 | 6.84E-18 |
| GO:0009611~response to wounding | 51 | 3.59E-14 |
| GO:0006954~inflammatory response | 40 | 6.55E-14 |
| GO:0006952~defense response | 54 | 1.70E-11 |
| GO:0042127~regulation of cell proliferation | 60 | 2.65E-11 |
| GO:0002237~response to molecule of bacterial origin | 15 | 1.06E-08 |
| GO:0001568~blood vessel development | 32 | 6.06E-08 |
| GO:0048732~gland development | 28 | 9.14E-08 |
| GO:0048514~blood vessel morphogenesis | 28 | 1.02E-07 |
| GO:0001944~vasculature development | 32 | 1.06E-07 |
| GO:0001817~regulation of cytokine production | 23 | 1.11E-07 |
| GO:0007178~transmembrane receptor protein serine/threonine kinase signaling pathway | 17 | 1.59E-07 |
| GO:0002684~positive regulation of immune system process | 27 | 8.10E-07 |
| GO:0006935~chemotaxis | 19 | 8.35E-07 |
| GO:0042330~taxis | 19 | 8.35E-07 |
| GO:0042325~regulation of phosphorylation | 33 | 9.51E-07 |
| GO:0051173~positive regulation of nitrogen compound metabolic process | 48 | 1.52E-06 |
| GO:0045893~positive regulation of transcription, DNA-dependent | 41 | 1.56E-06 |
| GO:0008284~positive regulation of cell proliferation | 32 | 1.77E-06 |
| GO:0045944~positive regulation of transcription from RNA polymerase II promoter | 37 | 1.85E-06 |
| GO:0051254~positive regulation of RNA metabolic process | 41 | 1.86E-06 |
| GO:0019220~regulation of phosphate metabolic process | 33 | 2.16E-06 |
| GO:0051174~regulation of phosphorus metabolic process | 33 | 2.16E-06 |
| GO:0032494~response to peptidoglycan | 7 | 2.36E-06 |
| GO:0002250~adaptive immune response | 16 | 2.50E-06 |
| GO:0002460~adaptive immune response based on somatic recombination of immune receptors built from immunoglobulin superfamily domains | 16 | 2.50E-06 |
| GO:0031328~positive regulation of cellular biosynthetic process | 49 | 2.53E-06 |
| GO:0007242~intracellular signaling cascade | 70 | 2.96E-06 |
| GO:0045941~positive regulation of transcription | 44 | 3.08E-06 |
| GO:0032496~response to lipopolysaccharide | 11 | 3.16E-06 |
| GO:0009891~positive regulation of biosynthetic process | 49 | 3.24E-06 |
| GO:0019221~cytokine-mediated signaling pathway | 13 | 3.50E-06 |
| GO:0045935~positive regulation of nucleobase, nucleoside, nucleotide and nucleic acid metabolic process | 46 | 3.59E-06 |
| GO:0010628~positive regulation of gene expression | 44 | 6.14E-06 |
| GO:0010557~positive regulation of macromolecule biosynthetic process | 46 | 9.85E-06 |
| GO:0006357~regulation of transcription from RNA polymerase II promoter | 51 | 1.10E-05 |
| GO:0008285~negative regulation of cell proliferation | 26 | 1.20E-05 |
| GO:0022612~gland morphogenesis | 15 | 1.24E-05 |
| GO:0001525~angiogenesis | 19 | 1.52E-05 |
| GO:0048584~positive regulation of response to stimulus | 23 | 1.62E-05 |
| GO:0045765~regulation of angiogenesis | 11 | 1.63E-05 |
| GO:0007179~transforming growth factor beta receptor signaling pathway | 11 | 2.97E-05 |
| GO:0001701~in utero embryonic development | 28 | 3.28E-05 |
| GO:0009968~negative regulation of signal transduction | 21 | 4.57E-05 |
| GO:0007167~enzyme linked receptor protein signaling pathway | 28 | 4.83E-05 |
| GO:0046888~negative regulation of hormone secretion | 7 | 5.06E-05 |
| GO:0035295~tube development | 27 | 7.12E-05 |
| GO:0030879~mammary gland development | 14 | 7.26E-05 |
| GO:0051240~positive regulation of multicellular organismal process | 20 | 7.37E-05 |
| GO:0031663~lipopolysaccharide-mediated signaling pathway | 6 | 8.88E-05 |
| GO:0010604~positive regulation of macromolecule metabolic process | 49 | 9.06E-05 |
| GO:0051048~negative regulation of secretion | 8 | 1.09E-04 |
| GO:0051216~cartilage development | 13 | 1.15E-04 |
| GO:0046883~regulation of hormone secretion | 9 | 1.15E-04 |
| GO:0051094~positive regulation of developmental process | 23 | 1.37E-04 |
| GO:0044093~positive regulation of molecular function | 29 | 1.37E-04 |
| GO:0009617~response to bacterium | 19 | 1.40E-04 |
| GO:0048660~regulation of smooth muscle cell proliferation | 7 | 1.42E-04 |
| GO:0030334~regulation of cell migration | 14 | 1.47E-04 |
| GO:0010648~negative regulation of cell communication | 21 | 1.49E-04 |
| GO:0045859~regulation of protein kinase activity | 21 | 1.49E-04 |
| GO:0010941~regulation of cell death | 44 | 1.79E-04 |
| GO:0051090~regulation of transcription factor activity | 11 | 1.86E-04 |
| GO:0051270~regulation of cell motion | 15 | 1.91E-04 |
| GO:0043549~regulation of kinase activity | 21 | 2.28E-04 |
| GO:0051051~negative regulation of transport | 12 | 2.34E-04 |
| GO:0042981~regulation of apoptosis | 43 | 2.37E-04 |
| GO:0001819~positive regulation of cytokine production | 11 | 2.84E-04 |
| GO:0001763~morphogenesis of a branching structure | 16 | 3.01E-04 |
| GO:0051252~regulation of RNA metabolic process | 92 | 3.01E-04 |
| GO:0051272~positive regulation of cell motion | 9 | 3.02E-04 |
| GO:0043009~chordate embryonic development | 35 | 3.10E-04 |
| GO:0043067~regulation of programmed cell death | 43 | 3.12E-04 |
| GO:0032675~regulation of interleukin-6 production | 8 | 3.44E-04 |
| GO:0009792~embryonic development ending in birth or egg hatching | 35 | 3.65E-04 |
| GO:0051338~regulation of transferase activity | 21 | 3.67E-04 |
| GO:0050867~positive regulation of cell activation | 14 | 3.78E-04 |
| GO:0009615~response to virus | 12 | 3.80E-04 |
| GO:0050865~regulation of cell activation | 18 | 3.90E-04 |
| GO:0035239~tube morphogenesis | 19 | 4.07E-04 |
| GO:0043123~positive regulation of I-kappaB kinase/NF-kappaB cascade | 8 | 4.21E-04 |
| GO:0060443~mammary gland morphogenesis | 8 | 4.21E-04 |
| GO:0006355~regulation of transcription, DNA-dependent | 90 | 4.42E-04 |
| GO:0010647~positive regulation of cell communication | 20 | 5.08E-04 |
| GO:0051092~positive regulation of NF-kappaB transcription factor activity | 7 | 5.40E-04 |
| GO:0007243~protein kinase cascade | 23 | 5.41E-04 |
| GO:0002221~pattern recognition receptor signaling pathway | 5 | 5.76E-04 |
| GO:0045766~positive regulation of angiogenesis | 6 | 5.80E-04 |
| GO:0002526~acute inflammatory response | 12 | 6.63E-04 |
| GO:0031349~positive regulation of defense response | 10 | 6.68E-04 |
| GO:0045449~regulation of transcription | 126 | 7.08E-04 |
| GO:0002687~positive regulation of leukocyte migration | 4 | 7.40E-04 |
| GO:0032755~positive regulation of interleukin-6 production | 6 | 7.75E-04 |
| GO:0040012~regulation of locomotion | 14 | 8.63E-04 |
| GO:0007265~Ras protein signal transduction | 10 | 8.65E-04 |
| GO:0030335~positive regulation of cell migration | 8 | 8.73E-04 |
| GO:0032642~regulation of chemokine production | 5 | 8.75E-04 |
| GO:0045580~regulation of T cell differentiation | 9 | 9.15E-04 |
| GO:0051046~regulation of secretion | 15 | 9.49E-04 |
| GO:0002520~immune system development | 26 | 9.59E-04 |
| GO:0002694~regulation of leukocyte activation | 17 | 9.66E-04 |
| GO:0051329~interphase of mitotic cell cycle | 9 | 0.001053 |
| GO:0002696~positive regulation of leukocyte activation | 13 | 0.001079 |
| GO:0048534~hemopoietic or lymphoid organ development | 25 | 0.00109 |
| GO:0009967~positive regulation of signal transduction | 18 | 0.001189 |
| GO:0051101~regulation of DNA binding | 11 | 0.001198 |
| GO:0002758~innate immune response-activating signal transduction | 5 | 0.001268 |
| GO:0002697~regulation of immune effector process | 12 | 0.001335 |
| GO:0051249~regulation of lymphocyte activation | 16 | 0.00134 |
| GO:0051325~interphase | 9 | 0.001379 |
| GO:0043122~regulation of I-kappaB kinase/NF-kappaB cascade | 8 | 0.001415 |
| GO:0006953~acute-phase response | 7 | 0.001509 |
| GO:0007507~heart development | 21 | 0.001523 |
| GO:0060341~regulation of cellular localization | 17 | 0.001548 |
| GO:0042035~regulation of cytokine biosynthetic process | 10 | 0.001567 |
| GO:0016477~cell migration | 22 | 0.001577 |
| GO:0051098~regulation of binding | 12 | 0.001606 |
| GO:0031327~negative regulation of cellular biosynthetic process | 33 | 0.001773 |
| GO:0007431~salivary gland development | 7 | 0.001804 |
| GO:0043085~positive regulation of catalytic activity | 23 | 0.002015 |
| GO:0009890~negative regulation of biosynthetic process | 33 | 0.002042 |
| GO:0046637~regulation of alpha-beta T cell differentiation | 6 | 0.002065 |
| GO:0048754~branching morphogenesis of a tube | 12 | 0.002097 |
| GO:0050778~positive regulation of immune response | 15 | 0.002141 |
| GO:0050670~regulation of lymphocyte proliferation | 11 | 0.002182 |
| GO:0032944~regulation of mononuclear cell proliferation | 11 | 0.002182 |
| GO:0001570~vasculogenesis | 8 | 0.002188 |
| GO:0050900~leukocyte migration | 8 | 0.002188 |
| GO:0051251~positive regulation of lymphocyte activation | 12 | 0.002285 |
| GO:0002218~activation of innate immune response | 5 | 0.002395 |
| GO:0002224~toll-like receptor signaling pathway | 4 | 0.002428 |
| GO:0040017~positive regulation of locomotion | 8 | 0.002507 |
| GO:0002822~regulation of adaptive immune response based on somatic recombination of immune receptors built from immunoglobulin superfamily domains | 9 | 0.00255 |
| GO:0002819~regulation of adaptive immune response | 9 | 0.00255 |
| GO:0070663~regulation of leukocyte proliferation | 11 | 0.002626 |
| GO:0030099~myeloid cell differentiation | 12 | 0.002703 |
| GO:0010942~positive regulation of cell death | 22 | 0.002877 |
| GO:0051091~positive regulation of transcription factor activity | 7 | 0.002955 |
| GO:0002252~immune effector process | 14 | 0.002981 |
| GO:0001569~patterning of blood vessels | 6 | 0.003104 |
| GO:0048661~positive regulation of smooth muscle cell proliferation | 5 | 0.003156 |
| GO:0045619~regulation of lymphocyte differentiation | 9 | 0.003192 |
| GO:0006915~apoptosis | 34 | 0.003235 |
| GO:0007626~locomotory behavior | 21 | 0.003418 |
| GO:0010033~response to organic substance | 36 | 0.0036 |
| GO:0010605~negative regulation of macromolecule metabolic process | 36 | 0.00372 |
| GO:0006875~cellular metal ion homeostasis | 12 | 0.003722 |
| GO:0048589~developmental growth | 12 | 0.003722 |
| GO:0042088~T-helper 1 type immune response | 4 | 0.003761 |
| GO:0002685~regulation of leukocyte migration | 4 | 0.003761 |
| GO:0040007~growth | 18 | 0.004032 |
| GO:0042110~T cell activation | 13 | 0.004156 |
| GO:0012501~programmed cell death | 34 | 0.004207 |
| GO:0044092~negative regulation of molecular function | 14 | 0.004455 |
| GO:0051050~positive regulation of transport | 14 | 0.004455 |
| GO:0050673~epithelial cell proliferation | 6 | 0.004476 |
| GO:0001775~cell activation | 21 | 0.004722 |
| GO:0031399~regulation of protein modification process | 16 | 0.005008 |
| GO:0051726~regulation of cell cycle | 19 | 0.005096 |
| GO:0010332~response to gamma radiation | 5 | 0.005139 |
| GO:0032946~positive regulation of mononuclear cell proliferation | 8 | 0.005237 |
| GO:0050671~positive regulation of lymphocyte proliferation | 8 | 0.005237 |
| GO:0007435~salivary gland morphogenesis | 6 | 0.005302 |
| GO:0001916~positive regulation of T cell mediated cytotoxicity | 4 | 0.005463 |
| GO:0043068~positive regulation of programmed cell death | 21 | 0.005639 |
| GO:0030097~hemopoiesis | 21 | 0.005769 |
| GO:0055065~metal ion homeostasis | 12 | 0.005804 |
| GO:0030003~cellular cation homeostasis | 15 | 0.00587 |
| GO:0032103~positive regulation of response to external stimulus | 7 | 0.00598 |
| GO:0010035~response to inorganic substance | 11 | 0.006046 |
| GO:0044057~regulation of system process | 18 | 0.006048 |
| GO:0042098~T cell proliferation | 6 | 0.006231 |
| GO:0000060~protein import into nucleus, translocation | 5 | 0.006386 |
| GO:0070665~positive regulation of leukocyte proliferation | 8 | 0.006516 |
| GO:0050863~regulation of T cell activation | 12 | 0.006672 |
| GO:0045860~positive regulation of protein kinase activity | 13 | 0.007068 |
| GO:0040008~regulation of growth | 21 | 0.007263 |
| GO:0021983~pituitary gland development | 6 | 0.007269 |
| GO:0010558~negative regulation of macromolecule biosynthetic process | 30 | 0.007541 |
| GO:0035272~exocrine system development | 7 | 0.007667 |
| GO:0060444~branching involved in mammary gland duct morphogenesis | 5 | 0.007818 |
| GO:0048535~lymph node development | 5 | 0.007818 |
| GO:0046638~positive regulation of alpha-beta T cell differentiation | 5 | 0.007818 |
| GO:0051172~negative regulation of nitrogen compound metabolic process | 29 | 0.007937 |
| GO:0043388~positive regulation of DNA binding | 7 | 0.008628 |
| GO:0048511~rhythmic process | 11 | 0.009352 |
| GO:0048145~regulation of fibroblast proliferation | 5 | 0.009446 |
| GO:0045785~positive regulation of cell adhesion | 7 | 0.009672 |
| GO:0007595~lactation | 6 | 0.009693 |
| GO:0043405~regulation of MAP kinase activity | 10 | 0.009844 |
| GO:0043370~regulation of CD4-positive, alpha beta T cell differentiation | 4 | 0.010063 |
| GO:0001914~regulation of T cell mediated cytotoxicity | 4 | 0.010063 |
| GO:0033674~positive regulation of kinase activity | 13 | 0.010158 |
| GO:0070423~nucleotide-binding oligomerization domain containing signaling pathway | 3 | 0.010443 |
| GO:0070431~nucleotide-binding oligomerization domain containing 2 signaling pathway | 3 | 0.010443 |
| GO:0043065~positive regulation of apoptosis | 20 | 0.01066 |
| GO:0007155~cell adhesion | 37 | 0.010677 |
| GO:0051241~negative regulation of multicellular organismal process | 11 | 0.010716 |
| GO:0000122~negative regulation of transcription from RNA polymerase II promoter | 19 | 0.010811 |
| GO:0022610~biological adhesion | 37 | 0.010891 |
| GO:0051674~localization of cell | 22 | 0.010937 |
| GO:0048870~cell motility | 22 | 0.010937 |
| GO:0006468~protein amino acid phosphorylation | 41 | 0.011201 |
| GO:0002253~activation of immune response | 10 | 0.011407 |
| GO:0008219~cell death | 34 | 0.011412 |
| GO:0009628~response to abiotic stimulus | 20 | 0.01175 |
| GO:0051099~positive regulation of binding | 7 | 0.012025 |
| GO:0019882~antigen processing and presentation | 10 | 0.012255 |
| GO:0045934~negative regulation of nucleobase, nucleoside, nucleotide and nucleic acid metabolic process | 28 | 0.012483 |
| GO:0043330~response to exogenous dsRNA | 4 | 0.012995 |
| GO:0032101~regulation of response to external stimulus | 11 | 0.013037 |
| GO:0055080~cation homeostasis | 16 | 0.013131 |
| GO:0010740~positive regulation of protein kinase cascade | 10 | 0.013149 |
| GO:0050796~regulation of insulin secretion | 5 | 0.013327 |
| GO:0045088~regulation of innate immune response | 7 | 0.013341 |
| GO:0051347~positive regulation of transferase activity | 13 | 0.013455 |
| GO:0045321~leukocyte activation | 18 | 0.013608 |
| GO:0003007~heart morphogenesis | 9 | 0.013832 |
| GO:0046634~regulation of alpha-beta T cell activation | 6 | 0.014288 |
| GO:0002821~positive regulation of adaptive immune response | 6 | 0.014288 |
| GO:0002824~positive regulation of adaptive immune response based on somatic recombination of immune receptors built from immunoglobulin superfamily domains | 6 | 0.014288 |
| GO:0048771~tissue remodeling | 7 | 0.014755 |
| GO:0043066~negative regulation of apoptosis | 19 | 0.01492 |
| GO:0001932~regulation of protein amino acid phosphorylation | 12 | 0.015038 |
| GO:0016265~death | 34 | 0.01558 |
| GO:0046631~alpha-beta T cell activation | 5 | 0.015597 |
| GO:0050766~positive regulation of phagocytosis | 5 | 0.015597 |
| GO:0007610~behavior | 28 | 0.015827 |
| GO:0002449~lymphocyte mediated immunity | 9 | 0.01607 |
| GO:0006874~cellular calcium ion homeostasis | 10 | 0.016122 |
| GO:0010627~regulation of protein kinase cascade | 14 | 0.016232 |
| GO:0002757~immune response-activating signal transduction | 7 | 0.016269 |
| GO:0010165~response to X-ray | 4 | 0.016362 |
| GO:0043900~regulation of multi-organism process | 4 | 0.016362 |
| GO:0042089~cytokine biosynthetic process | 4 | 0.016362 |
| GO:0002711~positive regulation of T cell mediated immunity | 4 | 0.016362 |
| GO:0070391~response to lipoteichoic acid | 3 | 0.016912 |
| GO:0032495~response to muramyl dipeptide | 3 | 0.016912 |
| GO:0046426~negative regulation of JAK-STAT cascade | 3 | 0.016912 |
| GO:0060541~respiratory system development | 12 | 0.017772 |
| GO:0016064~immunoglobulin mediated immune response | 8 | 0.018017 |
| GO:0043069~negative regulation of programmed cell death | 19 | 0.018046 |
| GO:0070507~regulation of microtubule cytoskeleton organization | 6 | 0.018051 |
| GO:0032268~regulation of cellular protein metabolic process | 21 | 0.018092 |
| GO:0050729~positive regulation of inflammatory response | 5 | 0.018095 |
| GO:0060548~negative regulation of cell death | 19 | 0.018695 |
| GO:0045597~positive regulation of cell differentiation | 15 | 0.018814 |
| GO:0001666~response to hypoxia | 8 | 0.019506 |
| GO:0030155~regulation of cell adhesion | 10 | 0.01956 |
| GO:0003018~vascular process in circulatory system | 7 | 0.019612 |
| GO:0008283~cell proliferation | 19 | 0.020096 |
| GO:0042771~DNA damage response, signal transduction by p53 class mediator resulting in induction of apoptosis | 4 | 0.020171 |
| GO:0046887~positive regulation of hormone secretion | 4 | 0.020171 |
| GO:0042107~cytokine metabolic process | 4 | 0.020171 |
| GO:0055074~calcium ion homeostasis | 10 | 0.020816 |
| GO:0006940~regulation of smooth muscle contraction | 5 | 0.020829 |
| GO:0050764~regulation of phagocytosis | 5 | 0.020829 |
| GO:0070482~response to oxygen levels | 8 | 0.02108 |
| GO:0019724~B cell mediated immunity | 8 | 0.02108 |
| GO:0033673~negative regulation of kinase activity | 7 | 0.021447 |
| GO:0006469~negative regulation of protein kinase activity | 7 | 0.021447 |
| GO:0021536~diencephalon development | 6 | 0.022415 |
| GO:0050870~positive regulation of T cell activation | 8 | 0.022739 |
| GO:0002764~immune response-regulating signal transduction | 7 | 0.023394 |
| GO:0055066~di-, tri-valent inorganic cation homeostasis | 13 | 0.023521 |
| GO:0030323~respiratory tube development | 11 | 0.023536 |
| GO:0002791~regulation of peptide secretion | 5 | 0.023802 |
| GO:0046635~positive regulation of alpha-beta T cell activation | 5 | 0.023802 |
| GO:0060603~mammary gland duct morphogenesis | 5 | 0.023802 |
| GO:0032760~positive regulation of tumor necrosis factor production | 4 | 0.024425 |
| GO:0019229~regulation of vasoconstriction | 4 | 0.024425 |
| GO:0032655~regulation of interleukin-12 production | 4 | 0.024425 |
| GO:0060438~trachea development | 3 | 0.024651 |
| GO:0002467~germinal center formation | 3 | 0.024651 |
| GO:0045892~negative regulation of transcription, DNA-dependent | 22 | 0.024836 |
| GO:0000165~MAPKKK cascade | 11 | 0.024848 |
| GO:0048667~cell morphogenesis involved in neuron differentiation | 15 | 0.025405 |
| GO:0051348~negative regulation of transferase activity | 7 | 0.025457 |
| GO:0010038~response to metal ion | 7 | 0.025457 |
| GO:0050730~regulation of peptidyl-tyrosine phosphorylation | 7 | 0.025457 |
| GO:0016310~phosphorylation | 43 | 0.025634 |
| GO:0060627~regulation of vesicle-mediated transport | 9 | 0.025966 |
| GO:0007565~female pregnancy | 8 | 0.026323 |
| GO:0051253~negative regulation of RNA metabolic process | 22 | 0.026411 |
| GO:0006928~cell motion | 25 | 0.026906 |
| GO:0030595~leukocyte chemotaxis | 5 | 0.027019 |
| GO:0010811~positive regulation of cell-substrate adhesion | 5 | 0.027019 |
| GO:0000910~cytokinesis | 5 | 0.027019 |
| GO:0060326~cell chemotaxis | 5 | 0.027019 |
| GO:0007266~Rho protein signal transduction | 5 | 0.027019 |
| GO:0032680~regulation of tumor necrosis factor production | 5 | 0.027019 |
| GO:0010810~regulation of cell-substrate adhesion | 6 | 0.02741 |
| GO:0012502~induction of programmed cell death | 14 | 0.028054 |
| GO:0006917~induction of apoptosis | 14 | 0.028054 |
| GO:0042592~homeostatic process | 36 | 0.028907 |
| GO:0046330~positive regulation of JNK cascade | 4 | 0.029123 |
| GO:0070304~positive regulation of stress-activated protein kinase signaling pathway | 4 | 0.029123 |
| GO:0060560~developmental growth involved in morphogenesis | 4 | 0.029123 |
| GO:0030005~cellular di-, tri-valent inorganic cation homeostasis | 12 | 0.029575 |
| GO:0043086~negative regulation of catalytic activity | 10 | 0.029592 |
| GO:0016481~negative regulation of transcription | 25 | 0.030146 |
| GO:0006937~regulation of muscle contraction | 6 | 0.030152 |
| GO:0002703~regulation of leukocyte mediated immunity | 8 | 0.030275 |
| GO:0000082~G1/S transition of mitotic cell cycle | 5 | 0.030484 |
| GO:0031110~regulation of microtubule polymerization or depolymerization | 5 | 0.030484 |
| GO:0048568~embryonic organ development | 18 | 0.030857 |
| GO:0046651~lymphocyte proliferation | 6 | 0.03306 |
| GO:0016202~regulation of striated muscle tissue development | 6 | 0.03306 |
| GO:0001890~placenta development | 9 | 0.033212 |
| GO:0046627~negative regulation of insulin receptor signaling pathway | 3 | 0.03354 |
| GO:0045073~regulation of chemokine biosynthetic process | 3 | 0.03354 |
| GO:0045987~positive regulation of smooth muscle contraction | 3 | 0.03354 |
| GO:0045622~regulation of T-helper cell differentiation | 3 | 0.03354 |
| GO:0060664~epithelial cell proliferation involved in salivary gland morphogenesis | 3 | 0.03354 |
| GO:0033043~regulation of organelle organization | 13 | 0.033731 |
| GO:0009620~response to fungus | 4 | 0.034263 |
| GO:0035270~endocrine system development | 8 | 0.034607 |
| GO:0007050~cell cycle arrest | 7 | 0.034905 |
| GO:0050727~regulation of inflammatory response | 7 | 0.034905 |
| GO:0042770~DNA damage response, signal transduction | 7 | 0.034905 |
| GO:0003006~reproductive developmental process | 19 | 0.035822 |
| GO:0048634~regulation of muscle development | 6 | 0.036138 |
| GO:0070661~leukocyte proliferation | 6 | 0.036138 |
| GO:0032943~mononuclear cell proliferation | 6 | 0.036138 |
| GO:0051130~positive regulation of cellular component organization | 11 | 0.037284 |
| GO:0002443~leukocyte mediated immunity | 9 | 0.037305 |
| GO:0000077~DNA damage checkpoint | 5 | 0.038164 |
| GO:0017015~regulation of transforming growth factor beta receptor signaling pathway | 5 | 0.038164 |
| GO:0006350~transcription | 92 | 0.03847 |
| GO:0001501~skeletal system development | 20 | 0.038529 |
| GO:0002709~regulation of T cell mediated immunity | 4 | 0.039838 |
| GO:0000904~cell morphogenesis involved in differentiation | 16 | 0.040286 |
| GO:0045087~innate immune response | 10 | 0.040676 |
| GO:0048469~cell maturation | 8 | 0.041845 |
| GO:0048878~chemical homeostasis | 24 | 0.04201 |
| GO:0002521~leukocyte differentiation | 12 | 0.042418 |
| GO:0032886~regulation of microtubule-based process | 6 | 0.042806 |
| GO:0055069~zinc ion homeostasis | 3 | 0.043467 |
| GO:0060429~epithelium development | 19 | 0.044153 |
| GO:0009314~response to radiation | 12 | 0.044257 |
| GO:0030217~T cell differentiation | 8 | 0.04446 |
| GO:0046632~alpha-beta T cell differentiation | 4 | 0.045842 |
| GO:0030330~DNA damage response, signal transduction by p53 class mediator | 4 | 0.045842 |
| GO:0048666~neuron development | 20 | 0.046281 |
| GO:0001892~embryonic placenta development | 7 | 0.046359 |
| GO:0002699~positive regulation of immune effector process | 6 | 0.046399 |
| GO:0007254~JNK cascade | 5 | 0.046853 |
| GO:0034097~response to cytokine stimulus | 5 | 0.046853 |
| GO:0045582~positive regulation of T cell differentiation | 5 | 0.046853 |
| GO:0030522~intracellular receptor-mediated signaling pathway | 5 | 0.046853 |
| GO:0030888~regulation of B cell proliferation | 5 | 0.046853 |
| GO:0007409~axonogenesis | 13 | 0.04861 |
| GO:0031175~neuron projection development | 16 | 0.049317 |
| GO:0030324~lung development | 10 | 0.049452 |
| GO:0010629~negative regulation of gene expression | 26 | 0.049511 |

**Supplementary Table 29.**  Biological processes significantly enriched in the overlapping genes between the immune-related module (S_Co_M16) from combined data and that from LPS treated mouse model (LPS_3h_M2)

| **Term** | **Count** | **PValue** |
| --- | --- | --- |
| GO:0009611~response to wounding | 15 | 2.16E-08 |
| GO:0001817~regulation of cytokine production | 10 | 4.97E-08 |
| GO:0006954~inflammatory response | 12 | 7.37E-08 |
| GO:0048584~positive regulation of response to stimulus | 10 | 4.77E-07 |
| GO:0031349~positive regulation of defense response | 7 | 5.25E-07 |
| GO:0034097~response to cytokine stimulus | 7 | 8.42E-07 |
| GO:0006952~defense response | 14 | 9.70E-07 |
| GO:0002237~response to molecule of bacterial origin | 7 | 1.39E-06 |
| GO:0032101~regulation of response to external stimulus | 8 | 3.88E-06 |
| GO:0010033~response to organic substance | 14 | 5.65E-06 |
| GO:0032496~response to lipopolysaccharide | 6 | 1.60E-05 |
| GO:0001819~positive regulation of cytokine production | 6 | 3.41E-05 |
| GO:0002221~pattern recognition receptor signaling pathway | 4 | 4.38E-05 |
| GO:0002684~positive regulation of immune system process | 8 | 5.32E-05 |
| GO:0002758~innate immune response-activating signal transduction | 4 | 6.21E-05 |
| GO:0002218~activation of innate immune response | 4 | 6.21E-05 |
| GO:0042127~regulation of cell proliferation | 13 | 7.20E-05 |
| GO:0032103~positive regulation of response to external stimulus | 5 | 1.39E-04 |
| GO:0009617~response to bacterium | 7 | 1.40E-04 |
| GO:0019221~cytokine-mediated signaling pathway | 5 | 1.97E-04 |
| GO:0002755~MyD88-dependent toll-like receptor signaling pathway | 3 | 2.50E-04 |
| GO:0050727~regulation of inflammatory response | 5 | 2.71E-04 |
| GO:0050778~positive regulation of immune response | 6 | 3.25E-04 |
| GO:0051240~positive regulation of multicellular organismal process | 7 | 4.94E-04 |
| GO:0002253~activation of immune response | 5 | 6.09E-04 |
| GO:0051092~positive regulation of NF-kappaB transcription factor activity | 4 | 6.41E-04 |
| GO:0051272~positive regulation of cell motion | 5 | 7.13E-04 |
| GO:0010605~negative regulation of macromolecule metabolic process | 11 | 7.64E-04 |
| GO:0048585~negative regulation of response to stimulus | 5 | 7.69E-04 |
| GO:0019220~regulation of phosphate metabolic process | 9 | 8.05E-04 |
| GO:0051174~regulation of phosphorus metabolic process | 9 | 8.05E-04 |
| GO:0045089~positive regulation of innate immune response | 4 | 8.99E-04 |
| GO:0010810~regulation of cell-substrate adhesion | 4 | 8.99E-04 |
| GO:0051094~positive regulation of developmental process | 7 | 9.79E-04 |
| GO:0009615~response to virus | 5 | 0.001 |
| GO:0002757~immune response-activating signal transduction | 4 | 0.001 |
| GO:0045088~regulation of innate immune response | 4 | 0.001 |
| GO:0002224~toll-like receptor signaling pathway | 3 | 0.001 |
| GO:0002764~immune response-regulating signal transduction | 4 | 0.002 |
| GO:0051091~positive regulation of transcription factor activity | 4 | 0.002 |
| GO:0045785~positive regulation of cell adhesion | 4 | 0.002 |
| GO:0009968~negative regulation of signal transduction | 6 | 0.002 |
| GO:0042177~negative regulation of protein catabolic process | 3 | 0.002 |
| GO:0032655~regulation of interleukin-12 production | 3 | 0.002 |
| GO:0070555~response to interleukin-1 | 3 | 0.002 |
| GO:0001666~response to hypoxia | 5 | 0.002 |
| GO:0030155~regulation of cell adhesion | 5 | 0.002 |
| GO:0070482~response to oxygen levels | 5 | 0.003 |
| GO:0043405~regulation of MAP kinase activity | 5 | 0.003 |
| GO:0042325~regulation of phosphorylation | 8 | 0.003 |
| GO:0043388~positive regulation of DNA binding | 4 | 0.003 |
| GO:0032570~response to progesterone stimulus | 3 | 0.003 |
| GO:0010648~negative regulation of cell communication | 6 | 0.004 |
| GO:0010627~regulation of protein kinase cascade | 6 | 0.004 |
| GO:0051098~regulation of binding | 5 | 0.004 |
| GO:0051099~positive regulation of binding | 4 | 0.004 |
| GO:0007243~protein kinase cascade | 7 | 0.004 |
| GO:0045766~positive regulation of angiogenesis | 3 | 0.005 |
| GO:0042981~regulation of apoptosis | 10 | 0.005 |
| GO:0001952~regulation of cell-matrix adhesion | 3 | 0.006 |
| GO:0043067~regulation of programmed cell death | 10 | 0.006 |
| GO:0010941~regulation of cell death | 10 | 0.006 |
| GO:0030335~positive regulation of cell migration | 4 | 0.006 |
| GO:0010811~positive regulation of cell-substrate adhesion | 3 | 0.006 |
| GO:0001775~cell activation | 6 | 0.007 |
| GO:0050729~positive regulation of inflammatory response | 3 | 0.007 |
| GO:0031328~positive regulation of cellular biosynthetic process | 9 | 0.007 |
| GO:0008284~positive regulation of cell proliferation | 7 | 0.007 |
| GO:0032680~regulation of tumor necrosis factor production | 3 | 0.007 |
| GO:0009967~positive regulation of signal transduction | 6 | 0.007 |
| GO:0009891~positive regulation of biosynthetic process | 9 | 0.007 |
| GO:0040017~positive regulation of locomotion | 4 | 0.008 |
| GO:0051270~regulation of cell motion | 5 | 0.008 |
| GO:0051090~regulation of transcription factor activity | 4 | 0.009 |
| GO:0001501~skeletal system development | 6 | 0.010 |
| GO:0044093~positive regulation of molecular function | 8 | 0.010 |
| GO:0009895~negative regulation of catabolic process | 3 | 0.010 |
| GO:0001818~negative regulation of cytokine production | 3 | 0.011 |
| GO:0007259~JAK-STAT cascade | 3 | 0.011 |
| GO:0010647~positive regulation of cell communication | 6 | 0.011 |
| GO:0002238~response to molecule of fungal origin | 2 | 0.012 |
| GO:0040008~regulation of growth | 6 | 0.013 |
| GO:0045893~positive regulation of transcription, DNA-dependent | 7 | 0.014 |
| GO:0051101~regulation of DNA binding | 4 | 0.014 |
| GO:0045859~regulation of protein kinase activity | 6 | 0.014 |
| GO:0051254~positive regulation of RNA metabolic process | 7 | 0.014 |
| GO:0045597~positive regulation of cell differentiation | 5 | 0.015 |
| GO:0043066~negative regulation of apoptosis | 6 | 0.015 |
| GO:0043549~regulation of kinase activity | 6 | 0.016 |
| GO:0043069~negative regulation of programmed cell death | 6 | 0.016 |
| GO:0060548~negative regulation of cell death | 6 | 0.016 |
| GO:0051173~positive regulation of nitrogen compound metabolic process | 8 | 0.016 |
| GO:0002701~negative regulation of production of molecular mediator of immune response | 2 | 0.016 |
| GO:0002719~negative regulation of cytokine production during immune response | 2 | 0.016 |
| GO:0014742~positive regulation of muscle hypertrophy | 2 | 0.016 |
| GO:0010613~positive regulation of cardiac muscle hypertrophy | 2 | 0.016 |
| GO:0008285~negative regulation of cell proliferation | 6 | 0.017 |
| GO:0010557~positive regulation of macromolecule biosynthetic process | 8 | 0.018 |
| GO:0051338~regulation of transferase activity | 6 | 0.019 |
| GO:0045087~innate immune response | 4 | 0.019 |
| GO:0042176~regulation of protein catabolic process | 3 | 0.020 |
| GO:0032695~negative regulation of interleukin-12 production | 2 | 0.021 |
| GO:0050878~regulation of body fluid levels | 4 | 0.021 |
| GO:0006955~immune response | 8 | 0.023 |
| GO:0010604~positive regulation of macromolecule metabolic process | 9 | 0.024 |
| GO:0010611~regulation of cardiac muscle hypertrophy | 2 | 0.025 |
| GO:0010558~negative regulation of macromolecule biosynthetic process | 7 | 0.025 |
| GO:0007346~regulation of mitotic cell cycle | 4 | 0.025 |
| GO:0031327~negative regulation of cellular biosynthetic process | 7 | 0.028 |
| GO:0045765~regulation of angiogenesis | 3 | 0.028 |
| GO:0045941~positive regulation of transcription | 7 | 0.028 |
| GO:0014743~regulation of muscle hypertrophy | 2 | 0.029 |
| GO:0051241~negative regulation of multicellular organismal process | 4 | 0.030 |
| GO:0009890~negative regulation of biosynthetic process | 7 | 0.030 |
| GO:0010740~positive regulation of protein kinase cascade | 4 | 0.032 |
| GO:0010628~positive regulation of gene expression | 7 | 0.032 |
| GO:0051893~regulation of focal adhesion formation | 2 | 0.033 |
| GO:0001960~negative regulation of cytokine-mediated signaling pathway | 2 | 0.033 |
| GO:0030334~regulation of cell migration | 4 | 0.033 |
| GO:0001932~regulation of protein amino acid phosphorylation | 4 | 0.035 |
| GO:0052200~response to host defenses | 2 | 0.037 |
| GO:0052173~response to defenses of other organism during symbiotic interaction | 2 | 0.037 |
| GO:0075136~response to host | 2 | 0.037 |
| GO:0007166~cell surface receptor linked signal transduction | 14 | 0.037 |
| GO:0043502~regulation of muscle adaptation | 2 | 0.041 |
| GO:0001953~negative regulation of cell-matrix adhesion | 2 | 0.041 |
| GO:0051248~negative regulation of protein metabolic process | 4 | 0.042 |
| GO:0045935~positive regulation of nucleobase, nucleoside, nucleotide and nucleic acid metabolic process | 7 | 0.043 |
| GO:0042060~wound healing | 4 | 0.045 |
| GO:0010812~negative regulation of cell-substrate adhesion | 2 | 0.045 |
| GO:0048545~response to steroid hormone stimulus | 4 | 0.045 |
| GO:0040012~regulation of locomotion | 4 | 0.045 |
| GO:0032268~regulation of cellular protein metabolic process | 6 | 0.046 |
| GO:0006928~cell motion | 6 | 0.046 |
| GO:0001558~regulation of cell growth | 4 | 0.046 |
| GO:0002683~negative regulation of immune system process | 3 | 0.046 |
| GO:0031667~response to nutrient levels | 4 | 0.048 |
| GO:0002675~positive regulation of acute inflammatory response | 2 | 0.049 |
| GO:0048821~erythrocyte development | 2 | 0.049 |
