# Supplementary material for: Transcriptome sequencing of the choroid plexus in schizophrenia
Source: Transl Psychiatry. 2016 Nov 29;6(11):e964–. doi: 10.1038/tp.2016.229 (PMC5290353; doi:10.1038/tp.2016.229)
Supplement: Supplementary Figure 1 [file tp2016229x2.docx]

**Supplementary Figure 1**

**a**

**b**


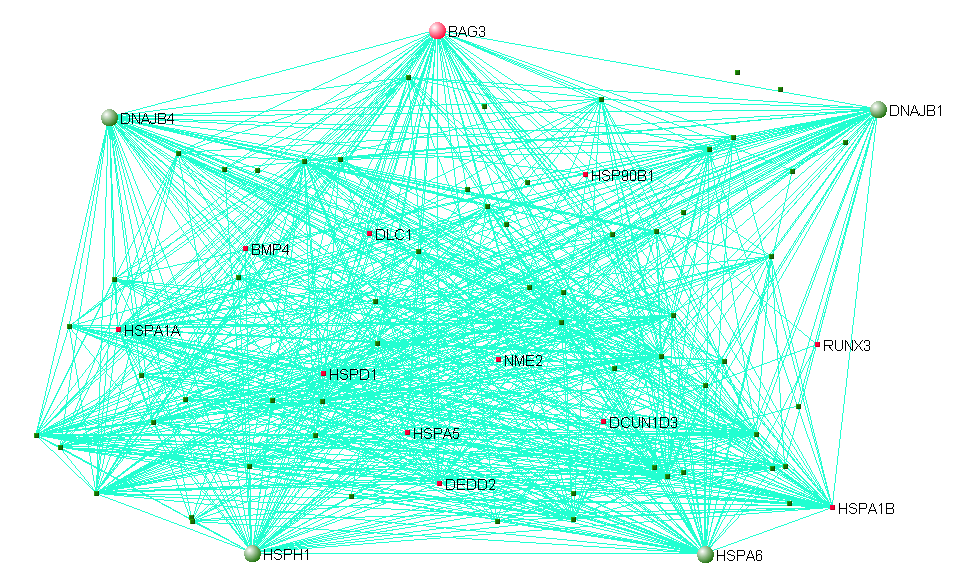


**Supplementary Figure 1. Co-expression module for schizophrenia in the choroid**

**plexus**

Visualization of the co-expression module (SCH_only_M7) which was built solely from schizophrenia data and was only weakly preserved in unaffected controls and therefore likely to be schizophrenia specific (a) and major biological processes (Gene ontology) enriched in the genes in the co-expression module (b). All network connections with topological overlap above the threshold of 0.2 were visualized using VisANT[^1^](#_ENREF_1). The hub genes are larger circles in the network. Genes related to apoptosis are red.

# REFERENCE

1. Hu Z, Mellor J, Wu J, DeLisi C. VisANT: an online visualization and analysis tool for biological interaction data. *BMC bioinformatics* 2004; **5:** 17.
