# Supplementary material for: Transcriptome sequencing of the choroid plexus in schizophrenia
Source: Transl Psychiatry. 2016 Nov 29;6(11):e964–. doi: 10.1038/tp.2016.229 (PMC5290353; doi:10.1038/tp.2016.229)
Supplement: Supplementary Figure 2 [file tp2016229x3.docx]

**Supplementary Figure 2**


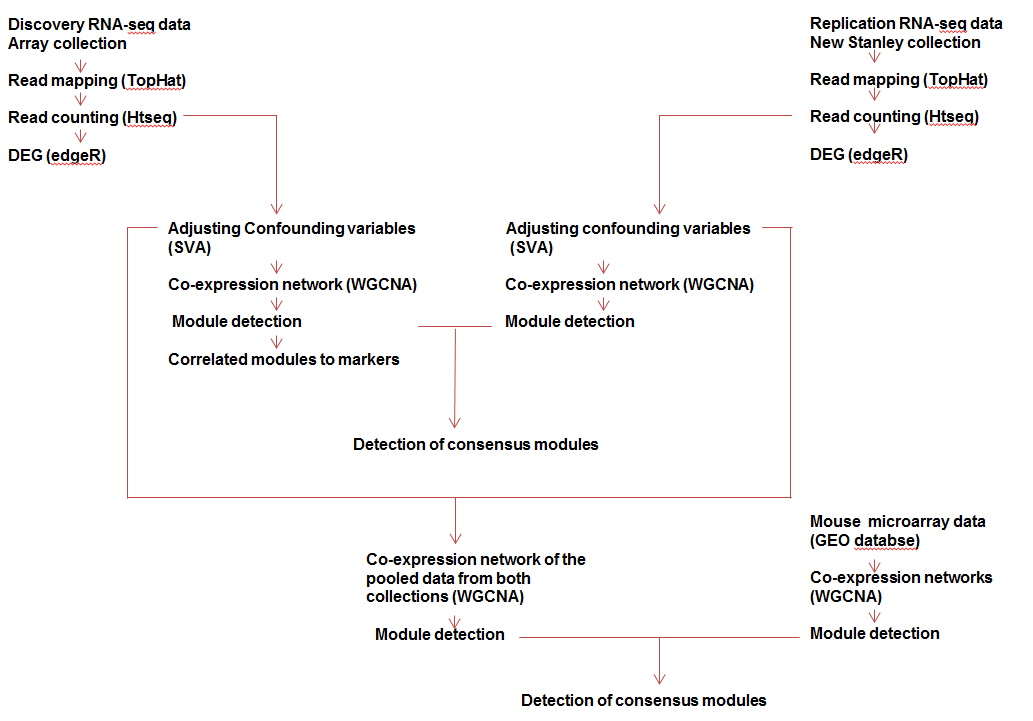


**Supplementary Figure 2. Overall flowchart of data analysis**

Flowchart shows the procedures for differentially expressed genes (DEG), detection of co-expression modules associated with schizophrenia and detection of consensus modules between two co-expression networks. Software and R-packages used for each corresponding analysis are in parentheses.
