# Supplementary material for: Transcriptome sequencing of the choroid plexus in schizophrenia
Source: Transl Psychiatry. 2016 Nov 29;6(11):e964–. doi: 10.1038/tp.2016.229 (PMC5290353; doi:10.1038/tp.2016.229)
Supplement: Supplementary Table 30 [file tp2016229x4.docx]

**Supplementary Table S30. Gene symbols of each co-expression modules**

**S_M16:** *AASS ABCA1 ABL1 ACIN1 ACKR3 ACO1 ACVR1B ADAMTS1 ADAMTS15 ADAMTS4 ADORA2A-AS1 AFAP1 AKAP12 AKR1B10 AKR1C2 ALDH7A1 ALG9 ALOX15B ALOX5 ALPL ANAPC5 ANKMY2 ANO2 APCDD1 APOL6 ARHGAP6 ARHGEF19 ARID3A ARSJ ATAD2 ATP1B3 ATP8B1 AXL AZI2 B4GALT1 B4GALT5 BATF BCL2L11 BCL3 BCL6 BDKRB2 BICC1 BLCAP BMP1 BMP8A BMP8B BMPER BNIP3 BNIP3L BPHL BRE-AS1 BTG1 BZW1 C14orf93 C17orf96 C1R C1RL C1RL-AS1 C1orf162 C2CD4A C3 C3orf36 C5AR2 C6orf195 CAMK2D CAPZA1 CASC10 CASP1 CASP4 CCDC174 CCDC71L CCNI CCNL1 CCR7 CD163 CD1D CD209 CD274 CD300A CD38 CD44 CD55 CD72 CD9 CDC37L1 CDC42SE1 CDC6 CDC7 CDH11 CDK2 CDV3 CEBPD CFB CFI CHI3L2 CHMP1B CHSY1 CITED2 CLCN1 CLEC4D CLEC4E CLIC4 CLK1 CLMP CMTM8 CNTN4 COL12A1 COL8A1 COMP CR1 CREB3L1 CREM CRISPLD2 CRTC3 CTC1 CTSL CXCL6 CYP1B1 CYP21A2 CYP4B1 DBF4 DDIT4 DENND3 DGAT2 DGKA DHCR7 DIO3 DIO3OS DIRC3 DIXDC1 DLGAP1-AS1 DNAJC12 DRAM1 DSE DTWD1 DTX3L DUSP1 ECEL1 EDEM1 EDNRA EFCAB13 EFNA1 EGFR EIF1B ELAC2 ELF1 ELF2 ELF4 ELK1 ELL2 ELP2 EMR2 ENPEP ENTPD6 EPB41L5 EPHX2 EPSTI1 ERMAP ESYT3 ETV7 EZH2 FAM115C FAM65B FAS FBXL3 FBXL8 FBXO42 FCGR2A FERMT2 FES FFAR2 FGF18 FGF7 FGFR1OP2 FHL2 FILIP1 FJX1 FKBP11 FOSL1 FOXO1 FPR1 FPR2 FRRS1 FST FSTL1 FSTL3 FUZ FZD4 GABARAPL1 GABRR2 GAL GALNT2 GAS1 GBP5 GCH1 GK5 GLCE GLRX GLUL GMNN GNA12 GNAL GNB3 GNL3 GOLPH3 GPBP1L1 GPCPD1 GPR4 GPR64 GPRC5A GRAMD1A GREM2 GRK5 GTPBP2 H19 HAPLN3 HAS1 HDAC4 HECA HECTD2 HELZ2 HERC6 HIST1H4E HIST2H3D HK2 HLA-DOB HLA-L HNRNPH3 HNRNPLL HNRNPU-AS1 HOOK3 HP HTR1D HTRA3 IFI16 IFI27 IFI44 IFI44L IFITM1 IFITM2 IFITM3 IFNAR2 IFNGR1 IFNGR2 IGF1 IGF2BP2 IGFBP4 IGFBP5 IL10 IL12A IL13RA1 IL15RA IL17RA IL18BP IL18R1 IL18RAP IL1R1 IL1RL2 IL2RA IL4R IL6R IL7R ING1 INHBA INSIG1 INSR IRAK3 IRF2 IRF4 IRF7 IRF9 IRX3 ISG20 ITGA10 ITGA5 ITGB1 ITPR3 JAG1 JAK3 JOSD1 KCNE4 KCNT2 KIAA0247 KLF9 KLHL6 KLK7 KLK8 KLRD1 KSR1 LAP3 LATS2 LDHA LDLRAD3 LEPR LILRA5 LILRA6 LILRB3 LINC00473 LINC00887 LINC00959 LINC00960 LITAF LMNB1 LOC100127983 LOC100129617 LOC100506136 LOC100507387 LOC100996455 LOC154761 LOC257396 LOC646903 LONRF3 LOXL2 LRG1 LXN LYRM9 MAFG MAN1A1 MAOA MAP3K5 MAP3K6 MAP7D3 MAPK13 MAPK14 MAPK1IP1L MARCH3 MC1R MCC MCMBP MCTP2 MEFV MEG9 MGP MID1 MIDN MIEF2 MIR100HG MIR548AO MIR614 MLKL MOCOS MOV10 MROH6 MTHFD2 MTHFR MTMR12 MTUS1 MUC1 MUL1 MX1 MX2 MYD88 MYO1G NABP1 NADK NAMPT NAV1 NCAPH2 NCOA3 NCOA7 NDRG1 NDST2 NDUFAF7 NEDD4 NFATC4 NFE2L2 NFIL3 NFKBIZ NGF NICN1 NID1 NKX6-1 NMNAT3 NNMT NOD2 NPPC NRBF2 NRD1 NXPH4 OAS2 OAS3 OASL OSER1 OSMR OTOF OTOG OVCH1 OXCT2 P4HA1 PAPPA PAPPA2 PAPSS2 PARP14 PARP9 PCBP3 PCDHB11 PCDHGA5 PCK1 PCK2 PDE10A PDE3B PDE4D PDE7B PDE8B PDPN PFKFB3 PHF17 PHF21A PHLDA1 PHLDB2 PID1 PIF1 PIGA PIP5K1C PITPNC1 PLA2G2A PLAGL1 PLAUR PLCL2 PLEKHA2 PLSCR1 PLXNA4 PNRC1 POSTN POU6F2 PPAP2B PPARD PPIL4 PPP1CB PPP1R3C PROSER2 PRRX1 PSEN2 PSTPIP2 PTEN PTGER1 PTGIR PTP4A1 PTPN12 PTPN2 PTPN9 PTPRE PVR PVT1 RAB20 RAB39A RAP1A RARG RARRES1 RASSF5 RBBP7 RBM6 RBM7 RBMS1 RDH10 RELT RGS2 RHBDF2 RIOK1 RLF RND3 RNF122 RNF149 RNF19A RNF24 S100A14 S100P S1PR3 SAMSN1 SAP30 SAT1 SBNO2 SCO2 SDS SEC24D SECTM1 SERP1 SERPINB9 SESTD1 SGPP2 SH3BP5 SH3RF3 SHMT2 SIDT2 SIGLEC1 SIGLEC12 SIGLEC14 SIK1 SIRT1 SKIDA1 SLA SLC16A3 SLC16A4 SLC17A9 SLC1A1 SLC22A23 SLC25A37 SLC26A6 SLC2A3 SLC2A4 SLC35G2 SLC37A3 SLC4A4 SLC7A2 SLC7A8 SLC9B2 SLFN11 SLPI SMAD3 SMAD7 SMAP2 SMARCAL1 SMCHD1 SMOC1 SMOX SMYD4 SNAPC1 SNHG15 SNORA1 SNORA4 SNORA9 SNORD10 SNORD5 SNORD6 SNORD89 SNX9 SP110 SP140L SPAG4 SPIDR SPRY4 SPTLC3 SPTSSA SRGAP1 SRGN SRSF12 SRSF4 SRSF7 SRXN1 SSH1 SSH2 STARD8 STAT1 STAT3 STAT4 STEAP1 STEAP4 STK17B STK3 STOM STX11 SULT1B1 SULT1C4 SULT1E1 SURF4 SVEP1 SYCP2L SYTL4 TACC1 TAF1D TAP1 TAP2 TBC1D16 TBC1D8 TBX15 TCTA TEP1 TFPI TGFB2 THAP9-AS1 THBD THBS1 THBS2 THEMIS2 TIPARP TLL1 TLR2 TMEM100 TMEM25 TMEM255B TMEM43 TMEM45A TMOD1 TNFRSF11B TNFRSF1B TNFSF14 TPM4 TPST1 TRIB2 TRIM22 TRIM38 TRIOBP TSC22D1 TSC22D3 TSPYL2 TUBE1 TYMP TYSND1 UAP1 UBAP1 UBE2B UBXN2B UCK2 UIMC1 URB1 USB1 USP53 VCAN VPS37A WBP1L WDR41 WDR44 WWTR1 XAF1 XPNPEP2 YAF2 YBX3 ZBP1 ZCCHC10 ZFAND1 ZFHX3 ZNF215 ZNF286A ZNF385D ZNF394 ZNF516 ZNF727 ZNF815P ZRANB1*

**C_only1_M8:** *ACTN2 ANKRD37 ARG2 BHLHE40 BMP4 C2CD4A C6orf120 CSRNP1 DCUN1D3 EGR2 EGR3 ELL2 ELMSAN1 ERRFI1 F3 FBXW4P1 FRAT1 FRAT2 GADD45G IFRD1 IL18RAP IL6 ITPRIP KLHL21 LIF LOC285074 MAFF MAP3K8 MT1A MT2A NFKBID NR4A3 OVCA2 PHF13 PRR26 PXDC1 RGS16 RGS3 RPS14P3 SAT1 SDC4 SDS SLC20A1 TGIF1 TOB1 USP2 VPS37B XBP1 ZFAND5 ZWINT*

**SCH_only_M7:** *ACRC ACTRT3 AHSA1 ARHGAP17 AURKAPS1 BAG3 BMP4 C11orf84 CACYBP CCDC117 CCRN4L CHORDC1 DCAF4L1 DCUN1D3 DEDD2 DLC1 DNAJA1 DNAJA4 DNAJB1 DNAJB4 EIF1AD ELMSAN1 FAM46A FILIP1L FKBP4 HSP90AA1 HSP90AB1 HSP90B1 HSPA1A HSPA1B HSPA1L HSPA4L HSPA5 HSPA6 HSPA7 HSPD1 HSPH1 IER5 IL8 JMJD6 KLHL25 LOC648987 LOC729603 LOC90834 LRIG3 LSMEM1 MB21D1 NHLH1 NKRF NME2 PAPD5 PARD6G PARD6G-AS1 PNLDC1 PPM1D PPP1R15A PROZ PUS3 RUNX3 SCAF4 SERPINH1 SH3GL1P1 SIX4 SLC6A1-AS1 SNORD25 SNORD63 SPEN STIP1 TCP1 TOB2 TONSL TPM3P9 TRIM26 TRIM61 VGLL3 ZC3HAV1*

**S_R_M2:** *ABCB9 ACE2 ADAMTS4 ADH1B AKAP12 ALOX12 ALPL APCDD1 ARHGAP26 ARMCX2 ASCC1 AZI2 B4GALT1 B4GALT5 BACH2 BAZ1A BDKRB2 BMP1 BMP4 BMPER BZW1 C2orf47 C5AR2 CAPZA1 CBLB CBX4 CCL2 CCNL1 CD55 CDC37L1 CDK2 CHIC2 CLCF1 CNN3 CNOT8 CPD CRTC2 CSRP2BP CTGF CYP1B1 CYP26B1 DBF4 DDX21 DENR DET1 DFFB DSE DUSP4 DUSP6 ELL2 ELMSAN1 ENPEP EPCAM ERRFI1 ETS2 FAM65B FJX1 FKBP5 FMO2 FOSL1 FRMD8 FZD4 GABARAPL1 GAL GAS2L1 GLUL GNL2 GPR88 GTF2B HGSNAT HSD11B1 HTRA3 ID1 IFNGR1 IGFBP3 IL15RA IL18R1 IL1R1 IL4R IL6 IL6ST IRAK3 ITFG2 ITPKC JHDM1D KBTBD3 KCNT2 KIT KLF15 KLF3 KLF9 LAMA2 LCNL1 LIF LINC00473 LOC100507217 LRRC57 LXN MAP1LC3B MAP3K13 MAP3K6 MAPK1IP1L MEDAG METTL25 MMP19 MOCOS MORF4L2 MOV10L1 MPZL2 MT1A MT1X MTHFD1L MYBPH NAA16 NAMPT NCOA7 NDUFAF4 NEDD9 NEK3 NFKBIZ NUTM2A-AS1 OSMR P2RY14 PABPC4 PAPSS2 PDE7A PDE7B PDGFRA PDK4 PITPNC1 PLCL2 PNRC1 POLR3C POSTN PPARD PPP1CB PPP1R3G PRKAB1 PTP4A2 PTPDC1 PTPRE PXN QRFPR RAB21 RAD51D RARRES1 RBM15 RBM44 RBMS1 RBP1 RHOU RND3 RQCD1 RSRC2 RWDD2B S100A16 SAT1 SBNO2 SCML1 SDCBP SEMA4F SERPINB1 SERPINB9 SH3PXD2B SLC1A1 SLC22A23 SLC7A2 SMAP2 SMOC1 SMOX SNHG3 SNORA45 SNORD31 SNORD89 SNRNP27 SOCS3 SP6 SPAG5-AS1 SPRY2 SPRY4 SRGN SSH2 STAT3 TACC1 TACR1 TAF1D TBX15 TGFBR2 TGIF1 THAP9-AS1 THBS1 TLCD2 TMEM165 TMOD1 TNFRSF10D TNFRSF1A TNFRSF6B TNKS1BP1 TRA2B TRIB2 TUBB6 TXNDC15 TXNIP UBL4A UGGT2 URB1 USH1C USP53 WDR41 YPEL5 ZFAND5 ZNF267 ZNF394 ZNF595 ZNF700 ZNF805 ZNF879 ZSWIM4*

**C_R_only_M10:** *ALDOA C19orf77 EDN2 FAM195A FLJ45513 GPS2 HMOX1 HSBP1L1 IER3 KCNG2 KISS1R KRT6A LPCAT1 MIR210 MIR210HG MTFP1 MTRNR2L8 MTRNR2L9 PQLC1 PRR5 SCGB1A1 SFTPA1 SFTPA2 SFTPB SFTPC SLC14A2 SOX1 SPC25 STAC2 TMEM191A*

**S_R_only_M1:** *AIG1 ARHGAP28 ASB9 B2M BST2 C8orf34 CHODL CLEC9A CMBL CPZ CTLA4 CXCL10 CXCL11 DDX58 DDX60 DDX60L DHX58 DPH6-AS1 DTX3L EGFL6 EPHB2 EPSTI1 EYS FMO1 FOXP2 FRMD6 FZD10-AS1 GAPT GBP1 GBP1P1 GPHA2 HCG23 HERC6 HESX1 HLA-A HLA-B HLA-C HLA-DOA HLA-DQA2 HLA-DQB2 HMGCLL1 HMGN1 HPGDS HSH2D HYMAI IFI27 IFI35 IFI44 IFI44L IFI6 IFIH1 IFIT1 IFIT2 IFIT3 IRF4 IRF9 ISG15 KANK4 KCNV1 KIAA1210 LGALS3BP LOC149684 LRRC17 LY6E MARCKS MDK MX1 MX2 NME8 NT5E NXPH2 OAS1 OAS2 OAS3 OASL PARP12 PARP14 PARP9 PCOLCE-AS1 PDGFRL PGRMC1 PLA2G7 PPP5D1 PRRT3-AS1 PSMB9 RARRES3 RGS1 RTP4 SAMD9 SAMD9L SCN2B SCN4B SLC26A2 SLC30A3 SP110 SST STAT1 TMEM140 TRIM21 TULP1 TXLNB UBE2L6 USP18 VMO1 XAF1 ZBP1*

**S_R_only_M7:** *APOA1 C12orf75 C1QTNF2 C1QTNF7 C20orf166-AS1 CCDC3 CXCL12 EBF1 ESR1 FABP3 FOXF2 GPR124 HRC ITM2A KLF2 LAMA4 LINC00987 LSMEM2 MFAP4 MRGPRF MUSTN1 MYOZ1 NRIP2 PRICKLE1 RAMP2 RSPO3 SFRP2 SGCA SLC8A1 TMC4*

**S_R_only_M12:** *ACTN2 ARC ARG2 BMP4 C3orf35 CCNB1IP1 CD160 CEP95 ENDOU EVA1C FAM41C HAMP IL1RL1 KCNE2 LINC00312 LYPD3 MAP3K8 MIR22HG MIS18A MT1E MT1M PHC2 PIGA PMAIP1 PSMD12 PSMD6-AS2 S100P SDC4 SDS SFN SLC2A5 SOD2 TBC1D8 TRIB1 TRMT11 YPEL5 ZFAND5 ZNF256*

**S_R_only_M18 :** *ABCA7 ACKR4 ANKRD33B ANO9 AQP3 ATG16L2 ATP8B3 BAIAP2L1 BMF BMP8B C14orf37 C2orf71 C9orf43 CABLES1 CAPN12 CATSPERG CCBL1 CCNE1 CDH24 CES3 CHRNA5 CHST2 CIB2 CLDN10 CMTM8 CNGB3 COL11A1 COL6A4P2 E2F1 EPS8L1 ERVMER34-1 EZH2 FCHSD1 FGF5 FGFR4 FLJ23867 GLA GPC2 GPR160 GPR173 HGFAC HLA-L HMGA1 HMGCS2 IFRD1 IGSF1 IMPA2 INPP5J ITGA2B KCNJ11 KIAA1984 KIFC2 KNOP1 KREMEN1 LAPTM4B LAYN LINC00950 LINC01010 LOC100506100 LOC729970 LRRC3 LTK MAP1LC3C MDGA1 MEX3A MMEL1 MREG NCMAP NPAS1 OPHN1 PALM3 PARD6G PDZD7 PELI3 PLA2G3 PPM1J PPP1R1A PRKCSH PRRT4 PRSS8 PTGES RAC3 RADIL RAET1G RASGEF1A RASL10B REEP6 RELL2 RHBDL3 RNF128 SCN4A SERINC2 SFXN2 SH2D3A SHISA9 SLC2A4 SLC45A1 SLC4A11 SLC9A4 SSPO SUSD3 TG TLL2 TMEM108 TMEM56 TMEM63C TMEM74B TNFAIP8L1 TRIM16 TRIM16L TTC9 TTF2 USH2A WIF1 WNT2B XPR1*

**S_R_only_M20:** *ABCG4 ADAM11 ADAM23 AGPAT6 AMDHD1 ASPHD1 B3GALT2 BAI2 CACNG7 CELSR3 CHAF1B CHST1 CLSTN3 CORO2A EPB41L1 FLJ22184 FNDC4 GAL3ST3 KCNK12 KIAA0513 KIF1A KIF21B KIF3C LIMK1 LMTK3 LOC100130705 LOC441666 LPHN1 MAGEE2 MAP3K10 MEGF11 NUDT10 NUDT11 NYAP1 PAK7 PODXL2 POPDC3 PSD3 RAB15 RAB3B RLTPR RTN3 SBK1 SCN3A SH2D5 SLC9A7 SPATS2 TMCC2 TMEM158 TNNT1 TTBK1 TTC39A*

**S_Co_M16*:*** *ABCA1 ADAMTS4 AKAP12 ALOX15B ALOX5 ALPL ANKRD22 APCDD1 APOLD1 AQP9 ARF6 ARHGAP6 ARHGEF19 ATAD2 B4GALT5 BAZ1A BCL2A1 BCL2L11 BCL3 BCL6 BDKRB2 BMP1 BMPER BTG1 BTG3 BZW1 C17orf96 C19orf59 C1RL C1orf112 C1orf162 C5AR2 CAPZA1 CARD6 CASP4 CBLB CCDC71L CCNL1 CCR7 CD163 CD1D CD209 CD300A CD300E CD38 CD55 CD72 CDC37L1 CDC42SE1 CDC6 CEACAM1 CEBPD CHI3L2 CLEC4E CLK1 CR1 CRISPLD2 CSF3R CTPS1 CYP1B1 DARC DBF4 DDIT4 DDX21 DENND3 DIO3 DNAJC12 DPF3 DRAM1 DSE ECEL1 EDN1 EFNA1 ELL ELL2 EMR2 ENPEP EPB41L3 ETS2 EVA1C FAM115A FAM115C FAM65B FBXO32 FCGR2A FCGR3B FES FFAR2 FGF18 FGF7 FJX1 FKBP11 FKBP5 FOSL1 FPR1 FRMD8 FSTL1 FSTL3 GABARAPL1 GAL GLCE GLRX GLUL GMNN GNA12 GNAL GNL2 GNL3 GPR97 GRAMD1A GREM2 H19 HAS1 HCLS1 HDAC7 HK2 HP HTRA3 IFITM1 IFITM2 IFITM3 IFNGR1 IFNGR2 IGF1 IGF2BP2 IGFBP4 IL12A IL15RA IL17RA IL18R1 IL18RAP IL1R1 IL1R2 IL2RA IL4R IL6ST INHBB IRAK3 IRF2 ISG20 ITGA10 ITPKC JAK3 KCNE4 KCNJ15 KCNK15 KCNT2 KLF9 KLHL6 KLRD1 KRT18 LDHA LDLRAD3 LILRA5 LILRA6 LILRB2 LILRB3 LINC00473 LINC00887 LMNB1 LOC100506115 LOC154761 LOC646762 LPCAT3 LRG1 LXN MAN1A1 MAP3K6 MAPK13 MAPK1IP1L MARCH3 MCTP2 MEDAG MEFV MEG9 MGP MLKL MLLT11 MMP19 MOCOS MOV10 MROH6 MSR1 MT1A MT1X MT2A MTHFD1L MUC1 MXD1 MYC MYD88 MYO1G NABP1 NAMPT NAV1 NCOA7 NFATC4 NFE2L2 NFKBIZ NGF NICN1 NID1 NNMT NOD1 NOD2 NOLC1 NPPC OSMR OXTR P4HA1 PABPC4 PAPSS2 PCDHB4 PCK2 PCTP PDE7B PDE8B PDLIM1 PHC2 PHLDB2 PIF1 PITPNC1 PLA2G2A PLAGL1 PLCL2 PLEKHA2 PLEKHA4 PLIN2 PLSCR1 PLXNA4 PNRC1 POSTN PPARD PPP1CB PPP1R3C PROSER2 PSTPIP2 PTGER1 PTPDC1 PTPN2 PTPRE PXMP4 PXN PYGL RAB20 RAB21 RARRES1 RBMS1 RELA RGS2 RHBDF2 RHOU RND3 RNF125 RNF149 RNF24 RPF2 RPS6KA3 RUNX1 S100A12 S100A8 S100A9 S100P S1PR3 SAA1 SAMSN1 SAP30 SAT1 SBNO2 SDCBP SDS SEC24D SECTM1 SEH1L SELL SERPINA1 SERPINA3 SERPINB1 SERPINB8 SERPINB9 SHC1 SHMT2 SIGLEC12 SIGLEC14 SIGLEC7 SIGLEC9 SIRPB2 SIRT1 SLA SLC11A1 SLC16A3 SLC17A9 SLC1A1 SLC22A23 SLC26A6 SLC43A3 SLC7A2 SLED1 SLPI SMAD3 SMAP2 SNORD10 SNORD89 SOCS3 SPAG4 SPIDR SPRY4 SPTLC3 SRGAP1 SRGN SSH2 STARD8 STAT3 STEAP1 STEAP4 SULT1B1 SULT1C4 SULT1E1 SYCP2L TACC1 TBC1D16 TBC1D8 TBX15 THAP9-AS1 THBD THBS1 TIMP1 TLR2 TLR8 TMBIM1 TMEM100 TMEM255B TMEM71 TMOD1 TNFRSF1A TNFRSF1B TNFSF14 TNKS1BP1 TPM4 TPST1 TRIB2 TRIOBP TSC22D1 USH1C USP53 VNN2 WDR41 WTAP XPNPEP2 YAF2 YBX3 ZNF516*

**LPS_3h_M2:** *0610009F02Rik 0610009H04Rik 0610039K22Rik 0610039P13Rik 0710001C05Rik 1110001A05Rik 1110001K21Rik 1110002B05Rik 1110003P22Rik 1110004B15Rik 1110006I15Rik 1110018J23Rik 1110019L22Rik 1110025F24Rik 1110028E10Rik 1110030J09Rik 1110032E23Rik 1110067D22Rik 1190002H23Rik 1190005I06Rik 1200002N14Rik 1200003I07Rik 1200009O22Rik 1200013B22Rik 1300002F13Rik 1300017K07Rik 1300018P11Rik 1500003O03Rik 1500004A08Rik 1500032H18Rik 1700010L19Rik 1700023A16Rik 1810037K07Rik 1810049K24Rik 1810054O13Rik 2010301N04Rik 2210021A15Rik 2310002A05Rik 2310003L06Rik 2310004N11Rik 2310007B03Rik 2310046K01Rik 2310046K10Rik 2310057H16Rik 2310057J16Rik 2310058J06Rik 2410015B03Rik 2410076I21Rik 2410118P20Rik 2410141M05Rik 2510004L01Rik 2600005C20Rik 2610024E20Rik 2610510D13Rik 2610529H08Rik 2810046M22Rik 2810049G06Rik 2810452K22Rik 2810457M08Rik 2810474O19Rik 2900016G23Rik 2900057K09Rik 2900090M10Rik 3110032G18Rik 3110043J09Rik 3110070M22Rik 3322402L07Rik 4432411E13Rik 4631426J05Rik 4632417D23 4732418C07Rik 4732429I09Rik 4833401D15Rik 4921509B22Rik 4921513O20Rik 4921517D22Rik 4922501C03Rik 4930402E16Rik 4930422J18Rik 4930486L24Rik 4930488P06Rik 4930519N16Rik 4930525K21Rik 4930573I19Rik 4930599N23Rik 4931432E15Rik 4933405A16Rik 4933417E01Rik 4933429I20Rik 4933430F08Rik 4933434I06Rik 4933436E20Rik 5031414D18Rik 5133401N09Rik 5730410E15Rik 5730411O18Rik 5730438N18Rik 5730509K17Rik 5730537H01Rik 5830484A20Rik 5830493J20Rik 5930434B04Rik 6030423D04Rik 6030440P17Rik 6330406I15Rik 6330415M09Rik 6330442E10Rik 6330548G22Rik 6430526O11Rik 6430548M08Rik 6820443O06Rik 8030498B09Rik 8430408G22Rik 9030623N16Rik 9030625A04Rik 9130213B05Rik 9130218O11Rik 9130416B15 9230105E10Rik 9330140K16Rik 9430004M15 9430038I01Rik 9530080O11Rik 9830147J24Rik 9830147P19Rik 9930017A07Rik 9930021J17Rik 9930022D16Rik 9930111J21Rik A030013D21 A130052D22 A230046K03Rik A230053A07Rik A2m A330043L12 A330066M24Rik A430005L14Rik A430005L14Rik A430005L14Rik A430093F15Rik A630077B13Rik A730069N07Rik A930008G19Rik A930021H16Rik A930029B02Rik AA175286 AA589507 Abat Abi3 Acadm Acat3 Ache Acin1 Acvrl1 Acy3 Adam7 Adamts1 Adamts1 Adamts4 Adamtsl1 Adh1 Adora2b Adprhl2 Adprtl3 Adra2c AI115600 AI428936 AI481100 AI481105 AI481214 AI481214 AI646023 AI929863 Ak1 Akap12 Aldh3a1 Aldoa-ps2 Alox12 Als2cr2 Ampd2 Anp32a Anp32b Anpep Aoc3 Aox3 Aqp1 Arhgap4 Arhgap9 Arhgef19 Arhgef6 Arid5a Arntl Arrdc3 Asph Asph Atf7ip Atp10d Atp2a2 Atp5a1 AV312086 AW456874 AW491445 Axud1 AY026312 AY078069 B130017I01Rik B230342M21Rik B3galt1 B930018B01 Bach2 Bambi Batf BC003236 BC003277 BC003281 BC003324 BC004022 BC004728 BC011209 BC019206 BC020025 BC021608 BC022765 BC023741 BC023829 BC023957 BC030863 BC031575 BC036333 BC038156 BC049975 BC060267 BC061259 Bcar3 Bcas2 Bcl10 Bcl6 Bcor Birc2 Birc5 Bmp4 Bmp6 Bmpr1a Bmx Brca2 Bst2 Btg1 Btg1 C1qg C1qr1 C1rl C2 C3 C330016O10Rik C430004E15Rik C430010P07Rik C630023L15Rik C730036B14Rik C730049P21 C80913 Cables1 Cacna1f Cacna1h Caln1 Calu Car13 Car4 Card10 Caskin2 Casp4 Casp4 Catnbip1 Cav1 Cbfa2t3h Cbln1 Ccl11 Ccl12 Ccl19 Ccl2 Ccl4 Ccl4 Ccl5 Ccl7 Ccl9 Ccng1 Ccng1 Ccr4 Ccrl2 Ccrn4l Ccrn4l Cd14 Cd47 Cd69 Cd83 Cd86 Cd86 Cdc42ep2 Cdc6 Cdca1 Cdh11 Cdk8 Cdkn1a Cdkn1a Cds2 Ceacam2 Cebpb Celsr2 Celsr3 Ch25h Chi3l1 Cirbp Cish Cklfsf3 Clcn1 Clcn3 Cldn11 Cldn3 Cldn5 Clic4 Clic4 Clic4 Clic4 Cml4 Cnn2 Cobll1 Cobll1 Col27a1 Col4a1 Copeb Cpt1b Cpt2 Crem Crip1 Cryz Cryzl1 Csda Csda Csf1 Csf1r Csf3 Csf3 Csnk1d Csnk1g1 Ctbs Ctps Ctps Ctrl Cx3cl1 Cx3cr1 Cxcl1 Cxcl10 Cxcl12 Cxcl13 Cxcl16 Cxcl2 Cxcl9 Cygb Cyp1b1 Cyp2s1 Cyp4f15 D030015G18Rik D11Ertd498e D11Ertd498e D11Ertd759e D130027M04Rik D13Bwg1146e D14Abb1e D14Ertd668e D17H6S56E-5 D19Ertd386e D2Wsu81e D3Jfr1 D4Bwg0951e D5Ertd593e D630045E04Rik D7Bwg0611e D930030O05Rik Daxx Dbp Ddit4l Ddx25 Ddx5 Ddx58 Defcr-rs10 Dfy Dgat2 Dhrs7 Dio2 Dmn Dmrt3 Dnajb5 Dnmt3l Dock7 Dok3 Dph2l1 Dpysl3 Dscr1 Dscr1 Dscr6 Dsg2 Dst Dusp1 E030010A14 E030024M05Rik E130102H24Rik E130113K08Rik E130203B14Rik E2f6 E2f6 E2f6 Eaf1 Ebi3 Eda Edaradd Edg1 Edn1 Edn2 Efnb1 Egfr Egr1 Eif2b3 Eif4ebp1 Ell Eltd1 Emid2 Emilin1 Eng Enpp6 Epb4.1l4a Erbb2 Esm1 Esrra Ets2 Ewsh Exoc7 Ext2 F3 Fabp6 Fasn Fbf1 Fbn1 Fbxl20 Fbxo24 Fbxo25 Fbxo27 Fbxw17 Fcgr2b Fgd3 Fgd5 Fgf18 Fgfrl1 Fkbp5 Flot1 Flrt2 Fmnl2 Fmnl3 Fndc3 Fos Fosl2 Foxp4 Frmpd1 Frzb Fstl1 Fzd10 G0s2 G1p2 G3bp Gabpb2 Gad1 Gadd45a Gadd45g Galnt3 Gata2 Gbp4 Gbp5 Gcg Gch1 Gcn5l2 Gcnt2 Gdf15 Gfpt2 Gja1 Gja4 Gjb2 Gjb6 Gm644 Gna11 Gng13 Gp38 Gpd1 Gpd2 Gpha2 Gpihbp1 Gpihbp1 Gpr73 Gpr84 Gpr88 Gprc5c Gpx3 Gria3 Gsn Gucy1b3 H2-Bf H2-K1 H2-L H2-L H2-Q2 H2-Q6 H2-Q8 H2-T17 H2-T22 H2-T9 H60 H6pd Haao Has1 Hba-a1 Hccs Hdac11 Helb Hes1 Hey1 Hic1 Hist1h2ah Hist1h2ai Hivep1 Hk2 Hnrpk Hnrpr Hook1 Hp Hpca Hpgd Hrbl Htr1d Icam1 Idb2 Ier3 Ifi1 Ifi205 Ifi47 Ifit2 Ifit3 Ifit3 Ifit3 Ifitm1 Ifitm3 Ifnar2 Ifngr2 Igfbp4 Igtp Il11 Il13 Il15 Il18bp Il1b Il28ra Il4i1 Il6 Il6st Ilvbl Ing3 Inhbb Irak3 Irf1 Irf1 Irf1 Irf2 Irf7 Irs3 Isg20 Isgf3g Isgf3g Isl1 Itga10 Itga8 Itm2a Jam3 Jun Junb Jup Kcne2 Kcnf1 Kcnj15 Kcnk3 Kctd2 Kdt1 Kif2a Klf2 Klhl6 Kmo Kpna4 Krt1-12 Krt1-23 Krt2-8 Ktn1 L259 L3mbtl3 Lactb Lcn2 Ldb2 Lgi1 Lgtn Lip1 Litaf Lman1l LOC209387 LOC213233 LOC239447 LOC260408 LOC330599 LOC384419 LOC56628 Lox Loxl4 Lrig1 Lrig3 Lrp4 Lrrn2 Lrrn3 Luzp1 Ly6a Ly96 Lypla1 M6prbp1 Mad2l2 Madcam1 Maff Mafg Maml1 Map2k3 Map3k1 Map3k14 Map3k3 Map3k5 Map3k6 Map3k8 Mapk11 Mapk14 Mapk4 Mapk8 Marveld1 Mat2a Mc3r Mcf2l Mcoln2 Mdm2 Mef2c Meox1 Mertk Mfap2 Mfap3 Mfng Mgam Mgat3 MGC6357 Mgll Mgll Mical3 Mid1 Mid1ip1 Miz1 Mlp Mlp Mlr2 Mmp12 Mmp13 Mmrn2 Mospd1 Mpp7 Mpra Mpz Mrvi1 Ms4a10 Ms4a6d Msc Msx1 Mt1 Mt2 Mt4 Mtap1b Muc1 Muc1 Muc1 Mucdhl Mx1 Mx1 Mx2 Mybph Myd88 Myl9 Myl9 Myo10 Myo1e n/a n/a n/a n/a n/a Nav1 Nbr1 Ncald Ncdn Ndufb4 Nedd9 Negr1 Nek11 Net1 Neurl Nfatc1 Nfe2l2 Nfil3 Nfkb1 Nfkb2 Nfkbia Nfkbie Nfkbie Nfkbiz Ngfb Nid1 Nktr Nme7 Nol5 Nos3 Notch4 Npas2 Nr1d1 Nr2f1 Nr2f6 Nrp Nrp Nudt7 Numbl Nup50 Nupr1 Oas1b Oas1g Oas1g Oas1g Oas2 Oasl1 Oasl1 Oasl2 Ocil Ogfr Olfr1209 Olfr44 Olfr592 Olfr836 Olfr920 Opn1mw Osmr Oxt P2rx5 P2ry2 Pacsin3 Paip2 Palm Parp14 Pawr Pcdh7 Pcdhgb6 Pdcd1lg1 Pde8a Pdgfb Pdgfrb Pdk4 Pdlim1 Pdlim2 Pdlim2 Pdlim2 Pdzk3 Pecam1 Peli1 Pfkm Pfn1 Phf11 Phf17 Phka1 Phlda1 Pias3 Piga Pim3 Pkia Plac8 Plagl1 Plagl2 Plaur Plcb4 Plcd3 Plek2 Plekhf1 Plekhh1 Plk3 Plscr1 Plvap Plxna2 Pml Pnp Pnp Pnpla2 Podxl Poln Por Ppargc1b Ppp1r14b Ppp1r15b Ppp1r3b Prc1 Prdx2 Prg4 Prh1 Prickle1 Prkr Prp2 Prrx2 Prss34 Psg23 Psmb10 Psmb8 Psmb9 Psmd9 Psme2b Ptger1 Ptp4a3 Ptpn11 Ptprb Ptx3 Pvalb Rab38 Rab3a Rabgap1l Rai14 Ralb Rarb Rarg Rasa3 Rasip1 Rassf1 Rbl1 Rbm10 Rbm5 Rbm6 Rbm6 Rcor1 Rela Relb Retnlg Rgs10 Rgs12 Rgs16 Rgs19ip1 Rgs4 Rgs9 Rhbdl6 Rhob Rhoe Rhoj Rhot2 Rhou Ripk1 Ripk2 Robo4 Rock2 Rorc Rps6ka2 Rras2 Rtn4rl1 Rxra S100a10 S100a8 S100a9 S3-12 Saa1 Saa2 Saa3 Samhd1 Sat1 Sav1 Sca10 Scara3 Scarf2 Scnn1b Sct Scx Sele Selp Sema4a Serpina3c Serpina3g Serpina3n Serpinb6b Sertad4 Sfrs10 Sfxn4 Sh3bp2 Sh3bp4 Sh3yl1 Siah1b Siat4a Slc1a1 Slc1a3 Slc23a2 Slc25a18 Slc26a7 Slc2a3 Slc2a6 Slc30a4 Slc31a2 Slc35a5 Slc35d1 Slc41a2 Slc43a3 Slc5a5 Slc6a6 Slc7a11 Slc7a5 Slc9a3r2 Slc9a3r2 Slco2b1 Slfn1 Slfn2 Slit1 Smad3 Sncg Sntb2 Snx10 Socs1 Socs2 Socs3 Sox18 Sp6 Spa17 Sphk1 Spnb3 Stag1 Stard8 Stat1 Stat1 Stat2 Stat3 Stat3 Stc1 Stk19 Stk19 Stk2 Stk35 Stx11 Sult1a1 Surf2 Syngr2 Syt4 T2bp Tac1 Tap1 Tap2 Tbx22 Tbxa2r Tead4 Tef Temt Tex2 Tgfb1i4 Tgfbi Tgfbi Tgfbr2 Tgif Tgm2 Tgtp Thbd Thbs1 Thbs1 Thra Thy28 Tifp39 Tigd3 Timp1 Tisp78 Tle1 Tlr2 Tmc7 Tmem2 Tmem34 Tnf Tnfaip2 Tnfaip3 Tnfaip8 Tnfrsf19l Tnfrsf1a Tnfrsf1a Tnfrsf4 Tnfrsf5 Tnfrsf5 Tnfrsf6 Tnip1 Tob1 Tor3a Tor3a Tpm4 Tpm4 Traf1 Traf4 Traf4 Trex1 Trib2 Trim25 Trim26 Trim30 Trim34 Trim35 Trim56 Trp53 Trpv2 Trpv4 Txnl2 Tyki Ube1l Ube2l6 Ube2l6 Ubtf Ugcg Uhrf2 Ulk1 Upp1 Upp1 Usp12 Usp18 Usp53 V2r6 Vars2 Vcam1 Vdr Vdrip Vegfa Viaat Wfdc1 Wnt5a Wnt5b Wsb1 Xbp1 Xdh Xrcc4 Yeats4 Zbtb5 Zc3hav1 Zfp1 Zfp275 Zfp36l1 Zfp46 Zfpm1 Znrf1 Zswim4*
