# Supplementary material for: Transcriptome sequencing of the choroid plexus in schizophrenia
Source: Transl Psychiatry. 2016 Nov 29;6(11):e964–. doi: 10.1038/tp.2016.229 (PMC5290353; doi:10.1038/tp.2016.229)
Supplement: Supplementary Table 31 [file tp2016229x5.docx]

**Supplementary Table S31**. Top 5000 probes that were selected from data of mice 3 hrs post LPS treatment and controls and corresponding gene symbols

*2690537 (CXCL1)*

*6510390 (SAA3)*

*2510112 (LCN2)*

*430047 (CCL4)*

*2650519 (CCL7)*

*2120619 (2510004L01RIK)*

*1770347 (CCL11)*

*5340400 (SERPINA3N)*

*3170093 (G1P2)*

*5550563 (SOCS3)*

*6860286 (MT2)*

*4760019 (CCL2)*

*6400253 (TIMP1)*

*870309 (PTX3)*

*2450278 (IFIT3)*

*6980138 (ICAM1)*

*6660707 (CSF3)*

*3840180 (PDCD1LG1)*

*4810735 (IFIT2)*

*580746 (A630077B13RIK)*

*6200113 (IFIT3)*

*5860487 (PARP14)*

*2030373 (MX2)*

*5270687 (OASL1)*

*2760019 (IFI205)*

*3710397 (CCL5)*

*6860528 (PFKFB3)*

*6520471 (GBP4)*

*2810138 (TGTP)*

*2450408 (CXCL10)*

*2900142 (TNFAIP3)*

*2690451 (9830147J24RIK)*

*4230048 (JUNB)*

*2370092 (BCL2A1B)*

*2480014 (GBP5)*

*50368 (CCL4)*

*70112 (S100A8)*

*5420372 (LST1)*

*4010110 (USP18)*

*6420309 (AKAP12)*

*2810092 (CCL3)*

*6980577 (IRF1)*

*5700138 (BCL2A1D)*

*870044 (FOSL2)*

*6040193 (SELP)*

*6110148 (IFI1)*

*5390520 (SAA1)*

*4200270 (DBP)*

*360435 (HBA-A1)*

*1400048 (RELB)*

*1990519 (OSMR)*

*5860156 (PRG4)*

*510471 (MMP13)*

*2190563 (2310057H16RIK)*

*1090438 (TYKI)*

*430435 (ADAMTS4)*

*5570600 (CD14)*

*6590184 (STX11)*

*940100 (BCL6)*

*5050162 (TLR2)*

*1570673 (CXCL9)*

*6110440 (AI481100)*

*4210167 (CH25H)*

*2940487 (D11ERTD759E)*

*2450075 (DDX58)*

*630706 (PFKFB3)*

*580390 (NFKBIE)*

*4730601 (AA175286)*

*6400390 (MAP3K6)*

*6940671 (LITAF)*

*780091 (RGS16)*

*1850315 (FOS)*

*5860021 (IER3)*

*670364 (GCH1)*

*2760471 (TNFRSF5)*

*2570056 (IGTP)*

*2970019 (CEBPB)*

*4050102 (IRAK3)*

*2230348 (SCT)*

*130332 (AIF1)*

*130717 (PODXL)*

*5890082 (MCOLN2)*

*1940458 (OAS1G)*

*610398 (CXCL2)*

*6420671 (OCIL)*

*2190438 (FPR-RS2)*

*2640722 (HK2)*

*6110040 (TNFRSF6)*

*5360452 (TGM2)*

*2940286 (MAP3K8)*

*2690121 (BC019206)*

*1050168 (BC023741)*

*5570450 (PVALB)*

*7000072 (AI481214)*

*5390538 (NEURL)*

*5910242 (CD52)*

*4760471 (CCRL2)*

*3450040 (TREX1)*

*3780519 (STAT2)*

*3850358 (UPP1)*

*1580010 (MADCAM1)*

*3450273 (1300002F13RIK)*

*4850170 (TRIM30)*

*5860047 (CASP4)*

*4070377 (NFIL3)*

*5220692 (BC049975)*

*870239 (PRKR)*

*2510279 (TNIP1)*

*5080487 (CCL19)*

*2970450 (NFKBIZ)*

*3360088 (SLC9A3R2)*

*3060673 (IFI47)*

*2470113 (SPHK1)*

*3120400 (CSF1)*

*60204 (MSR2)*

*2340369 (CD72)*

*1230025 (CLIC4)*

*70438 (TNFAIP2)*

*3940050 (DFY)*

*2640368 (SIAT4A)*

*5220053 (PTPRB)*

*7000408 (BCL2A1A)*

*4760168 (ASB2)*

*2970092 (ADAMTSL1)*

*2810452 (BC003281)*

*6220039 (DSCR1)*

*1780047 (STARD8)*

*6980471 (PSMB9)*

*3990524 (1300017K07RIK)*

*610600 (SERPINA3G)*

*2690301 (MX1)*

*6290632 (RIPK2)*

*2680092 (INHBB)*

*2340152 (IRF1)*

*2450280 (OAS1G)*

*1500288 (IFNGR2)*

*1740132 (CTPS)*

*7100142 (ISGF3G)*

*4610347 (EGR1)*

*1410594 (ESM1)*

*1230079 (LGALS9)*

*1690577 (CX3CR1)*

*2650301 (PLSCR1)*

*1500592 (EMR1)*

*2640364 (IL1B)*

*1570605 (IRF7)*

*6510204 (STAT1)*

*3130092 (PRKCB)*

*5860463 (ADAMTS1)*

*2230093 (LY86)*

*2940180 (F3)*

*360161 (STC1)*

*4050088 (CDKN1A)*

*2690441 (OASL2)*

*3120114 (SLFN2)*

*450609 (SLC2A6)*

*3520102 (PTX3)*

*5890184 (ERDR1)*

*4850577 (MAFF)*

*510278 (CXCL16)*

*5550020 (FCER1G)*

*5340372 (LOX)*

*2900184 (CAR13)*

*3290114 (PPARGC1B)*

*5550372 (LAPTM5)*

*4760450 (TRIM34)*

*1570152 (NFKBIA)*

*780497 (STAT1)*

*5910280 (PLAUR)*

*2640181 (LGALS9)*

*6200133 (BTG1)*

*2370563 (SAMSN1)*

*1580021 (RASIP1)*

*2650347 (AI115600)*

*6400706 (CDKN1A)*

*2680427 (UBE2L6)*

*1580541 (RAC2)*

*2810347 (TAP2)*

*5050551 (ADPN)*

*6130435 (P2RX5)*

*3190180 (CLIC4)*

*6290193 (LRMP)*

*2940301 (D14ERTD668E)*

*1230408 (OAS2)*

*3190131 (ISGF3G)*

*770725 (HMGCS2)*

*2810059 (FCRL3)*

*2900113 (FSTL1)*

*6510717 (CLDN5)*

*6450132 (PLXNA2)*

*5910286 (DUSP6)*

*5220369 (MBP)*

*3170427 (ADH1)*

*4200735 (BTG1)*

*3940100 (UPP1)*

*6660041 (CREM)*

*5900458 (D130027M04RIK)*

*3360138 (PIM3)*

*2570722 (GNG13)*

*1410168 (FGF18)*

*6840333 (AI481105)*

*2850138 (SLC7A11)*

*2060465 (RASA3)*

*2230193 (CSF3)*

*870551 (E130203B14RIK)*

*2340722 (MS4A6D)*

*2760463 (5830484A20RIK)*

*2630142 (CYP26B1)*

*770746 (NR1D1)*

*3990440 (BCL3)*

*4730017 (GDF15)*

*3440050 (1810049K24RIK)*

*3990692 (1500032H18RIK)*

*1500309 (RHOB)*

*70288 (BLNK)*

*3290368 (CLIC4)*

*2900341 (N/A)*

*6550075 (IGH-6)*

*3190020 (CORO1A)*

*360400 (EMID2)*

*5360400 (PLEKHF1)*

*4850139 (ATP10D)*

*5420358 (NFKB1)*

*6100736 (8430408G22RIK)*

*460040 (STAT3)*

*5220731 (GJA1)*

*1230053 (AXUD1)*

*4560397 (NDRL)*

*4070079 (D930048N14RIK)*

*1090520 (4930519N16RIK)*

*870142 (MAPK4)*

*4050047 (TAP1)*

*4570546 (HEY1)*

*6380670 (N/A)*

*510358 (N/A)*

*4570440 (D4BWG0951E)*

*3840082 (PML)*

*6760390 (BATF)*

*6040181 (TGFB1I4)*

*5690600 (1200013B22RIK)*

*670170 (EGFL7)*

*3170463 (ARNTL)*

*2470706 (RHOU)*

*6450050 (LOC209387)*

*50438 (CCRN4L)*

*6760593 (ANGPTL4)*

*4560273 (RNF125)*

*5340300 (EBI3)*

*4280136 (OLFR995)*

*1400736 (A630039F14RIK)*

*4610725 (CCL9)*

*2680136 (SPON2)*

*2320670 (NFKB2)*

*6860253 (ADORA2B)*

*2350338 (SAMHD1)*

*5420524 (EGFR)*

*2470048 (HAS1)*

*2480537 (TRIM25)*

*3710300 (SLAMF9)*

*1740372 (C3)*

*2650133 (NCF2)*

*2320086 (SOX18)*

*4780056 (CSDA)*

*6020593 (9230105E10RIK)*

*5670358 (CBLN1)*

*4570497 (OASL1)*

*1450577 (SLC31A2)*

*3140632 (LRIG3)*

*110010 (BC022765)*

*6450427 (KLHL6)*

*3450592 (IRF1)*

*6100300 (SLC6A6)*

*3120133 (E2F6)*

*4780280 (PLEKHH1)*

*610131 (SH3BP2)*

*70451 (CTPS)*

*4730451 (AA536743)*

*380133 (IL6)*

*770435 (ADAM7)*

*540010 (D930030O05RIK)*

*2940390 (TNFRSF5)*

*4780242 (1200009O22RIK)*

*2340358 (IFITM3)*

*6510524 (T2BP)*

*110541 (COPEB)*

*770288 (RAI14)*

*770026 (HPGD)*

*7000592 (CCL12)*

*3840446 (5730410E15RIK)*

*2630019 (NGFB)*

*5130685 (DGAT2)*

*6840056 (TNFRSF4)*

*3120397 (TEF)*

*730577 (H2-BF)*

*5550609 (IL13RA1)*

*5220400 (SLC43A3)*

*6660242 (UBE1L)*

*3710735 (HEMP1)*

*6660176 (RHOJ)*

*940731 (D11ERTD736E)*

*2970711 (RGS4)*

*4730088 (CD69)*

*3290315 (PER1)*

*2970433 (EFNB1)*

*1170039 (CHI3L1)*

*2260402 (OAS1B)*

*6370142 (ERDR1)*

*6420286 (D030015G18RIK)*

*6840446 (XDH)*

*4060070 (E030025P04RIK)*

*840168 (CYP4F15)*

*1770047 (EDN1)*

*1570594 (SPRY4)*

*1770170 (4933417E01RIK)*

*6290402 (CXCL13)*

*1170687 (UBE2L6)*

*4540519 (MLP)*

*4120504 (PDZK3)*

*4280372 (BRCA2)*

*2850338 (GPR84)*

*4760692 (SOCS2)*

*1770750 (ISG20)*

*1230017 (1110012L19RIK)*

*2690133 (1110032E23RIK)*

*5390441 (GJB6)*

*3290020 (TMEM2)*

*3710341 (STAT3)*

*1340494 (9130213B05RIK)*

*780731 (9130218O11RIK)*

*6590066 (AI132321)*

*3710072 (IL28RA)*

*3450128 (IL18BP)*

*3610095 (PBEF1)*

*2060014 (4930599N23RIK)*

*4810338 (MRVI1)*

*4560494 (THBS1)*

*2640592 (PLK3)*

*3060440 (PDGFB)*

*630504 (PSMB10)*

*2850093 (4930422J18RIK)*

*2680309 (IGFBP4)*

*6020487 (VAV1)*

*6760647 (EDN2)*

*4540048 (DSCR1)*

*2370292 (TBXA2R)*

*60148 (PLEK2)*

*3130497 (C730049P21)*

*4850600 (2310007B03RIK)*

*870504 (SMAD6)*

*1980066 (MSR2)*

*5290400 (P2RY6)*

*870025 (CAV1)*

*870347 (CYGB)*

*2570739 (MID1IP1)*

*1090673 (CD97)*

*6650093 (CASP4)*

*5130546 (PAWR)*

*1340450 (GPX3)*

*5890022 (SEMA4A)*

*870019 (PRKCB)*

*4560609 (2810474O19RIK)*

*2450435 (GPR88)*

*2650309 (MSX1)*

*1740253 (LRP4)*

*2900019 (PLAC8)*

*1660451 (VAPB)*

*670300 (SLC39A14)*

*3990707 (CX3CL1)*

*6510075 (IFITM1)*

*4150750 (CXCL12)*

*6020504 (RASGRP3)*

*6940577 (FMNL3)*

*6220309 (2410008K03RIK)*

*6590047 (8430417G17RIK)*

*770446 (C430010P07RIK)*

*6840292 (6330442E10RIK)*

*2370129 (GFPT2)*

*2760551 (TNFAIP8)*

*3520411 (GM644)*

*4230707 (LOC384410)*

*7050528 (S100A9)*

*2640400 (BC024955)*

*6020114 (ZC3HAV1)*

*2340332 (CSNK)*

*1850408 (CIRBP)*

*7050091 (KCTD12)*

*610161 (ICSBP1)*

*3170440 (ALOX5AP)*

*7040154 (GADD45A)*

*4200181 (SNRP70)*

*1780161 (ACE)*

*4570128 (USP53)*

*2570524 (CLDN3)*

*70138 (PFKP)*

*5290403 (PPP1R1B)*

*6510072 (DSIP1)*

*610685 (MAP3K3)*

*460592 (ZCCHC2)*

*1740068 (GLYCAM1)*

*1770520 (A430106J12RIK)*

*2450020 (PHLDA1)*

*3390338 (LGALS4)*

*840170 (JUN)*

*5050711 (4632417D23)*

*4230403 (OLFR1094)*

*3990551 (HIVEP3)*

*5700397 (OGFR)*

*6290040 (RASSF4)*

*4730280 (PPAP2B)*

*4730538 (4930555L03RIK)*

*2370279 (UNC5B)*

*4850164 (MT1)*

*2510471 (PLVAP)*

*4120142 (KCTD16)*

*5700131 (C1QG)*

*3850332 (E2F6)*

*450390 (MRGPRA3)*

*2260333 (SLC41A2)*

*6520735 (TNFRSF1A)*

*6840364 (MX1)*

*1340040 (NDRG1)*

*1740497 (MLP)*

*2640180 (P2RY2)*

*5390044 (IRF2)*

*4480128 (1190002H23RIK)*

*6450152 (PLK2)*

*6220010 (4932408M16)*

*5720008 (PLCG2)*

*3450019 (SLCO2B1)*

*4810280 (HES1)*

*6450670 (PFPL)*

*6350541 (TEK)*

*6040471 (ROBO4)*

*3140286 (ADAMTS1)*

*5550066 (CPM)*

*2230605 (DDIT4L)*

*2370156 (SLCO3A1)*

*4200184 (BC024537)*

*4540577 (CTGF)*

*3520022 (ZFP46)*

*1050239 (1500003O03RIK)*

*2630368 (RTN4RL1)*

*3850072 (TPM4)*

*6510039 (PSMB10)*

*2350113 (AS3MT)*

*1850020 (LGI1)*

*4070441 (PKIA)*

*870402 (CCNG1)*

*5420300 (HSPB1)*

*2190403 (D15ERTD735E)*

*6180253 (NR1D1)*

*5690440 (2310003H01RIK)*

*520181 (MFAP4)*

*770279 (ZBTB5)*

*4070341 (PSME2B)*

*510019 (4933405K18RIK)*

*5130372 (CCRN4L)*

*6660563 (D13BWG1146E)*

*7100368 (SELE)*

*5130301 (SLC9A3R2)*

*5890110 (PIK3CG)*

*6590286 (TGIF)*

*6770524 (MYT1)*

*4050086 (ITGA8)*

*6840129 (AOC3)*

*3520438 (FGD5)*

*6420154 (AOX3)*

*7040044 (H2-T9)*

*1690487 (4631426J05RIK)*

*630025 (PHF11)*

*5360253 (UGCG)*

*4210180 (5430435G22RIK)*

*2630497 (PLEC1)*

*4760735 (CYP1B1)*

*3290494 (ARID4B)*

*3140082 (MUC1)*

*2190048 (FKBP5)*

*6350008 (SFRS5)*

*1500070 (SLC1A3)*

*6220446 (RHBDL6)*

*7100463 (OLFML3)*

*6130397 (RALB)*

*5390465 (C2)*

*430288 (THBS1)*

*5130154 (ADRA2C)*

*580121 (ZFP90)*

*6400082 (N/A)*

*780750 (FCGR2B)*

*4810050 (RMCS1)*

*6860121 (DUSP1)*

*580332 (H2-K1)*

*5290487 (BZRAP1)*

*3170451 (OAS1G)*

*2340632 (4933405A16RIK)*

*6350487 (ICRFP703B1614Q5.5)*

*460441 (CARS)*

*2120066 (4833439L19RIK)*

*6200551 (GPR128)*

*6180500 (2900042B11RIK)*

*6040008 (EXO1)*

*1450056 (DCTN1)*

*1450066 (CD79B)*

*5290452 (AKAP8L)*

*2100717 (MYD88)*

*4010133 (OLFR68)*

*6350010 (S100A10)*

*2450133 (ARID5A)*

*6770025 (DOCK10)*

*2350070 (BC030183)*

*4150138 (TOB1)*

*2470193 (GSTA3)*

*540079 (CDC42EP2)*

*4230372 (PLEKHA4)*

*1770086 (LOC213233)*

*780082 (H2-L)*

*430592 (2010001H14RIK)*

*2360711 (E130006D01RIK)*

*460021 (AVPR2)*

*4760095 (D17H6S56E-5)*

*670563 (SMTN)*

*6220092 (SLC8A2)*

*670338 (CLDN11)*

*6040441 (CHN1)*

*6860010 (HPCA)*

*4610100 (ADCY2)*

*5290692 (PIGA)*

*5890487 (SCARA3)*

*2650433 (AI428936)*

*450292 (CUGBP1)*

*4070601 (SOX6)*

*2370338 (ZDHHC21)*

*6420397 (TBC1D4)*

*6220133 (BACE2)*

*5220450 (GOT2)*

*6130086 (ZFP275)*

*6040369 (KLK1)*

*5220398 (A930006J02RIK)*

*3800035 (SNX10)*

*1740577 (ZFP99)*

*5270121 (RBM5)*

*1230332 (STARD5)*

*4670044 (N/A)*

*4920164 (ZFP313)*

*3390471 (CD86)*

*6370131 (DSG1A)*

*770164 (MUC1)*

*2650142 (HIC1)*

*3800300 (HRASLS)*

*870563 (WSB1)*

*2340446 (PGM5)*

*130593 (SERTAD4)*

*6420095 (E130102H24RIK)*

*5420441 (IGFBP4)*

*450022 (MAPK12)*

*5290184 (SCARF2)*

*6590280 (GATA2)*

*3870131 (MPRA)*

*450075 (2010305A19RIK)*

*870324 (IDB2)*

*6400671 (PLEC1)*

*3450463 (FSCN1)*

*4070020 (FTCD)*

*6180400 (4933400F01RIK)*

*450592 (IL8RB)*

*4120068 (PALM2)*

*430725 (CHI3L4)*

*5290577 (TGFBR3)*

*4610390 (ARRDC3)*

*4670672 (BC003277)*

*5080162 (WNT5B)*

*6180528 (N/A)*

*4060524 (PIAS3)*

*6940053 (BCL9L)*

*6770372 (ZFP467)*

*7100309 (HCLS1)*

*380538 (RHBDF1)*

*2470102 (KCNE4)*

*4070279 (D11ERTD603E)*

*380056 (MITF)*

*6520204 (CARD10)*

*6370671 (ASPH)*

*110377 (RAI1)*

*5550706 (ABCA9)*

*770750 (ITGA10)*

*520484 (2310057J16RIK)*

*6760093 (ZFPM1)*

*6180372 (TUSC3)*

*4610541 (TPD52)*

*4230056 (COBLL1)*

*5860746 (4631403P03RIK)*

*7000446 (1110003A17RIK)*

*3120086 (ELTD1)*

*3190025 (RIPK1)*

*450184 (A030013N09RIK)*

*6400204 (CDC5L)*

*3940082 (LCN5)*

*460300 (GOLGA4)*

*2340601 (PYCS)*

*1190348 (HNRPK)*

*3060148 (OLFR507)*

*4920162 (TPM4)*

*3450154 (MUG2)*

*4070324 (E2F6)*

*60097 (HSPD1)*

*450021 (LOC330776)*

*460546 (AI413631)*

*5420541 (SAV1)*

*2260162 (EEF1A2)*

*5890435 (MAP3K14)*

*4010647 (DAXX)*

*1090139 (SAA2)*

*6620136 (1200002N14RIK)*

*430673 (SYNPO2)*

*940519 (POLA2)*

*2850707 (PSMB8)*

*460008 (IL12B)*

*4070110 (SLC29A1)*

*6290022 (FOXP4)*

*1740575 (COL4A1)*

*3450072 (1300019C06RIK)*

*4610592 (1810008K16RIK)*

*2940315 (CFI)*

*2510142 (GADD45G)*

*510372 (RASSF4)*

*5550347 (AKAP2)*

*130100 (CAR4)*

*1400152 (RNF4)*

*3440411 (BC030863)*

*2940358 (CRSP6)*

*4730685 (2310014H01RIK)*

*6110170 (LDB2)*

*770008 (5031400M07RIK)*

*3710333 (HIST1H2AH)*

*4280270 (ENG)*

*520519 (1200013B08RIK)*

*3060136 (V1RF3)*

*4210750 (MYL9)*

*460184 (PHOSPHO1)*

*450053 (PAPD1)*

*460019 (OLFR361)*

*2370685 (CAMK2D)*

*510040 (F830020C16RIK)*

*4210647 (4930519B02RIK)*

*1400671 (CDC2L2)*

*5270671 (MATN4)*

*5720025 (ROCK2)*

*6450181 (CLIC4)*

*5700324 (DPYSL2)*

*6220131 (MUC1)*

*2480427 (MEF2C)*

*2350441 (9030625A04RIK)*

*2680670 (6820428D13)*

*3800435 (AMMECR1)*

*2680402 (SIAT7B)*

*3140093 (H2-Q8)*

*2810168 (CXXC6)*

*6940195 (ZSWIM4)*

*3830673 (ERBB2)*

*4210403 (SERPINE1)*

*5050204 (PDXP)*

*4070093 (BC062109)*

*2640280 (PPP1R15B)*

*50594 (HSPA12B)*

*4230168 (RAD51L3)*

*2060079 (N/A)*

*2060397 (CNOT2)*

*6650519 (D11ERTD498E)*

*2340112 (REM1)*

*3390300 (1500003O03RIK)*

*3120142 (PNP)*

*2230735 (GPSM1)*

*130577 (B230342M21RIK)*

*6940242 (GMFG)*

*4280300 (OLFR1499)*

*2350170 (OLFR609)*

*6450450 (MLZE)*

*6980148 (ART4)*

*670685 (4833414E09RIK)*

*2810538 (IFRG15)*

*4280204 (DSCR5)*

*840364 (SYT1)*

*6200575 (NCK1)*

*6450377 (PTF1A)*

*450671 (PRICKLE1)*

*4050368 (SUV420H2)*

*2360435 (EDARADD)*

*6620129 (NAV1)*

*6370647 (SLC11A1)*

*2350408 (GADD45B)*

*5570040 (3110032G18RIK)*

*5890471 (AKAP3)*

*3060162 (BC013481)*

*1170671 (BC051244)*

*130605 (AK1)*

*6020601 (BING4)*

*6130619 (PRSS21)*

*4050551 (TFRC)*

*4280309 (EFNA4)*

*4230131 (LBA1)*

*6760487 (BST2)*

*4200136 (PARK2)*

*5360347 (MAP3K1)*

*6400300 (PDK4)*

*7050156 (2010315L10RIK)*

*2370041 (GCM2)*

*6200239 (GLYAT)*

*6350068 (HP)*

*4280068 (BTBD2)*

*3390593 (PER2)*

*460132 (BC042513)*

*6290519 (SIAH1B)*

*5670170 (1700094D03RIK)*

*580731 (NOL5)*

*130066 (SCX)*

*110524 (ENPP6)*

*6200541 (KCNK13)*

*3990095 (TMPRSS6)*

*1980100 (4933430F08RIK)*

*460048 (BC026744)*

*5890253 (9930017A07RIK)*

*6450433 (2310057J18RIK)*

*6760014 (XPO7)*

*6130707 (TRP53)*

*1740091 (OLFR414)*

*4850215 (9130401M01RIK)*

*4230435 (CCM1)*

*770670 (NPAS2)*

*6180411 (2610312E17RIK)*

*5720193 (IGH-6)*

*6760176 (4931432E15RIK)*

*2650605 (4732473B16RIK)*

*540348 (5330431K02RIK)*

*3830075 (RELA)*

*4150551 (9230112O05RIK)*

*5890047 (IL6ST)*

*4120079 (COL13A1)*

*610577 (IFNGR1)*

*3710528 (STX11)*

*1170433 (TCAM1)*

*4230576 (KCNK7)*

*6760047 (3110043J09RIK)*

*1780446 (H2-L)*

*1940133 (ORC5L)*

*1740121 (RORC)*

*1990463 (2410015B03RIK)*

*6200279 (DOCK10)*

*3610440 (ACAT2)*

*2570088 (1700019D03RIK)*

*2470609 (SPP1)*

*2360041 (RGS11)*

*6770717 (LYZS)*

*4280292 (LRRN2)*

*870446 (SNX16)*

*6180100 (NEK11)*

*4670133 (4930532L20RIK)*

*2650671 (OLFR745)*

*1500332 (INSIG1)*

*5270551 (SOX17)*

*1990300 (HOMER1)*

*5670091 (OPN4)*

*70215 (PDHB)*

*3940519 (AI646023)*

*6520010 (9130416B15)*

*6180670 (PYCR1)*

*2320333 (1500041J02RIK)*

*4230050 (ROPN1)*

*2360592 (IGFBP5)*

*6200280 (CD86)*

*1450019 (TIMM10)*

*6450131 (BC022150)*

*3800020 (AXOT)*

*5860048 (A430091O22RIK)*

*3170068 (DST)*

*3800400 (5730466H23RIK)*

*4610400 (ERBB4)*

*5220093 (QK)*

*5290242 (9930014A18RIK)*

*7000609 (GRIN1)*

*6980184 (AGPS)*

*1690070 (AI929863)*

*1780538 (SLC15A2)*

*6620039 (A2M)*

*6040170 (CYP2J6)*

*5340519 (DNM)*

*2360673 (1110017O22RIK)*

*6400288 (MEOX2)*

*3190403 (4930406H16RIK)*

*6380301 (D3WSU161E)*

*3130348 (A430005L14RIK)*

*6020132 (UTS2)*

*2450441 (FOXJ2)*

*2450711 (RGMA)*

*2260022 (ATP2B3)*

*3060520 (TNMD)*

*6220020 (PRG3)*

*2370347 (C430004E15RIK)*

*770438 (SLCO1A5)*

*2850601 (SLC13A3)*

*6760458 (2410004P03RIK)*

*3450068 (HLX1)*

*4060093 (TCFE2A)*

*1050136 (LRIG1)*

*4120047 (CDC6)*

*1990524 (NUPR1)*

*6520044 (CBFA2T3H)*

*1660463 (CUEDC1)*

*4200605 (A230053A07RIK)*

*2650161 (PAOX)*

*1340390 (MEOX1)*

*450368 (4933417L02RIK)*

*2370047 (SPI16)*

*6450497 (A130004G07RIK)*

*2970020 (GUCY1B3)*

*2190504 (CABLES1)*

*6450215 (TAL2)*

*4920520 (PNPLA2)*

*5670184 (5730592L21RIK)*

*7040239 (TAL1)*

*4120440 (MAFG)*

*6380497 (SBP)*

*4730072 (IL2RB)*

*3610075 (RPL21)*

*4210309 (CLDN13)*

*460750 (EPHA4)*

*1580528 (TRIM56)*

*4810152 (ZC3HDC1)*

*6290093 (ALOX12)*

*1660338 (CRYGC)*

*1190369 (KRT1-23)*

*3060075 (OLFR1351)*

*6550204 (H2-Q2)*

*2640008 (MAN2A2)*

*6980368 (ATRNL1)*

*50114 (ACO1)*

*4230348 (OLFR1395)*

*450048 (0710001E13RIK)*

*1940022 (ARHGAP4)*

*460446 (COL2A1)*

*7050088 (2410118P20RIK)*

*1940040 (SEPTIN 9)*

*4280537 (COL25A1)*

*2060605 (HRB)*

*5550278 (TNFSF8)*

*730484 (CTSK)*

*3710671 (RAB9)*

*5900113 (4933426M11RIK)*

*6760575 (RHOG)*

*1170347 (SOCS6)*

*70592 (ZNRF1)*

*4570068 (CXCL12)*

*2650403 (0610009F02RIK)*

*6020451 (1700025G04RIK)*

*460632 (OLFR541)*

*6590168 (TOR3A)*

*6370112 (AQP4)*

*540093 (SLC38A5)*

*6900086 (UGT1A6)*

*4670114 (4930511N19RIK)*

*630537 (SMAD1)*

*6380168 (HMMR)*

*510044 (ANP32A)*

*450731 (AMN)*

*6400093 (NR3C2)*

*6450632 (FBXW11)*

*3990075 (CAMTA2)*

*70487 (SERPINB2)*

*3060041 (TRAF4)*

*1660128 (CACNA1C)*

*2690364 (A630018G05RIK)*

*6450128 (ALS2CR2)*

*6550162 (A730098D12RIK)*

*6370064 (LOC13909)*

*6180020 (6030422M02RIK)*

*1500280 (PHGDH)*

*5860039 (TMEM24)*

*450427 (A530089L17)*

*670592 (1200015N20RIK)*

*4050541 (COL9A3)*

*1170021 (TCFCP2L2)*

*2970500 (PLCD3)*

*1190368 (KIF22)*

*1740278 (1110018J23RIK)*

*6380551 (ACCN4)*

*5390725 (CPXM1)*

*460242 (OLFR497)*

*3850575 (SELENBP1)*

*4670438 (D130012P04RIK)*

*7040021 (IL13)*

*3130242 (YEATS4)*

*4280056 (9930111J21RIK)*

*2230020 (TYROBP)*

*1980161 (AI194308)*

*50088 (H2-T22)*

*6400450 (A630031M04RIK)*

*6420600 (NAALAD2)*

*110273 (ISLR)*

*4280095 (SPT1)*

*2510110 (2810474O19RIK)*

*1580239 (NPR3)*

*5390411 (2310046A06RIK)*

*6180300 (DHX29)*

*2030427 (BC061259)*

*3840184 (FCGR3)*

*2190193 (BMPR1A)*

*6100673 (4930471O16RIK)*

*2060110 (SH3BGRL)*

*1500100 (2500003M10RIK)*

*2030113 (CASKIN2)*

*460064 (0610040J01RIK)*

*2340110 (CSF1R)*

*4210524 (HIST1H4B)*

*5220017 (AIF1)*

*6650450 (1810033A06RIK)*

*460600 (FBXO8)*

*4230020 (LAF4L)*

*2350132 (GDF1)*

*6180739 (AV216087)*

*510075 (KY)*

*6200170 (BK)*

*2640411 (C330012H03RIK)*

*6770537 (BC004728)*

*5050403 (1500010G04RIK)*

*4610373 (1300013J15RIK)*

*3870112 (DST)*

*6290170 (RRAS2)*

*450446 (C330017I15RIK)*

*380047 (NTSR2)*

*4060082 (ITGB2L)*

*3390692 (SREBP2)*

*7050110 (D11MOH35)*

*6350519 (BC013481)*

*2370184 (DUSP2)*

*1740286 (RBP7)*

*5340452 (VLDLR)*

*6100070 (RNF24)*

*6370494 (COBLL1)*

*540181 (MCM6)*

*6980736 (SH2BP1)*

*4050692 (TAF13)*

*940131 (SLFN1)*

*4560037 (DEK)*

*6450195 (PTGS1)*

*6180095 (BOLL)*

*1230605 (SLC25A25)*

*4230129 (LOC239447)*

*3870215 (PELI1)*

*7000014 (ABCA8A)*

*4610450 (NCB5OR)*

*510010 (SNX3)*

*3990403 (FPR-RS6)*

*2320097 (EGFL5)*

*2470014 (IAN6)*

*4230066 (4930590J08RIK)*

*4230358 (SCA10)*

*3450086 (OLFR1126)*

*3440494 (HUS1)*

*1980091 (NDUFAB1)*

*6380333 (WDR17)*

*430707 (2310037I24RIK)*

*3990082 (SPIN)*

*4670131 (2610019A05RIK)*

*2650372 (SELENBP2)*

*2450070 (CDKL5)*

*60088 (KCNIP2)*

*380528 (CCND3)*

*1940348 (GPRK5)*

*510333 (2900010J23RIK)*

*450072 (4930442L21RIK)*

*70273 (5830426I05RIK)*

*6220025 (SMYD5)*

*5290524 (POLN)*

*5050239 (HIST1H2AN)*

*2350048 (BC019206)*

*430593 (NEU4)*

*4070347 (ADAM12)*

*1980537 (TGFBR2)*

*1940102 (MYH4)*

*5340195 (FGD2)*

*730736 (PAQR4)*

*4150242 (CD83)*

*4610411 (TBX15)*

*4070168 (D7BWG0611E)*

*2320398 (OLFR447)*

*5720167 (G6PDX)*

*6200707 (SLC25A27)*

*4230524 (A630005I04RIK)*

*6420358 (CHST2)*

*2970088 (L259)*

*460458 (SEMA6C)*

*6180152 (DPH2L1)*

*1660193 (PIK3R2)*

*4610053 (OLFR1451)*

*5420079 (LUM)*

*3060092 (LOC238447)*

*6420601 (CYP2C29)*

*4280162 (BTBD14B)*

*3060739 (GPIHBP1)*

*510092 (4930525K21RIK)*

*4730020 (BIRC2)*

*1450021 (COLEC11)*

*1090309 (TIE1)*

*460082 (V1RE12)*

*2470148 (2700055K07RIK)*

*6900133 (EDNRA)*

*6450739 (GALR2)*

*4610594 (SMARCA3)*

*4670278 (9430034D17RIK)*

*6200139 (CYP2B20)*

*4060600 (FREQ)*

*730021 (GABPB1)*

*6110309 (MARVELD1)*

*5570551 (LYPLA1)*

*4540465 (GCNT2)*

*1500750 (ADPRTL3)*

*5290338 (BC037034)*

*6130253 (PPP1R3B)*

*3940731 (THEA)*

*1170452 (TMEM32)*

*3870619 (LOC384422)*

*6980301 (GAMT)*

*4670619 (ITGB7)*

*1740113 (EVL)*

*6380452 (GJA4)*

*4060075 (D330045A20RIK)*

*4210706 (ANK1)*

*380497 (HTR2C)*

*4280066 (PHF17)*

*2810551 (PDLIM2)*

*5340750 (1200009I06RIK)*

*6100315 (ULK1)*

*3450181 (1110059P08RIK)*

*450711 (IL1RAP)*

*4730735 (6330406I15RIK)*

*1690292 (ENAH)*

*630519 (LY96)*

*6200079 (C130058G22RIK)*

*4200300 (G630039H03RIK)*

*6100440 (H2-T17)*

*4230128 (HCPH)*

*6220750 (HSPG2)*

*460025 (GZMC)*

*2480053 (POLR3A)*

*5860750 (DR1)*

*2510671 (JUP)*

*2370021 (RNF13)*

*4610008 (HNRPR)*

*50465 (SH3D4)*

*6370242 (SUV39H2)*

*1980458 (2310058J06RIK)*

*6110605 (LY6A)*

*2350438 (2310016F22RIK)*

*7040010 (PSMC2)*

*2970142 (TRIM34)*

*3710438 (4931406C07RIK)*

*4210047 (2410004H05RIK)*

*1740368 (4631423B10RIK)*

*4050026 (3110007P09RIK)*

*1410390 (2410004N11RIK)*

*4280279 (C430014N20RIK)*

*6450040 (PLCD4)*

*1500021 (CHL1)*

*60504 (NID1)*

*2230377 (MFAP2)*

*4070438 (OLFR895)*

*4670458 (A930017E24RIK)*

*4670035 (EEF2)*

*2320139 (LACTB)*

*4730112 (6820408C15RIK)*

*1740280 (RAB12)*

*1400594 (1700018O18RIK)*

*3190397 (PPAP2B)*

*3520167 (BC037135)*

*2320673 (FZD10)*

*6550358 (GCH1)*

*6450446 (D930038M13RIK)*

*6370446 (MBL2)*

*5340603 (BC036333)*

*6180458 (5930406N14RIK)*

*460088 (2610318I18RIK)*

*4070673 (SSB4)*

*1410131 (2410141K03RIK)*

*6100692 (JAK2)*

*2370070 (SFMBT1)*

*5570017 (2810409K11RIK)*

*4230162 (RANBP2)*

*2450161 (PSCA)*

*2350411 (BTG2)*

*4210113 (BARHL1)*

*6770446 (SEPHS2)*

*2970066 (4933402E13RIK)*

*3390458 (C330016O10RIK)*

*3870041 (AW494914)*

*7000500 (RNF150)*

*6450044 (CDKN1B)*

*6020692 (MGEA6)*

*670446 (PSAT1)*

*7040603 (BHLHB2)*

*7040465 (ATBF1)*

*380020 (ZIC3)*

*1770010 (GATA6)*

*1690692 (D11ERTD530E)*

*6450082 (A730037L19RIK)*

*460092 (1110033J19RIK)*

*2760333 (TM9SF1)*

*5050086 (MYLK)*

*4210044 (IFI205)*

*520341 (2700084L06RIK)*

*4050706 (CAPN5)*

*6200193 (PLAGL1)*

*2360021 (TMOD4)*

*3190113 (IFIT3)*

*4730672 (IFNAR1)*

*2470093 (UBE2V1)*

*3850725 (9230106L14RIK)*

*6770273 (AV006891)*

*1230008 (2610024E20RIK)*

*3870301 (H2-EA)*

*5570446 (GULP1)*

*5720408 (PDYN)*

*540020 (CRAT)*

*840504 (V1RA4)*

*3170288 (CYP4A10)*

*5420647 (THSD1)*

*4070022 (GPR34)*

*5690372 (D11ERTD636E)*

*2970072 (RNF111)*

*1240333 (SUPT16H)*

*3710487 (CACNB1)*

*6380746 (N/A)*

*6510139 (1500004A08RIK)*

*4280364 (A030005K14RIK)*

*5340050 (SLC37A1)*

*1230066 (UCHL1)*

*50204 (PIAS3)*

*2510546 (S100A16)*

*3940593 (TRIM10)*

*5860195 (3632451O06RIK)*

*6450671 (SMAD3)*

*3360452 (EPM2AIP1)*

*3850309 (E030010A14)*

*6450279 (PDGFRB)*

*3450070 (OAZIN)*

*2810039 (D17ERTD288E)*

*1050273 (RGS9)*

*2570110 (2310046K10RIK)*

*5910075 (COL4A3)*

*2510706 (MAT2B)*

*50731 (SLC37A3)*

*3440142 (BC004022)*

*3120035 (MS4A6D)*

*2970601 (OLFR763)*

*4280041 (SVS5)*

*1240056 (9230113P08RIK)*

*2680168 (N/A)*

*4070112 (RAB7)*

*6200110 (SEMA5A)*

*4850609 (H13)*

*3830176 (GEMIN6)*

*4200524 (ACCN3)*

*4210717 (UBE2Q)*

*3800707 (1110054N06RIK)*

*940504 (RHOE)*

*2260008 (HCFC1)*

*2030133 (CACNB4)*

*6040524 (OLFR854)*

*1240131 (ATBF1)*

*4210022 (HIST2H2BB)*

*4060095 (MRGPRB2)*

*2060446 (TGFBI)*

*2480500 (HOOK1)*

*3140520 (2600003E23RIK)*

*4010167 (OLFR808)*

*4070017 (SCN5A)*

*2480450 (DUSP16)*

*430487 (V2R2)*

*3830168 (GSN)*

*6860707 (4930470D19RIK)*

*3520168 (1190005I06RIK)*

*1990129 (TMC7)*

*2690471 (ITIH4)*

*450070 (ZFP67)*

*6510154 (1500016L11RIK)*

*1690427 (5033430I15RIK)*

*4730170 (2310061J03RIK)*

*60300 (FEM1C)*

*4570735 (HIST1H4K)*

*6450341 (PNLIP)*

*2900707 (NET1)*

*2320048 (CD96)*

*460647 (C730036D15RIK)*

*4810048 (2310057M21RIK)*

*580619 (TRIM33)*

*4210079 (CCKAR)*

*3140537 (C630023L15RIK)*

*540458 (SYT4)*

*5890576 (LASS6)*

*2760136 (ABCA9)*

*4670398 (HNRPA1)*

*1500170 (A930008G19RIK)*

*6400364 (PTP4A3)*

*50075 (POMT2)*

*2760497 (FLRT3)*

*2030195 (DDX56)*

*4610010 (B230399N07)*

*7000064 (2810048G17RIK)*

*4230563 (SEMA3E)*

*6450465 (ACCN2)*

*4060563 (KRT1-18)*

*5220707 (6030440P17RIK)*

*6350292 (AGA)*

*1450520 (TJP3)*

*2120121 (HOXA2)*

*4200056 (E2F7)*

*1980731 (PLIB)*

*1660010 (DNAJA1)*

*4120092 (1700019M22RIK)*

*4610068 (2010004O20RIK)*

*4210184 (LOC56628)*

*5890390 (CAR3)*

*6400193 (RNF138)*

*510451 (BC003236)*

*5390600 (ADD3)*

*2340497 (RNF2)*

*5900121 (TRIM29)*

*2350575 (IER5)*

*3800193 (OGN)*

*6180044 (SDK2)*

*450687 (N/A)*

*3060324 (UNC5A)*

*430097 (DIA1)*

*6370035 (PDE4A)*

*1570215 (N/A)*

*2510451 (SLC22A5)*

*50025 (OLR1)*

*3800112 (SH3GL3)*

*6940706 (SPRED1)*

*4480373 (LYPLA1)*

*4610619 (SPINK4)*

*450497 (HNRPM)*

*6660722 (SLC23A2)*

*6650603 (TNF)*

*5570368 (CD6)*

*6980450 (EPGN)*

*6400338 (ADPRT1)*

*6200494 (HNRPA2B1)*

*1170446 (A630052C17RIK)*

*3130377 (IGF1)*

*6220035 (2610027C15RIK)*

*6290717 (OPRS1)*

*6900315 (UBTD1)*

*1230180 (D15ERTD785E)*

*3140408 (CFC1)*

*3360333 (GPC5)*

*5670152 (BC038156)*

*1410524 (TLR1)*

*6180139 (AI593442)*

*5570390 (WBSCR16)*

*4060438 (FMNL2)*

*5700091 (N/A)*

*3610039 (ZFP94)*

*4050148 (SLC26A9)*

*6200576 (6230405M12RIK)*

*5360463 (RAD23A)*

*1780577 (KCNE2)*

*4230113 (CYP1A1)*

*2100164 (2010301N04RIK)*

*4050035 (ADAM28)*

*4230739 (IFI35)*

*1780672 (CSMD1)*

*1660692 (ADRBK2)*

*4200008 (VGLL1)*

*1740390 (COL27A1)*

*60162 (A630026H08RIK)*

*2120047 (KITL)*

*6200438 (1110027O12RIK)*

*3870026 (KDT1)*

*1230524 (N/A)*

*6980133 (HOXA11)*

*5290739 (PTHLH)*

*2810114 (SFRP5)*

*3610537 (SEC14L1)*

*540176 (GNGT2)*

*450161 (CRHR1)*

*2100128 (BMP6)*

*6550576 (EFNA4)*

*2940722 (AQP7)*

*4060390 (ERCC2)*

*5340142 (SLC38A3)*

*2900215 (DNAJB5)*

*7100528 (C530043K16RIK)*

*3290008 (JAK3)*

*6400400 (NG23)*

*4210082 (GGN)*

*6130338 (CHD4)*

*4210129 (TMEM25)*

*1170725 (LOC230872)*

*2680021 (AV249152)*

*6940167 (3222402P14RIK)*

*4070315 (ABCD2)*

*6370706 (MGC6357)*

*3390427 (CAMK1)*

*2230152 (NANOG)*

*110672 (6330415M09RIK)*

*1940402 (PRLPF)*

*4540603 (EXT1)*

*7100039 (ITPKB)*

*510095 (SIAT8E)*

*4760128 (SLMAP)*

*6900731 (RAB5B)*

*1980148 (9930021D14RIK)*

*6660068 (L3MBTL3)*

*4540398 (LSM5)*

*3440148 (DDX5)*

*730286 (RBM15)*

*6450687 (TRIM9)*

*520148 (PYGL)*

*6040358 (RAB32)*

*1780035 (ROCK2)*

*4570102 (1110019L22RIK)*

*2450176 (NR1H4)*

*1780390 (NKG7)*

*3140167 (GAD1)*

*1170286 (TRIM2)*

*3390746 (B3GALT3)*

*5570739 (CAST1)*

*1990324 (IL27)*

*3840632 (4931414L13RIK)*

*4850537 (4921511C20RIK)*

*4570463 (SAT1)*

*4610167 (C130048D07RIK)*

*6450487 (RBM11)*

*6940373 (N/A)*

*2360121 (STK35)*

*5700167 (RBM3)*

*6450609 (RTN4RL2)*

*3060010 (1700036D21RIK)*

*6380368 (NEDD4L)*

*5550593 (CACNA2D1)*

*1780324 (KCNIP2)*

*6380100 (TNFRSF21)*

*6350092 (1110030J09RIK)*

*4670020 (1110028E10RIK)*

*460075 (DEPDC1)*

*6220079 (N/A)*

*380504 (DIO2)*

*5860685 (CPD)*

*6520095 (LOXL4)*

*5290097 (NEUROD2)*

*3520035 (USP25)*

*730347 (OPN1MW)*

*6660136 (STK19)*

*2190280 (SPATA1)*

*3170270 (OLFR1277)*

*5720546 (RASSF2)*

*4920114 (2310047I15RIK)*

*2350239 (MARE)*

*2230110 (WDR31)*

*4060463 (ADH6A)*

*2260279 (GALNT2)*

*6100110 (2810408E11RIK)*

*460390 (HGD)*

*580040 (TLE3)*

*4560332 (IL15)*

*2260593 (MGAT3)*

*2230332 (MTAP1B)*

*510066 (LMX1B)*

*6660056 (NAB1)*

*6770047 (SCARB1)*

*4070594 (TCF20)*

*5390035 (SNTB2)*

*1570717 (2400010D15RIK)*

*1690348 (ZFP1)*

*840315 (CISH)*

*50148 (ANKS1)*

*2340433 (MYH2)*

*6900040 (PELI1)*

*6860088 (RABGAP1L)*

*2680403 (CALU)*

*6110402 (9630036L12RIK)*

*2510735 (CPLX1)*

*2370722 (2700050L05RIK)*

*2470068 (SNX2)*

*60332 (N/A)*

*4540239 (DHRS8)*

*3520347 (D6WSU163E)*

*5390750 (KIF13A)*

*1170576 (PSMD10)*

*6840494 (RPL27A)*

*4610403 (OBOX1)*

*2340338 (CYP24A1)*

*3390114 (NOTCH1)*

*4210333 (5430432M24RIK)*

*730020 (COL1A1)*

*2900672 (GDAP10)*

*3060066 (SYTL4)*

*2230390 (GNG2)*

*70195 (6820416H06RIK)*

*1340685 (PRKWNK1)*

*6400086 (MNAB)*

*840601 (B130017I01RIK)*

*6220138 (ESR2)*

*2650440 (LTBP3)*

*730463 (9130210N20RIK)*

*5080100 (EIF1A)*

*3170603 (PRKWNK1)*

*6040577 (6530401N04RIK)*

*580017 (2610001E17RIK)*

*540286 (DICER1)*

*3290154 (LRRN1)*

*7000364 (MYLK)*

*2760338 (SUMO1)*

*4920368 (GZMD)*

*460292 (PCDHGA9)*

*510014 (D430026P16RIK)*

*5420377 (RPS24)*

*450044 (4921511F01RIK)*

*580333 (MRPL1)*

*380368 (SLC6A14)*

*5900338 (RB1)*

*1090717 (REV3L)*

*1230082 (ACTA2)*

*3290390 (N/A)*

*6020010 (BTBD14A)*

*730044 (PSMD12)*

*3440041 (ENPP4)*

*4590427 (CYP51)*

*4120273 (IL2RG)*

*3060497 (LOC223672)*

*2680458 (STAT5A)*

*5670280 (5830417C01RIK)*

*4060411 (EML2)*

*6180131 (OLFR1145)*

*1190520 (BCAP31)*

*2690551 (CYSLTR1)*

*3830494 (SH3GL2)*

*430619 (OLFR1135)*

*3120372 (TRPV4)*

*1170603 (DDX28)*

*3360601 (C330046L10RIK)*

*5910402 (6430527G18RIK)*

*4070609 (BC034090)*

*6620315 (1810035L17RIK)*

*4230079 (HAP1)*

*130364 (1810073P09RIK)*

*2470402 (OLFR1093)*

*460041 (A630050E13RIK)*

*4730411 (PTK2B)*

*6100546 (MYO1F)*

*2810142 (AIM1)*

*510026 (CYP4X1)*

*3120091 (MYH9)*

*6040593 (PIK3C2A)*

*4850047 (1200003M09RIK)*

*70446 (KTN1)*

*6020465 (IL12RB2)*

*4050039 (9030624L02RIK)*

*4780059 (2400003L07RIK)*

*4200035 (FLRT2)*

*3520364 (SDCCAG1)*

*6650487 (5730446C15RIK)*

*7100092 (ALS2CR2)*

*1660044 (GPM6A)*

*6350750 (CABP1)*

*6400053 (DOLPP1)*

*2810022 (GM770)*

*6550487 (EPB4.1)*

*6900373 (STRA13)*

*1780563 (TXNRD3)*

*2760458 (1110037F02RIK)*

*2060195 (AKAP9)*

*2120603 (B230382K22RIK)*

*6180364 (POLR3D)*

*1990377 (SLC2A3)*

*1690341 (HADHSC)*

*4060309 (GORASP2)*

*460128 (1110001P11RIK)*

*1990484 (LOC384419)*

*6940292 (E330018D03RIK)*

*4060129 (MRO)*

*6110594 (CCNG1)*

*5700162 (CHES1)*

*520451 (GGA2)*

*4280348 (MFNG)*

*3990746 (OSTN)*

*2940040 (DOCK2)*

*4280170 (CEL)*

*3840215 (APOBEC3)*

*2650398 (UCK1)*

*770632 (4833441J24RIK)*

*580095 (ALDH3A1)*

*6420056 (MERTK)*

*1410204 (BRD4)*

*3870239 (CSPG3)*

*940402 (9430063L05RIK)*

*1990048 (F830045P16RIK)*

*5690735 (AW491445)*

*610520 (SPOCK3)*

*7000204 (STK32B)*

*3120044 (PCSK9)*

*2360706 (PHYHIPL)*

*1500711 (SPEER4F)*

*770112 (A230072I16RIK)*

*6370102 (A430056A10RIK)*

*2060458 (TANK)*

*2650471 (NUP50)*

*2230148 (DHCR7)*

*2370131 (CHST3)*

*3450114 (KPNA2)*

*450176 (ZFP385)*

*1990348 (SPARCL1)*

*6980066 (4833403I15RIK)*

*1500035 (1600022A19RIK)*

*2450736 (3830421F13RIK)*

*1690685 (XRCC4)*

*2350341 (LOC383540)*

*4200273 (A430005L14RIK)*

*6350731 (OLFR1055)*

*4760575 (NSG2)*

*3870086 (CYP2C39)*

*3800592 (6530401D17RIK)*

*4230138 (AMID)*

*2450377 (MGAT1)*

*6020446 (RBM6)*

*2690603 (TRIM35)*

*4560711 (SYTL3)*

*1340577 (2410019G02RIK)*

*1990040 (E430002D04RIK)*

*3290072 (BC023151)*

*1240537 (TNRC6)*

*4610433 (4933402D24RIK)*

*2100059 (TPM4)*

*1940411 (CD8B)*

*1940072 (2310057G13RIK)*

*1850458 (TAF9)*

*1780273 (PTGS1)*

*3780538 (RXRIP110)*

*360044 (A430005L14RIK)*

*1850411 (1700011K15RIK)*

*70161 (1110018J18RIK)*

*4210603 (OLFR1084)*

*3130072 (D230037D09RIK)*

*3450156 (DOK5)*

*1190433 (NPHP4)*

*1740072 (TPBPB)*

*6400139 (PTPRS)*

*2230050 (COL5A1)*

*4760369 (4632417K18RIK)*

*4610292 (1700010C24RIK)*

*5900066 (APOC1)*

*4280044 (CD209E)*

*430446 (MBD3L2)*

*6180280 (4931413A09RIK)*

*2190402 (EMILIN1)*

*2470092 (CSDA)*

*6200450 (BC029169)*

*450341 (DMC1H)*

*1660673 (RASSF4)*

*6110603 (1600012H06RIK)*

*3990465 (FV4)*

*4280176 (1200003I07RIK)*

*1230673 (BC030046)*

*3450053 (MDM2)*

*3450427 (RAPGEF5)*

*1500301 (TRIM31)*

*5340048 (0910001A06RIK)*

*4060538 (OLFR822)*

*2350148 (PCSK1)*

*4210576 (SLC17A6)*

*4610746 (OLFR742)*

*2760301 (RPA2)*

*2650156 (2810004A10RIK)*

*2970347 (PCDHA5)*

*2360397 (BCL10)*

*4810139 (PECAM1)*

*2350204 (1110039B18RIK)*

*460722 (FGFR1OP2)*

*2360133 (KLRG1)*

*5890706 (4921538N17RIK)*

*2340452 (4932417I16RIK)*

*4070692 (6330505F04RIK)*

*3870292 (RHOQ)*

*7050273 (DDR2)*

*2340609 (GCN5L2)*

*1740047 (MMRN2)*

*2630484 (2610510D13RIK)*

*2360497 (C2C)*

*6510112 (ZFML)*

*6550735 (CLECSF9)*

*2030390 (2310043N13RIK)*

*1090592 (1700010L19RIK)*

*5420195 (RPA2)*

*6400136 (HMGA2)*

*1980025 (OLFR140)*

*4610022 (APOBEC1)*

*6130452 (DPH2L1)*

*3520440 (BC027342)*

*6220050 (HOXC8)*

*5550377 (2210412D01RIK)*

*4920075 (9530033F24RIK)*

*2030097 (TRP53INP1)*

*2570563 (PDE1A)*

*380524 (HS1BP3)*

*6130402 (OLFR814)*

*7050047 (5830467E07RIK)*

*6760427 (5430413K10RIK)*

*1850056 (TAF6L)*

*3990204 (PLFR)*

*3450609 (KTN1)*

*780373 (DDIT3)*

*3450093 (D130011D22RIK)*

*1580044 (PITPNM2)*

*610551 (OTUB1)*

*1850593 (1700030B21RIK)*

*6450398 (4930562F07RIK)*

*6130079 (E130113K22RIK)*

*450079 (NRP)*

*6760671 (PRIMA1)*

*630176 (CAR5B)*

*1690050 (SCG3)*

*730398 (ZFP294)*

*630014 (CYP4F16)*

*2190215 (PDE11A)*

*6900044 (YSK4)*

*3830037 (4933432B09RIK)*

*1190064 (ZFP61)*

*3390446 (SRI)*

*5290446 (3230401I01RIK)*

*2030315 (ZFP617)*

*2900450 (VCAM1)*

*3450064 (OLFR771)*

*2510593 (NAP1L1)*

*1170647 (EGFL8)*

*2350358 (FGL1)*

*2230121 (BC027309)*

*5670050 (ABR)*

*4570070 (6530401L14RIK)*

*1580372 (GPRC5C)*

*130292 (ZFP106)*

*1090035 (DST)*

*6760131 (BACH2)*

*5420408 (2210409D07RIK)*

*4200563 (6430590A10RIK)*

*2510176 (BC037651)*

*3940053 (BCOR)*

*1770148 (PLAGL2)*

*6040056 (1700024B07RIK)*

*3060551 (TJP3)*

*6200347 (OLFR1340)*

*2320372 (1600022A19RIK)*

*4050372 (C330039G02RIK)*

*2630603 (DSG2)*

*6020333 (TOPBP1)*

*2260133 (HDAC9)*

*3940746 (PSCD4)*

*4230167 (CCL1)*

*1170731 (NKD2)*

*4010131 (RGS3)*

*450139 (4933401K09RIK)*

*4590731 (1700020H15)*

*3870593 (N/A)*

*770193 (OLFR860)*

*2100487 (SLC24A4)*

*5360717 (RCOR1)*

*1050397 (SFRS16)*

*3830465 (HADHSC)*

*610338 (3632431M01RIK)*

*1500092 (THBD)*

*1090286 (PRSS2)*

*2360484 (CRYZ)*

*3990010 (H2-M9)*

*4730332 (AI661438)*

*2510358 (SLC22A5)*

*6760136 (RARG)*

*4010102 (JARID1C)*

*2350037 (B830045N13RIK)*

*1500082 (ZIC4)*

*6900332 (RARSL)*

*6380647 (RAB27B)*

*1570168 (4921522K05RIK)*

*1580086 (ATP8A2)*

*2940114 (1110025G12RIK)*

*2190600 (TPK1)*

*1850161 (SPACA3)*

*3290471 (HNRPH1)*

*5290079 (SEPTIN 3)*

*5390400 (2410002F23RIK)*

*2190575 (AI840980)*

*6450546 (ENTPD6)*

*6940390 (AW046014)*

*4070041 (A930018P22RIK)*

*3170364 (GIP)*

*6200156 (CAST)*

*1980315 (SEMA4C)*

*1780398 (DOCK7)*

*3800288 (4930455C21RIK)*

*4230142 (WDFY2)*

*3830142 (LOC328526)*

*7100576 (G3BP)*

*3190114 (KLRD1)*

*3840050 (COTL1)*

*3870402 (RAB11FIP1)*

*6940400 (3830408P04RIK)*

*2680066 (LCP2)*

*2060332 (USP3)*

*2970358 (MYO1G)*

*4540717 (CATNBIP1)*

*4760408 (8430416H19RIK)*

*3450400 (PITRM1)*

*3130280 (6030446I19RIK)*

*4230440 (9830123M21RIK)*

*580152 (ASH2L)*

*4570097 (5830433M19RIK)*

*6400008 (A930038C07RIK)*

*6130575 (KIF2A)*

*1090131 (4930588N13RIK)*

*1580136 (OSTB)*

*4120450 (HIP1)*

*7100242 (TAGLN3)*

*4230092 (V1RC30)*

*3610167 (BC016198)*

*2190164 (ACTR5)*

*1580575 (H2-Q5)*

*7040088 (KPNA3)*

*1580056 (S3-12)*

*510152 (ASAH1)*

*6370364 (IL5)*

*2260403 (1190002J23RIK)*

*1050707 (ARID4A)*

*5080403 (KCNA2)*

*1500114 (A530065E19RIK)*

*2350039 (GULP1)*

*2030053 (PARK2)*

*1450600 (0610010F05RIK)*

*4670592 (LIP1)*

*6180685 (ZFP566)*

*380593 (SFRS2)*

*1940592 (ACAT3)*

*5420427 (CKLFSF7)*

*1940451 (BC018462)*

*1570546 (SHOC2)*

*3520487 (KPTN)*

*610528 (1810007M14RIK)*

*780092 (2700091N06RIK)*

*6040278 (1700011I11RIK)*

*4210181 (6430526O11RIK)*

*6940725 (CRABP2)*

*5700040 (OLFR599)*

*4230408 (CRSP7)*

*1410181 (FTSJ)*

*3190095 (CCNG2)*

*2970504 (MC2R)*

*1990441 (EDG8)*

*2940504 (TSSC1)*

*3940110 (CTPS2)*

*60348 (2900090M10RIK)*

*1940121 (AMOT)*

*6020500 (ASB9)*

*2810575 (ZRF2)*

*3440369 (REN1)*

*5900746 (TDPOZ1)*

*6380167 (AR)*

*2510687 (HSPG2)*

*5900528 (N/A)*

*1940162 (TGFB1)*

*2360500 (JUNDM2)*

*4230136 (5730509K17RIK)*

*2450204 (2610208E05RIK)*

*580377 (2810410A08RIK)*

*5860731 (SAT2)*

*2570026 (TCFCP2)*

*6200619 (4933425M15RIK)*

*2370458 (RABIF)*

*5420008 (NELL2)*

*3870139 (PLAG1)*

*2190278 (ROPN1L)*

*520377 (WDR8)*

*2230301 (ARHGAP9)*

*2060575 (1810054O13RIK)*

*2690097 (FNDC3)*

*6370292 (FASTK)*

*3140487 (APBA1)*

*1850441 (FRG1)*

*840731 (GNPDA1)*

*6290066 (ELMO1)*

*6020044 (1700001C14RIK)*

*3780092 (ZBTB7)*

*450398 (PCDH7)*

*2060347 (2400003C14RIK)*

*430739 (NAB1)*

*6590309 (SLC27A3)*

*4610364 (GLA)*

*6450706 (BMS1L)*

*730402 (4933428A15RIK)*

*1850451 (SLC30A3)*

*3850594 (CD109)*

*1850133 (KCNA7)*

*6370707 (NOTCH4)*

*6200300 (HR)*

*1990014 (PLSCR3)*

*1940167 (0610016J10RIK)*

*7050592 (KLHL8)*

*2370019 (SESN2)*

*6770180 (C1QR1)*

*1690014 (ELF2)*

*2850672 (D7ERTD743E)*

*3440403 (MYOCD)*

*2340519 (RUTBC1)*

*3450088 (OLFR594)*

*4670605 (AP3B2)*

*3450538 (OLFR113)*

*4060408 (YTHDC2)*

*2350446 (B3GNT1)*

*5910347 (SRD5A1)*

*2480059 (FBLN1)*

*1940148 (OXCT2A)*

*430113 (RBBP7)*

*4560465 (LHCGR)*

*1450435 (SCA7)*

*5420278 (NEUROG2)*

*6450438 (DCC)*

*5900609 (GJB2)*

*1090056 (F730038I15RIK)*

*6200010 (TNRC6)*

*730139 (SOCS1)*

*1990072 (4833420N02RIK)*

*6550402 (NSPC1)*

*360471 (CBARA1)*

*7040647 (SYT12)*

*6660300 (CAPN1)*

*3780400 (8430415E04RIK)*

*6200088 (MLLT3)*

*4230746 (VMD2L1)*

*6180452 (SLA)*

*2190458 (OLFR1046)*

*2030161 (9430076C15RIK)*

*5670369 (MYT1L)*

*1230132 (FBXO24)*

*5860433 (B230363K08RIK)*

*6110722 (RBMS3)*

*2690433 (CPT1B)*

*6660162 (PEX6)*

*6760292 (MYO1D)*

*2640632 (V1RC24)*

*4590333 (CNOT6L)*

*4780100 (4933413G11RIK)*

*2350215 (9130214H05RIK)*

*2340494 (BCAS2)*

*6350605 (AV312086)*

*1450088 (A330043L12)*

*1940270 (MYOC)*

*6980433 (NF1)*

*6200270 (B630009I04RIK)*

*450095 (ATP8B3)*

*6510019 (ORC2L)*

*7000053 (QTRTD1)*

*2230048 (BC016235)*

*450167 (USH3A)*

*6130528 (SFRS10)*

*2190239 (CLSPN)*

*6980435 (DIXDC1)*

*6350528 (GRWD1)*

*5550288 (ORC4L)*

*430632 (N/A)*

*2810619 (NFE2L2)*

*2760008 (MAML1)*

*2940736 (BCAN)*

*4480167 (CSNK1D)*

*6040440 (SLCO1A4)*

*6040333 (MICAL3)*

*6770041 (FUNDC2)*

*3780154 (CLCN4-2)*

*2940594 (MS4A2)*

*4120301 (HIP2)*

*5670746 (CLDN1)*

*1230722 (1300010F03RIK)*

*4670273 (D11ERTD498E)*

*60064 (HADHB)*

*5900082 (SLC15A4)*

*2510184 (E430025L02RIK)*

*1980575 (NCOR2)*

*5360484 (EHD3)*

*1170670 (GCL)*

*70315 (STX16)*

*2340594 (OLFR399)*

*6900093 (4632408A20RIK)*

*6860156 (RBM10)*

*6420180 (4930479M11RIK)*

*1940504 (BC035291)*

*3710739 (KIF9)*

*4060673 (DMN)*

*4810097 (MUM1)*

*430463 (RALGDS)*

*3870154 (TCRA-V13.1)*

*6400463 (ZFP212)*

*1660059 (AY078069)*

*4070082 (ASS1)*

*6420292 (OLFR1107)*

*2450400 (2010004B12RIK)*

*3140731 (TSSC4)*

*2360373 (BC011209)*

*2060102 (BC049354)*

*2260576 (2310061A22RIK)*

*3060273 (STX1A)*

*1940180 (CKLFSF3)*

*2810072 (AQP7)*

*2350594 (PLAC1)*

*3520112 (6720463E02RIK)*

*2680440 (0610037M15RIK)*

*6180377 (ADAR)*

*6450288 (OLFR1309)*

*2360398 (ALDOA-PS2)*

*2230170 (6820443O06RIK)*

*2060390 (ING3)*

*6110338 (ABCB9)*

*2370072 (LRPPRC)*

*580050 (PSMC4)*

*580091 (MYOM1)*

*2370441 (NCF2)*

*4730092 (PLA2G7)*

*2320020 (TEMT)*

*4670300 (PDIR)*

*1190309 (CYP7B1)*

*5890551 (NUDT15)*

*2230471 (1700031F13RIK)*

*6350373 (6330416L11RIK)*

*3290440 (D11ERTD498E)*

*2370435 (8030491N06RIK)*

*4210441 (PAIP2)*

*730148 (D3JFR1)*

*7050279 (H2-M10.6)*

*3800315 (6330548G22RIK)*

*5550427 (BNC1)*

*6380541 (CASP1)*

*4120288 (2310061G07RIK)*

*6200324 (4921509C19RIK)*

*4070132 (2310011J03RIK)*

*2450603 (PDLIM2)*

*5420204 (1700024G13RIK)*

*6660059 (2810039F03RIK)*

*50605 (A630055G03RIK)*

*2810193 (TBP)*

*4560286 (NPHP1)*

*630270 (IGSF1)*

*6040270 (PAX2)*

*5290348 (1110004B15RIK)*

*6100022 (ZFP386)*

*4610458 (KIF18A)*

*6660040 (9030411M15RIK)*

*450152 (F730014I05RIK)*

*2060722 (SOS2)*

*4060441 (2310004N11RIK)*

*5900647 (MIZ1)*

*7040041 (HINT3)*

*3190014 (RABGAP1L)*

*4120592 (S100A5)*

*6590347 (1110028E10RIK)*

*6900132 (4632425D07RIK)*

*2030025 (OLFR323)*

*2640433 (A730098P15)*

*2030411 (A630085K21)*

*5720541 (2610018I05RIK)*

*3390167 (ELA1)*

*2260369 (OLFR1360)*

*6370093 (RDH1)*

*7000168 (E330018D03RIK)*

*3140551 (4921529N20RIK)*

*6200064 (CNTFR)*

*5290332 (BC003332)*

*2340438 (MELA)*

*540520 (4933434L15RIK)*

*450100 (PDLIM1)*

*610056 (AGPAT1)*

*4050750 (ANXA10)*

*380066 (GZMB)*

*4050273 (AA881470)*

*630132 (OLFR883)*

*2190450 (HIST1H4I)*

*2810162 (PHKA1)*

*5900093 (XRN1)*

*520050 (4931440B09RIK)*

*2360411 (SERPINB1C)*

*2360471 (EAR1)*

*2760692 (BCL2L2)*

*6450129 (N/A)*

*6020673 (LY6G6C)*

*4120132 (HTF9C)*

*6180180 (SLC30A4)*

*6110398 (HRB)*

*1580121 (COX11)*

*4120204 (TMPRSS4)*

*1230551 (NME7)*

*3130037 (2900006B13RIK)*

*6400725 (2310042M24RIK)*

*380619 (LOC268885)*

*6200181 (ZFP353)*

*6980017 (BC032204)*

*7040377 (FXYD2)*

*4730059 (SALL4)*

*6180088 (BC055107)*

*1050138 (2810022L02RIK)*

*1500161 (ANK3)*

*5890519 (PRKCN)*

*2760372 (DNAJB4)*

*2190315 (A530064D06RIK)*

*1660026 (MUC19)*

*5690035 (CREBBP)*

*5910435 (OLFR100)*

*2340121 (ASPH)*

*1980332 (1110014J01RIK)*

*520450 (GMPPA)*

*2370575 (XLR4)*

*940102 (ARHGEF19)*

*670368 (INPP1)*

*4060433 (OBOX2)*

*5550286 (9030407H20RIK)*

*4590348 (RGS19IP1)*

*6650463 (BAMBI)*

*6380440 (OLFR1339)*

*2370707 (F730047E07RIK)*

*2260440 (LZTS2)*

*1050059 (NRG2)*

*580315 (A230065J02RIK)*

*6900711 (6330406L22RIK)*

*520463 (PTTG1)*

*4850072 (TAS1R2)*

*6860082 (SNED1)*

*4920056 (SRCASM)*

*3440121 (5033406L14RIK)*

*6770278 (STX6)*

*4280167 (SLC5A3)*

*3360427 (PLOD2)*

*2060706 (DOC2G)*

*1410138 (RARB)*

*430026 (DST)*

*6370128 (ANXA11)*

*2640551 (GPR18)*

*6350148 (OLFR1223)*

*4730164 (ACTR8)*

*4070026 (MAT2A)*

*4480575 (BC025600)*

*5720142 (MAN2A2)*

*1340114 (RIPK5)*

*3800113 (L3MBTL3)*

*4200619 (EDG1)*

*3360086 (ELAVL4)*

*1660239 (OLFR1085)*

*2190176 (FRMD4A)*

*2680450 (C330027C09RIK)*

*3520286 (D10ERTD438E)*

*6100204 (PLA1A)*

*940239 (TUBA4)*

*2470064 (5830472M02RIK)*

*1400184 (OLFR1203)*

*3190735 (IMPDH1)*

*5890204 (PRC1)*

*2340706 (TRPM8)*

*6350181 (D330005C11RIK)*

*4730167 (2610511M17RIK)*

*6860368 (METTL2)*

*1850110 (UBE2S)*

*6220022 (STX1B2)*

*3450576 (NIN)*

*5390672 (AOAH)*

*2630088 (2310056K19RIK)*

*4610324 (MAOA)*

*5700364 (2600011C06RIK)*

*4050152 (GNAS)*

*6020136 (4933434I20RIK)*

*3360750 (GLIPR1)*

*5910097 (NUDT7)*

*6200725 (1110014P06RIK)*

*6760097 (OLFR846)*

*3130066 (LRPB7)*

*4200253 (SLC22A17)*

*430731 (NEK4)*

*5910563 (NARG1)*

*6450088 (UBE3B)*

*6110403 (SNF1LK)*

*6420541 (SLC18A2)*

*2450273 (AA407659)*

*3940301 (IL1RAP)*

*3450372 (CIDEC)*

*520348 (AOC2)*

*6350300 (GDI3)*

*6450097 (1700061J05RIK)*

*4060593 (4921528I01RIK)*

*1500484 (PIGH)*

*2970497 (SFRS14)*

*5270402 (4930573I19RIK)*

*4070156 (PLXNA4)*

*130632 (HIST1H1T)*

*6380242 (TENS1)*

*7000156 (TCF12)*

*6100452 (NHLH1)*

*5890670 (CRISP2)*

*6130411 (PRKAR1B)*

*2690176 (ATF7IP)*

*6220075 (ITPKA)*

*5670541 (SLC22A19)*

*380592 (5730478M09RIK)*

*4280398 (RHO)*

*450093 (2310039L15RIK)*

*5360685 (OTOP3)*

*6290435 (2810489O06RIK)*

*6400332 (1500005J14RIK)*

*6980008 (ZFP87)*

*2100162 (OAS1D)*

*450082 (SLC22A1)*

*3780162 (DNB5)*

*4120040 (PHKA1)*

*6110161 (OLFR811)*

*5910603 (A230097C02)*

*6660497 (ESD)*

*6220129 (PIK3CA)*

*5900014 (REC8L1)*

*130601 (PALM)*

*130333 (5730407K14RIK)*

*6940020 (USP40)*

*6290121 (IRF1)*

*380692 (H2-T23)*

*6180072 (PANX3)*

*940129 (GCNT1)*

*4920372 (CAR11)*

*5340095 (C330018J07RIK)*

*1190390 (2810423E13RIK)*

*1230195 (9130023D20RIK)*

*6180403 (PCNA)*

*3190008 (RAD51L3)*

*2360176 (MPO)*

*3060037 (EXOC7)*

*3780037 (ZFP503)*

*450025 (D8ERTD812E)*

*130129 (3322402L07RIK)*

*1190358 (TNFRSF19L)*

*2690563 (HRC)*

*2650435 (C030019F02RIK)*

*3780086 (COL12A1)*

*70044 (V1RH17)*

*6590092 (BLNK)*

*3610017 (A330080J22RIK)*

*4060113 (KCNJ14)*

*450364 (ADCY6)*

*1050014 (MTMR3)*

*1050528 (4930526H21RIK)*

*1940112 (CAMK2A)*

*4670315 (FBXL14)*

*5570133 (OLFR652)*

*4280397 (LCP1)*

*4560292 (4930558O21RIK)*

*6550348 (AW049765)*

*4060242 (BC031593)*

*3450632 (DGCR8)*

*6220358 (DEXI)*

*6370520 (ADORA1)*

*4780129 (RBM6)*

*4120138 (GTF2A1)*

*3870048 (TCFAP2B)*

*1940600 (HIST1H4A)*

*3780575 (5330438D12RIK)*

*6290100 (ADH5)*

*6220064 (1810057C19RIK)*

*3060139 (ADD1)*

*5050411 (OLFR262)*

*7000195 (TAC1)*

*1770167 (SELPL)*

*1660040 (TAS2R113)*

*2970725 (CYP2S1)*

*70372 (LINCR)*

*2650010 (PEX13)*

*2030377 (BC032925)*

*6370519 (COX4I2)*

*1980692 (SLC12A5)*

*6550463 (CHGA)*

*2760373 (SRST)*

*4010494 (POLR3F)*

*5860301 (ZFP28)*

*4730519 (C030039L03RIK)*

*450524 (N/A)*

*5550088 (6430527G18RIK)*

*70176 (KCNJ15)*

*70575 (TEX11)*

*2340528 (CACH)*

*4210025 (BC052328)*

*3060433 (TXNDC5)*

*2970102 (D13WSU50E)*

*5360577 (D10WSU102E)*

*3520465 (CBLB)*

*450411 (POSTN)*

*50273 (5830427D03RIK)*

*6370086 (PBX4)*

*540435 (CCL25)*

*4070671 (4632428N05RIK)*

*4120072 (P42POP)*

*3520300 (PPP1R16B)*

*4230176 (1810059A23RIK)*

*1690152 (PAK4)*

*4060520 (BC016608)*

*1340059 (SLC38A4)*

*2350400 (CIB3)*

*1190446 (SPA17)*

*4540059 (BMX)*

*5890632 (SIRT7)*

*4070239 (PRG)*

*1740156 (ATP4B)*

*4480458 (1110007A06RIK)*

*3450735 (1700049L16RIK)*

*4670091 (SSPN)*

*3060102 (FBXW17)*

*1980368 (MYBPC2)*

*6220400 (ZFP307)*

*1170309 (SIAH1B)*

*3840059 (ARL8)*

*2360577 (1110006I15RIK)*

*2260500 (GALNT5)*

*1980364 (4932423M01RIK)*

*5720369 (DPP4)*

*3840594 (XBP1)*

*4230369 (AI467606)*

*6770471 (2900006B13RIK)*

*6450408 (POMC1)*

*6450114 (1110067L22RIK)*

*2630279 (N/A)*

*1410400 (PKD1L1)*

*3190504 (ELF2)*

*6940102 (CDC25B)*

*3830458 (1700086L19RIK)*

*1740110 (V1RC21)*

*1450039 (SV2A)*

*3830369 (SPIN)*

*540377 (0710005I19RIK)*

*2340102 (BC021395)*

*870253 (CNTF)*

*1780164 (ANKRD23)*

*4760601 (IVNS1ABP)*

*6650092 (C1RL)*

*6660273 (B230365F16RIK)*

*3170035 (A930005I04RIK)*

*6180215 (AMIGO)*

*3850059 (6430571L13RIK)*

*2350427 (1300006N24RIK)*

*5340132 (A930008K15RIK)*

*460433 (1190002J23RIK)*

*1190100 (1600013P15RIK)*

*130487 (CHI3L4)*

*4060446 (HIST1H4H)*

*5360707 (2010309E21RIK)*

*1740373 (NEDD9)*

*1690551 (ARID1A)*

*1580593 (SEPTIN 4)*

*1450132 (RNF8)*

*1230092 (2900006B13RIK)*

*6450750 (1700040I03RIK)*

*2810056 (LSAMP)*

*5390377 (SCNN1B)*

*4060670 (CLIC3)*

*4560014 (EDARADD)*

*3170075 (PCSK4)*

*3450168 (9030227G01RIK)*

*2760044 (4921530D09RIK)*

*6860441 (KCNQ1)*

*2350373 (OLFR876)*

*4560706 (H60)*

*2340292 (RGS10)*

*5890309 (4933434H11RIK)*

*2450576 (C230052I12RIK)*

*1660253 (ZFP574)*

*6220152 (CELSR2)*

*5290064 (SLC1A4)*

*4200017 (H2-Q6)*

*5720056 (MUCDHL)*

*6450176 (E030003N15RIK)*

*540671 (A930040G15RIK)*

*510039 (9530090G24RIK)*

*5360026 (COPEB)*

*3440092 (1810018L02RIK)*

*4670497 (V1RG2)*

*630047 (PIM1)*

*4670400 (LRRC4B)*

*510154 (2610024N01RIK)*

*3360520 (LIX1)*

*70368 (E130119H09RIK)*

*4120541 (PRDM16)*

*6450528 (N/A)*

*6200072 (9030625G08RIK)*

*3120324 (2900054P12RIK)*

*4480184 (CCR4)*

*2360446 (GPHB5)*

*5080156 (ITGB1)*

*1740026 (GJA5)*

*3120014 (ASB18)*

*5420670 (D130038B21RIK)*

*7050484 (MAPK12)*

*6550010 (FBLN5)*

*2690100 (V2R8)*

*6400707 (4933408F15)*

*2360082 (OLFR676)*

*6130451 (YY1)*

*1850154 (CDCA1)*

*540619 (D15ERTD621E)*

*3120452 (1700022C21RIK)*

*3120132 (ATP6V1G1)*

*4060068 (TMPRSS5)*

*6420142 (C030002J06RIK)*

*4730154 (ZFP189)*

*4070059 (DNMT3B)*

*1400739 (CD28)*

*6980181 (TUBA3)*

*4670010 (AI844366)*

*6350593 (CSK)*

*4060253 (N28178)*

*3830411 (5830457O10RIK)*

*940035 (PSMB2)*

*2340685 (IL10)*

*60152 (AGPAT3)*

*6940746 (B230339H12RIK)*

*5340504 (COVA1)*

*2320156 (0610037P05RIK)*

*7000056 (SDHA)*

*1500731 (ZFYVE28)*

*6180736 (ADAM26)*

*1190504 (INSR)*

*6420546 (E230026N22RIK)*

*3140484 (4933417C16RIK)*

*2570128 (SDHD)*

*6860273 (ATR)*

*5130528 (DSCR6)*

*3520372 (2810003C17RIK)*

*4670451 (D430018P08)*

*6420184 (PPP1R14C)*

*1980725 (RAB37)*

*3610270 (9230105K17RIK)*

*3940711 (ABCA4)*

*1660605 (CST8)*

*4850528 (OLFR639)*

*1990722 (ATP5A1)*

*380239 (CFL2)*

*580605 (1700012B15RIK)*

*2680647 (MCM5)*

*510398 (PHLDA3)*

*4280735 (NT5C2)*

*7050129 (FBXO9)*

*2030170 (MOSPD1)*

*2810603 (AI505034)*

*6130672 (BRD3)*

*3870711 (ZFP289)*

*1580035 (D2ERTD435E)*

*4540091 (IL5RA)*

*6020181 (DNAJC2)*

*2480497 (OLFR945)*

*6040609 (2810452K22RIK)*

*460494 (ELK4)*

*4120280 (D230019K20RIK)*

*6130193 (CYP2C54)*

*870497 (RHBDL7)*

*380152 (POLR3E)*

*5670619 (AI481214)*

*5050114 (FABP4)*

*2260039 (ZFPN1A1)*

*6550059 (SYTL3)*

*870161 (PCYOX1)*

*5130717 (TOR3A)*

*1190079 (EIF3S10)*

*70541 (AI326906)*

*780176 (CLCN3)*

*7000048 (5730494M16RIK)*

*4280097 (CENPH)*

*3610575 (PAOX)*

*1660452 (BCOR)*

*6380528 (MYCS)*

*3450403 (4632419I22RIK)*

*3060722 (SLC5A5)*

*7000017 (BC008163)*

*520068 (NRM)*

*2100100 (VAMP2)*

*2260670 (CHRND)*

*2350348 (HPCAL1)*

*6420731 (2410015A16RIK)*

*1690403 (9430038I01RIK)*

*3870484 (UBQLN1)*

*4780086 (ABTB1)*

*4560671 (AHR)*

*450451 (OLFR1030)*

*380373 (1700055D18RIK)*

*7000278 (FABP6)*

*6380398 (DPM1)*

*2480010 (USP48)*

*4010739 (IFITM6)*

*3290075 (A430107O13RIK)*

*2260601 (NCB5OR)*

*4730739 (E330008O22RIK)*

*6180594 (0610009B22RIK)*

*6400019 (DMRT1)*

*6370017 (TRERF1)*

*4560619 (4933436E20RIK)*

*2510019 (6430548M08RIK)*

*6770110 (LEPRE1)*

*6180397 (MTRR)*

*2510373 (BC030045)*

*2350044 (HMGB1)*

*4850494 (5031414D18RIK)*

*4150204 (PARP8)*

*4210102 (ARMC8)*

*50672 (5830483C08RIK)*

*450040 (1700030K01RIK)*

*2350075 (GPD2)*

*3440133 (ETHE1)*

*430717 (FASN)*

*6400026 (SP2)*

*6840576 (DSC3)*

*5570605 (MAP1LC3A)*

*3360154 (PINX1)*

*4210091 (2600001J17RIK)*

*6100528 (PTPN11)*

*5390072 (HMX1)*

*5270239 (FBF1)*

*2650397 (SULT2A2)*

*2340605 (MTRF1)*

*3290538 (MC3R)*

*6380162 (MAP3K5)*

*6200041 (MDFI)*

*4050563 (1810010N17RIK)*

*510136 (NME6)*

*5270673 (EPB4.1L2)*

*6840072 (IKBKB)*

*2230433 (CNN2)*

*3360692 (1110061N23RIK)*

*4920528 (TRAF4)*

*6200504 (INPP4B)*

*1500736 (RHBDL)*

*4480538 (RTN2)*

*4060541 (IDUA)*

*7000142 (HSD17B7)*

*3610338 (5730457F11RIK)*

*450402 (MAST3)*

*3850692 (OXT)*

*5360035 (LRP8)*

*2340035 (CORO2B)*

*3830750 (ZFP191)*

*4060348 (HIST1H4J)*

*4590064 (CAPZA2)*

*2320438 (DLX2)*

*1410170 (BC028799)*

*5360438 (TICAM2)*

*6370068 (CXADR)*

*3440164 (SMC5L1)*

*2810504 (NOL6)*

*2690148 (SLCO6B1)*

*6510050 (NCK1)*

*7100133 (6330578E17RIK)*

*4670446 (OLFR1289)*

*1500008 (WDR36)*

*2630037 (NRARP)*

*6420524 (ADRM1)*

*6040672 (SLC12A2)*

*2850576 (1110051B16RIK)*

*6370019 (CNTN4)*

*3360735 (NMB)*

*4210435 (OLFR192)*

*3940440 (OOG3)*

*1690438 (ZFP287)*

*3870427 (MS4A6C)*

*1980088 (5730509C05RIK)*

*3450091 (4930565A21RIK)*

*3870167 (DOK3)*

*60577 (CASP9)*

*3840047 (2300005B03RIK)*

*4150037 (TDE2L)*

*360148 (STK17B)*

*3830601 (DMGDH)*

*2850044 (NEDD1)*

*50722 (4921528H16RIK)*

*4210520 (MGLL)*

*5670242 (D330001F17RIK)*

*580358 (1700055O19RIK)*

*70338 (CARD11)*

*2370097 (NUP37)*

*6450471 (DRPLA)*

*5550114 (1700012H17RIK)*

*2970537 (LOC330599)*

*6650114 (OLFR608)*

*3390278 (ACIN1)*

*2360161 (IFITM5)*

*2480717 (DXIMX39E)*

*7000368 (EN2)*

*6180184 (SDCCAG1)*

*4210632 (ITGAE)*

*1500685 (CCNG1)*

*1400273 (ASCL2)*

*1740520 (SLC35D1)*

*6550184 (LTBP1)*

*4920014 (ZFP322A)*

*4610369 (4931417A20)*

*6180333 (ZFP292)*

*1990500 (TSLP)*

*3140390 (CAPZA1)*

*5390369 (RGS12)*

*5290402 (1110001A05RIK)*

*5550176 (DCLRE1B)*

*5670286 (RPEL1)*

*1580091 (TDRKH)*

*6100161 (1110001A05RIK)*

*1690373 (4932416A11RIK)*

*4590722 (TMOD4)*

*1050292 (A930021H16RIK)*

*2650593 (FARSLA)*

*3990400 (OLFR1259)*

*6770333 (RAI2)*

*3060465 (HYOU1)*

*4610021 (RPP40)*

*360603 (SLC4A3)*

*6770044 (EIF3S1)*

*4920136 (PRH1)*

*5360541 (HABP2)*

*3830286 (BC024502)*

*6200131 (CBFA2T2H)*

*70280 (BB219290)*

*3360292 (SDPR)*

*3190037 (CORO1A)*

*6450500 (1700024C24RIK)*

*7100048 (MBNL1)*

*6290397 (EWSH)*

*580440 (CRYBA1)*

*2690372 (KCNK3)*

*3170184 (RNH2)*

*1770164 (IL10RA)*

*6860112 (LAG3)*

*1240020 (MKNK2)*

*110035 (RNF31)*

*2940746 (SRRM2)*

*1570086 (2810022L02RIK)*

*770039 (EEF2)*

*450601 (9030607L17RIK)*

*450494 (ZMYM1)*

*6400044 (ANKRD17)*

*6900128 (PMFBP1)*

*6590017 (SP1)*

*6370398 (SLC15A1)*

*3850685 (RAB33B)*

*2370161 (OLFR1122)*

*2970670 (SLC35A5)*

*6420746 (PRIM1)*

*510056 (9630054F20RIK)*

*840215 (2610016F04RIK)*

*2360541 (D730046L02RIK)*

*6220286 (5230400J09RIK)*

*460348 (NDUFB4)*

*5360168 (EN1)*

*3840463 (RAB6)*

*2060671 (1200016D23RIK)*

*1690605 (SLC6A2)*

*1190242 (HOOK3)*

*3830687 (2900026H06RIK)*

*4730722 (N/A)*

*870537 (PITX2)*

*3710451 (NALP2)*

*5270368 (0610041E09RIK)*

*360086 (R74862)*

*4200546 (A930029B02RIK)*

*2100504 (6720469N11RIK)*

*3850576 (TIGD3)*

*1190731 (SIRT1)*

*380044 (PINK1)*

*2690520 (NCOA6IP)*

*7100440 (HERPUD1)*

*3990390 (RAD9)*

*3800102 (CBR2)*

*2030576 (4932418K24RIK)*

*3450731 (INPP5D)*

*6040132 (AARD)*

*5290053 (OLFR247)*

*3170056 (SNAP91)*

*4200301 (5730537H01RIK)*

*4150324 (ABCB7)*

*7050025 (EN1)*

*730685 (A830006F12RIK)*

*1940609 (OLFR871)*

*6590110 (ITGA11)*

*6290722 (CRY2)*

*2190463 (BC021608)*

*2030446 (MGLL)*

*2360717 (2010001E11RIK)*

*7050138 (MYL9)*

*4920538 (4931412G03RIK)*

*7040609 (YTHDF3)*

*6040603 (BC027088)*

*5340092 (CHRNA6)*

*3940487 (DISC1)*

*3190273 (MUG1)*

*1450619 (PCYT1A)*

*6550253 (2410012M04RIK)*

*5700093 (PPIL3)*

*2630494 (IRX5)*

*6840161 (MKL1)*

*730017 (LOXL2)*

*5700204 (MKL2)*

*5290035 (AF155546)*

*7000161 (MRPL39)*

*1240563 (MRGPRA2)*

*2810070 (APOB48R)*

*3940148 (VGLL2)*

*1400091 (SLC7A3)*

*3990139 (MTMR2)*

*3710079 (CTBP2)*

*6940128 (EGR3)*

*7050397 (BC022687)*

*4060707 (9430079M16RIK)*

*780142 (PET112L)*

*770273 (CD37)*

*3800075 (PPP1R14B)*

*360672 (DHX40)*

*2260086 (MAP2K7)*

*1850050 (CFHL1)*

*6020440 (PDCD11)*

*510072 (STFA2)*

*450377 (NQO3A2)*

*5550484 (A030013D21)*

*6450402 (1700007K09RIK)*

*6200095 (RGN)*

*1340605 (BRP44L)*

*4120750 (SCOSPONDIN)*

*2360053 (CRIP1)*

*1770735 (4921513H07RIK)*

*1230039 (FSCN2)*

*4010121 (A630005A06RIK)*

*2470053 (SLC5A1)*

*2230040 (FBXL12)*

*6380164 (CDX4)*

*2850132 (DTNA)*

*2510102 (HBP1)*

*3440504 (HDAC8)*

*5700368 (C130036J11)*

*4810075 (TXNL6)*

*5390022 (TCF1)*

*4070619 (KCNH1)*

*2060398 (B930018B01)*

*2650092 (BC042720)*

*5080064 (LIMK1)*

*4670026 (NPAT)*

*460286 (SSB)*

*4070465 (DDEF1)*

*2900181 (B930013M22RIK)*

*1770278 (ERF)*

*5860438 (IL15)*

*6200687 (1110006G14RIK)*

*1340075 (OLFR968)*

*1660398 (CTSJ)*

*1690092 (HOMER1)*

*510402 (KRT2-6A)*

*2320148 (CELSR2)*

*630093 (AMPD2)*

*6980341 (OLFR1487)*

*4230452 (TMEM34)*

*1170601 (FN1)*

*5860064 (0610011I04RIK)*

*2350593 (5830472H07RIK)*

*2940685 (SGCD)*

*450113 (HAAO)*

*6550131 (VLDLR)*

*6900452 (CRBN)*

*1660020 (C130096D04RIK)*

*580398 (HAPLN1)*

*2690687 (NVL)*

*870685 (2610040E16RIK)*

*1980138 (TCFAP2A)*

*1230142 (GMFG)*

*730300 (RASSF3)*

*2340113 (TERF1)*

*3450672 (9830147P19RIK)*

*4210619 (HBP1)*

*6760438 (GHRL)*

*2360180 (PIGT)*

*1850601 (MDH2)*

*3830066 (SRGAP2)*

*6760180 (NUP88)*

*6450204 (DSPG3)*

*2340309 (CRYGA)*

*2760014 (ADMR)*

*6650735 (ATP7B)*

*870133 (TM4SF2)*

*2760048 (2410014A08RIK)*

*3360619 (CYP2A12)*

*4760242 (SCN10A)*

*450019 (RAB27A)*

*1660092 (EMX2)*

*6110575 (GYLTL1B)*

*4210440 (FGF16)*

*3140100 (FTSJ3)*

*5390593 (TDPOZ3)*

*5690026 (NFIC)*

*4480022 (STATIP1)*

*3800129 (TTK)*

*5890156 (TCEB3)*

*4920358 (GNG8)*

*1980411 (1700006H02RIK)*

*6550072 (PMP22)*

*4570131 (GFI1B)*

*5570373 (LEPRE1)*

*6840273 (2610529H08RIK)*

*460215 (MATN2)*

*4120050 (KCNE1)*

*4280181 (2310047O13RIK)*

*2030152 (5430407P10RIK)*

*6980440 (LOC333669)*

*3870095 (RAI3)*

*3940546 (4931440F15RIK)*

*4210161 (B130050K08)*

*2510056 (1700081D17RIK)*

*2640195 (MAPK8)*

*450338 (AQP1)*

*3850338 (FMO2)*

*6450064 (KIST)*

*3610088 (2600001B17RIK)*

*6980348 (MFTC)*

*5900484 (IL18R1)*

*430563 (SLC5A4B)*

*5570193 (MAP2K3)*

*6550494 (CNR1)*

*510059 (KCNA3)*

*3060408 (BCAN)*

*4760739 (CYBA)*

*6980632 (OLFR549)*

*3840102 (2600016J21RIK)*

*1740324 (4930555G01RIK)*

*1850278 (MMP17)*

*6180066 (RBPSUH)*

*5700647 (FBXO34)*

*4280707 (AP1S2)*

*7000524 (ZFP114)*

*2340408 (CNGB3)*

*3140575 (RHPN2)*

*3290408 (PTPN9)*

*6550093 (RETNLG)*

*5550497 (NUDT3)*

*6450139 (NF2)*

*1690082 (N/A)*

*460301 (HELB)*

*3800603 (TMC4)*

*7050739 (MPPE1)*

*450735 (VAMP1)*

*3060100 (CHAD)*

*5700253 (9130221D24RIK)*

*1190035 (KIRREL1)*

*6650609 (A130052D22)*

*3440739 (H2-M1)*

*6860184 (EIF4G2)*

*5360332 (CCRL1)*

*1780358 (CDK8)*

*2810010 (ARHGAP1)*

*450537 (TXNDC4)*

*6200278 (VIAAT)*

*580397 (2410076I21RIK)*

*7050390 (TCFAP2D)*

*2350270 (GPT1)*

*1170270 (LOC381621)*

*5670086 (GPRC2A-RS5)*

*1500400 (0610041D19RIK)*

*2340402 (KRT1-14)*

*1990333 (MPZ)*

*2320519 (A330096I21RIK)*

*3710021 (OLFR1118)*

*2690278 (AY026312)*

*2690059 (2810429K17RIK)*

*3390484 (CREB3L2)*

*7100086 (2410004J23RIK)*

*2100091 (2900090M10RIK)*

*4010687 (RHOBTB3)*

*1230348 (A830007L07RIK)*

*5910288 (MMP1A)*

*2030400 (CNGA1)*

*4850332 (ZFP57)*

*460253 (1110013G13RIK)*

*1050156 (TAS2R106)*

*3360193 (1810034M08RIK)*

*4070687 (IFNAR2)*

*2630280 (HCR)*

*4610176 (SSX2IP)*

*460091 (ADAM23)*

*2470132 (TCTEX1)*

*3060672 (HSD3B1)*

*6040687 (RECQL)*

*2350731 (WFIKKN1)*

*1990288 (N/A)*

*6860068 (4930515G01RIK)*

*5690402 (IL28)*

*4610273 (HSF4)*

*1980441 (SCRN2)*

*1340195 (9930027N05RIK)*

*4780128 (CRK)*

*4780500 (ALG8)*

*2360102 (YME1L1)*

*1450139 (4933407H18RIK)*

*2360338 (ZFP339)*

*5550671 (D130079A08RIK)*

*5220497 (PIP5K2A)*

*770064 (NUMB)*

*1940044 (RIN3)*

*630538 (9230112O05RIK)*

*2030332 (OLFR275)*

*6350592 (CORO1B)*

*2360132 (TRIM17)*

*3830008 (BAX)*

*6200242 (XPNPEP1)*

*510692 (RAB8B)*

*4480368 (RG9MTD1)*

*380039 (NKTR)*

*6840398 (5031400M07RIK)*

*6590500 (LENG1)*

*1990162 (BC022146)*

*360451 (ETS2)*

*1690100 (LHX1)*

*6770736 (FRMD4B)*

*1740563 (ABI3)*

*1410575 (DUOX2)*

*6380041 (1700128F08RIK)*

*2120215 (MISC12)*

*4050300 (HTR1D)*

*730504 (MLR2)*

*1340162 (QTRT1)*

*4200075 (OLFR211)*

*2120154 (KHDRBS3)*

*1770064 (ITIH5)*

*2510242 (HDAC11)*

*6660152 (MINA)*

*1780154 (N/A)*

*6840632 (1700029I15RIK)*

*1450524 (CKM)*

*6620397 (CASP3)*

*3060280 (1110002B05RIK)*

*3870270 (CDH2)*

*1580600 (2310016N21RIK)*

*2510164 (AW547365)*

*2640685 (EIF3S6)*

*4050739 (RP9H)*

*2350563 (AW551984)*

*6510086 (COL6A1)*

*770324 (GYK)*

*2970180 (PEX7)*

*3290463 (EIF2B3)*

*4280088 (NR2F6)*

*1770092 (DFFB)*

*2370672 (APOB)*

*6860070 (GPHA2)*

*1570180 (MUC10)*

*5360121 (FGF13)*

*3450079 (2610024G14RIK)*

*3120176 (PDCD5)*

*5570338 (KRT2-8)*

*2360079 (TUBE1)*

*50180 (SQLE)*

*3130193 (TYRO3)*

*5720168 (PFKM)*

*3450563 (CD79A)*

*3290056 (1810049H13RIK)*

*4230301 (TDRD1)*

*60170 (NME7)*

*5290500 (POLR3K)*

*4230309 (XRN2)*

*510369 (KPNA4)*

*5670711 (ARNT)*

*4050156 (TNRC4)*

*6400112 (LOC380800)*

*7100438 (1110011F09RIK)*

*4070576 (SVIL)*

*5420735 (SLC7A9)*

*450148 (PIT1)*

*4540731 (4930577M16RIK)*

*840707 (ARG1)*

*6100014 (MGEA5)*

*6020300 (B3GALT1)*

*360402 (NR2F1)*

*7100204 (MTAP1B)*

*2640132 (D2ERTD750E)*

*4010142 (GNG11)*

*6660035 (LIG4)*

*2470242 (OLFR920)*

*5360369 (NEK11)*

*4570292 (ART1)*

*2640112 (5330439J01RIK)*

*5130253 (NNAT)*

*1770193 (ZFP113)*

*1850279 (ITPA)*

*3440161 (LOC380803)*

*2120008 (HNRPUL1)*

*460373 (1110057K04RIK)*

*5340746 (ELOVL6)*

*4610050 (D5ERTD689E)*

*4280725 (OLFR786)*

*5690167 (ABAT)*

*1780605 (4432411E13RIK)*

*5420164 (FRMPD1)*

*4610184 (4930431B11RIK)*

*1190471 (OTX2)*

*6020128 (2410017I18RIK)*

*3450215 (4933440M02RIK)*

*2630180 (SERPINA1E)*

*4210270 (VARS2L)*

*3800253 (NUMB)*

*460408 (NRXN1)*

*4480242 (9330175B10RIK)*

*2810332 (4833413G10RIK)*

*4050139 (PLTP)*

*6860435 (TAF5)*

*2370309 (RBBP7)*

*6450315 (OLFR1402)*

*6350131 (NALP5)*

*4070452 (FZD5)*

*940072 (HBS1L)*

*4060372 (ESR1)*

*5700035 (MAD1L1)*

*5270161 (SULT4A1)*

*3120494 (5830493J20RIK)*

*3780750 (GRIA1)*

*2190706 (FGF18)*

*2340603 (2900006N09RIK)*

*3710446 (ARF3)*

*4280347 (GCDH)*

*2320100 (9930021J17RIK)*

*6770600 (NCOA4)*

*1990528 (9330161C17RIK)*

*2970538 (9030203C11RIK)*

*7100592 (FSHB)*

*1400528 (PIGO)*

*670195 (OLFR1449)*

*4560161 (4732496O08RIK)*

*4050538 (ZFP455)*

*6180075 (CPT2)*

*2030487 (SPHK2)*

*4210148 (UCP3)*

*6860131 (FBXO3)*

*4610180 (SS18L1)*

*6510181 (SFTPD)*

*7050020 (ASF1A)*

*2190324 (GCDH)*

*6450600 (CSNK1D)*

*2690154 (GPSM3)*

*2900551 (4930579A11RIK)*

*50576 (PLCB4)*

*6220095 (GALNT9)*

*3290010 (4930565A21RIK)*

*2260040 (CATSPER3)*

*6770161 (2310003L06RIK)*

*4610605 (VSX1)*

*4560446 (PSG16)*

*3390377 (MCF2L)*

*4590300 (BC023829)*

*2350072 (SMPDL3B)*

*3780091 (OLFR630)*

*5890368 (PSMD9)*

*1980433 (UPF3B)*

*2760110 (A730016F12RIK)*

*5050647 (TM4SF7)*

*3800022 (ADAM1A)*

*4230180 (BCR)*

*1850551 (CBFB)*

*2030671 (USP16)*

*5890102 (ZC3HDC3)*

*2690402 (HOOK1)*

*4280093 (2600011C06RIK)*

*6180338 (0610039K22RIK)*

*3940450 (GRASP)*

*130008 (PLXNB3)*

*60132 (EIF4EBP1)*

*4230170 (HSD3B2)*

*6420373 (ADRA2B)*

*840673 (D19ERTD386E)*

*360369 (2410091C18RIK)*

*5290647 (9530098N22RIK)*

*430647 (AGC1)*

*6620152 (2310031A18RIK)*

*6200600 (SH3RF2)*

*5130064 (LRP6)*

*3780082 (BC016495)*

*6400215 (SLC39A8)*

*5290047 (NEXN)*

*580736 (D330027H18RIK)*

*2940309 (DUSP7)*

*6760050 (SEC22L3)*

*2260056 (GIF)*

*4760136 (KCNMB2)*

*2650463 (NLGN3)*

*1170484 (BC020184)*

*610403 (GNG5)*

*2940193 (LILRB4)*

*6130647 (ZFP78)*

*3800377 (BC023957)*

*3610500 (0610005K03RIK)*

*5360647 (SDCCAG1)*

*6350301 (TCAM1)*

*2060091 (PSD2)*

*460142 (FBXO25)*

*6180315 (NMBR)*

*6380373 (MPST)*

*3120725 (SNCG)*

*3130372 (RBL1)*

*3440427 (TPK1)*

*4230086 (V1RI10)*

*1940435 (PIGA)*

*4150270 (CLDN15)*

*6650593 (PLEKHA5)*

*2340059 (PPT2)*

*5340113 (GPRK2L)*

*430164 (RENT1)*

*4150731 (NKX2-2)*

*2360309 (LIPH)*

*610593 (9330140K16RIK)*

*2360458 (C80913)*

*3870600 (OLFR1443)*

*6420021 (SH3YL1)*

*4010152 (TRP53RK)*

*2760086 (A230067G21RIK)*

*6180025 (TPMT)*

*1850086 (RNF30)*

*2340403 (TESP2)*

*6520148 (BC016188)*

*5420398 (CHD9)*

*4610070 (DOCK8)*

*3870309 (TGIFX1)*

*4200427 (8030498B09RIK)*

*3290100 (9130427A09RIK)*

*6450563 (BAK1)*

*2360136 (EPB4.9)*

*6200047 (SEC23IP)*

*6040647 (CHAF1A)*

*6900022 (ZFP13)*

*2640735 (GUCY1A3)*

*5270519 (BCCIP)*

*3290280 (IGF1)*

*6860750 (9430022F06RIK)*

*2320204 (NXF)*

*7050204 (EPS8)*

*5670368 (LRFN4)*

*2630398 (4122402O22RIK)*

*6420750 (9330152L17)*

*3850593 (1110055L24RIK)*

*2760102 (LTB4DH)*

*1690025 (RRM2B)*

*2470040 (E130010M05RIK)*

*430072 (HIST1H2AF)*

*6020600 (ACBD5)*

*4120402 (PCDHGC5)*

*4120161 (D7WSU128E)*

*6650471 (TPM3)*

*1740095 (N/A)*

*2340725 (NELL2)*

*1780181 (PFTK1)*

*7100348 (TRPV2)*

*2850440 (LY64)*

*4280373 (EIF3S8)*

*6350239 (4922501C03RIK)*

*6620594 (SRPX2)*

*7000132 (KMO)*

*4570280 (OLFR76)*

*580408 (LOC73072)*

*2370519 (D930036B08RIK)*

*3450037 (CYP11A1)*

*2850725 (NCOA2)*

*2690494 (OLFR54)*

*3360358 (AXIN)*

*4200739 (PET112L)*

*1410239 (OLFR270)*

*6900441 (4921517D22RIK)*

*4120097 (CTDP1)*

*5690333 (HSD3B2)*

*450180 (CSRP3)*

*3990278 (GALNT14)*

*1230725 (EIF4EBP3)*

*610519 (8430411H09RIK)*

*7000292 (TCFE3)*

*2680193 (SCGF)*

*6650372 (SAMD7)*

*2850537 (C230090D14)*

*5570156 (TRIM3)*

*3140524 (SC4MOL)*

*6200288 (SPAG5)*

*1190138 (FNBP3)*

*4200487 (BAG3)*

*110242 (SDS)*

*4670735 (EEF1A1)*

*6400056 (STXBP6)*

*1410739 (2810439M11RIK)*

*6980717 (1700040N02RIK)*

*2030601 (POU2F3)*

*2320044 (CRYZL1)*

*430441 (CHRNB3)*

*5290161 (LOC232060)*

*2810075 (PKNOX2)*

*1940301 (2610044O15RIK)*

*7100332 (OLFR178)*

*6200441 (DAB2IP)*

*610397 (PCDHB22)*

*1690497 (2010008E23RIK)*

*2350139 (LOC384402)*

*5910369 (C76566)*

*4590706 (4933415L06RIK)*

*5360022 (DXIMX39E)*

*6020398 (OLFR1052)*

*2190471 (3526402H21RIK)*

*4670215 (CDKN2A)*

*2260114 (N/A)*

*2360348 (1200008A14RIK)*

*3800438 (KCNQ4)*

*2350390 (4732486J07RIK)*

*2260711 (A030007L17RIK)*

*1570520 (CDS2)*

*4920280 (NRP)*

*2350315 (KCNJ4)*

*1400706 (2810441C07RIK)*

*2940338 (VEGFA)*

*360673 (B230315F11RIK)*

*6840139 (CUGBP2)*

*1450133 (LRRN3)*

*1500025 (OSR1)*

*1690390 (PARP16)*

*2360047 (PCDH8)*

*7040048 (9030623N16RIK)*

*4230446 (SDF2L1)*

*5700008 (UBADC1)*

*3060463 (OLFR473)*

*1230504 (UBE2R2)*

*3830746 (AI194308)*

*1850079 (BAT9)*

*4670332 (MRE11A)*

*3520725 (ABCF1)*

*2640068 (BXDC1)*

*4810601 (UGT1A9)*

*2360270 (7420402K12RIK)*

*2260180 (MBD1)*

*5550601 (4930511A21RIK)*

*50609 (PSMA6)*

*2340458 (CML2)*

*4210504 (C130099A20RIK)*

*3850373 (PTGFR)*

*3170113 (PPGB)*

*4120494 (PPAP2A)*

*2640670 (AI842396)*

*5270358 (SLC3A2)*

*1690465 (RRM2B)*

*5360594 (COVA1)*

*6520347 (AA987161)*

*460093 (SPRY1)*

*3610722 (CALN1)*

*6620301 (VIT)*

*1980369 (POLR3H)*

*6980731 (D130052B06RIK)*

*2510215 (OTX1)*

*6650162 (P2RX4)*

*6900092 (HIST1H4M)*

*6180717 (SGSH)*

*4670184 (CCDC9)*

*1570156 (1810055E12RIK)*

*5290215 (2310067B10RIK)*

*3170136 (CASQ2)*

*5570102 (B230220B15RIK)*

*580138 (AMY2)*

*3870278 (BC018399)*

*6200021 (NHLH2)*

*2450538 (4732429I09RIK)*

*6400672 (C330016K18RIK)*

*1570601 (OLFR790)*

*2760364 (RASSF1)*

*4560050 (TOP1MT)*

*2030114 (6430559E15RIK)*

*5910537 (N/A)*

*2190711 (MYBPH)*

*6100068 (MLLT1)*

*6220273 (PLXNB1)*

*3780593 (SESTD1)*

*6020133 (CEACAM2)*

*1340372 (NRIP3)*

*520037 (ADAM30)*

*840592 (5730507A09RIK)*

*6450110 (NCOA6)*

*3850632 (HIVEP1)*

*1690440 (FYB)*

*2350592 (EPN3)*

*430594 (FSIP2)*

*1090746 (2600005C20RIK)*

*6770152 (AGRN)*

*2100129 (4933440J22RIK)*

*3830402 (SIX3)*

*2810131 (FRZB)*

*1940075 (IL1RAPL2)*

*430176 (PPFIA1)*

*510035 (GRB14)*

*6110070 (DSCR1L2)*

*4010471 (FANCG)*

*6130358 (C630015F21RIK)*

*1410397 (SFPI1)*

*4010593 (ATIC)*

*6040070 (PTP4A2)*

*360048 (LY78)*

*3060110 (IGFBP7)*

*6100538 (TBL2)*

*2320500 (CLDN8)*

*2480095 (GPD1)*

*5570279 (CDC26)*

*6290450 (AOX1)*

*1240458 (1700020A23RIK)*

*3850168 (IL16)*

*6420093 (ALDOA-PS1)*

*3940735 (DNAJB4)*

*1940400 (4933440H19RIK)*

*2900014 (PPP2R2A)*

*6620551 (2810406C15RIK)*

*4070162 (2810453L12RIK)*

*6290341 (CAR7)*

*2650039 (STRA13)*

*4560181 (A730024F05RIK)*

*7000438 (PCDH15)*

*6660088 (SPNB3)*

*2350288 (IGSF11)*

*2340451 (GTF3A)*

*6400603 (RRAGA)*

*2900129 (C6)*

*6380176 (1810037K07RIK)*

*2640102 (LOC382137)*

*870242 (ATAD2)*

*3190593 (CDC23)*

*6130315 (3830408G10RIK)*

*2230097 (ARHGEF10)*

*4760707 (6530406M24RIK)*

*3940066 (TBXA2R)*

*7050132 (ACTN4)*

*70358 (TACR1)*

*4060364 (5033405K12RIK)*

*6110204 (OLFR736)*

*6860463 (VGLL4)*

*3610204 (MMAB)*

*2680278 (CIPP)*

*2120301 (CTRL)*

*1980450 (4930485B16RIK)*

*6840551 (HNRPK)*

*5550242 (FCER2A)*

*3520204 (SLC22A8)*

*6860017 (DNMT3L)*

*1660575 (FLOT1)*

*70114 (MAGEB3)*

*6100619 (INPP5E)*

*5290731 (MAPK14)*

*2370039 (OXR1)*

*3840575 (RNASEH1)*

*6100039 (HOXD10)*

*1940082 (OLFR1466)*

*6940142 (PTCRA)*

*450546 (JUND1)*

*4480551 (DDAH2)*

*1230347 (IGK-C)*

*2680128 (1110002H13RIK)*

*3360110 (EDG3)*

*60735 (5830457O10RIK)*

*2450050 (DEFCR-RS10)*

*5130563 (CAV2)*

*3120364 (IKBKAP)*

*6180121 (CUGBP2)*

*7000471 (OLFR836)*

*940398 (TOM1)*

*2260332 (ZCCHC8)*

*6220348 (ACCN5)*

*6900577 (NRAS)*

*460577 (TEX10)*

*2970364 (PRPF4)*

*360576 (NAP1L5)*

*2450167 (NES)*

*6510288 (OLFR1494)*

*3830736 (ZFP184)*

*2940021 (P2RX1)*

*510593 (RIN1)*

*4730193 (H6PD)*

*6370280 (RAG2)*

*450332 (LGTN)*

*4010280 (AI790326)*

*2570114 (LOC330599)*

*1980082 (TRIM36)*

*4230014 (BC052360)*

*4050731 (NOXO1)*

*2510438 (MGAM)*

*2320161 (TTN)*

*2360066 (SPATS2)*

*630053 (P2RY4)*

*2030338 (SGOL2)*

*6400546 (BC037527)*

*3190161 (IL4I1)*

*610368 (EFCBP2)*

*3990195 (ARHGEF12)*

*4760100 (FBXO27)*

*2970021 (SERPINA4-PS1)*

*2650022 (V1RF4)*

*6590324 (N/A)*

*4280040 (GAB2)*

*5720563 (ILTIFB)*

*4810736 (2300009A05RIK)*

*5220427 (POLD1)*

*670435 (4632417N05RIK)*

*2350672 (OAS1F)*

*6420707 (PRRX2)*

*450736 (ALOX15B)*

*5390112 (CDH3)*

*1050170 (HSPA4)*

*6980128 (BC016423)*

*2760010 (STK10)*

*3940577 (A230078I01RIK)*

*6220735 (GCG)*

*1980647 (PRLR)*

*5340632 (2410012C07RIK)*

*2190372 (SALL2)*

*5550725 (MIST1)*

*2970717 (2010109K11RIK)*

*2690438 (UBE3A)*

*6590398 (NFKBIL1)*

*1770091 (CUTL1)*

*2120446 (SLPI)*

*1770358 (LPIN1)*

*4760711 (4930429A22RIK)*

*4200112 (IRX3)*

*6110092 (MYLPF)*

*6130735 (USP12)*

*3520731 (ELL)*

*6350735 (A830023L05)*

*4610133 (BC013672)*

*5080524 (OLFR1106)*

*4280273 (GTSE1)*

*1190273 (AW212394)*

*2850403 (TGIF)*

*4210168 (CTLA2B)*

*730338 (D10BWG0940E)*

*3390671 (C820004B04RIK)*

*4560121 (TAGLN2)*

*5690438 (USP1)*

*2850458 (SH3KBP1)*

*6510315 (1500016O10RIK)*

*4850041 (CDC7)*

*4060537 (1810037B05RIK)*

*130438 (GNRHR)*

*6180373 (WNT2B)*

*3870035 (ZDHHC1)*

*3190348 (SLCO1A1)*

*4200280 (E130113K08RIK)*

*6450524 (IGSF3)*

*6940450 (NKX2-4)*

*3170400 (KCTD2)*

*2970010 (BC048082)*

*5360402 (SRMS)*

*2100082 (TTYH3)*

*1340551 (DPH2L1)*

*7000300 (OLFR166)*

*1410044 (4921508O11RIK)*

*3780019 (KPNB3)*

*6400292 (KLHL5)*

*3170673 (4933425O20RIK)*

*3060180 (ELF2)*

*2350647 (ICAM5)*

*630139 (6030446N20RIK)*

*2260041 (TXN1)*

*6370056 (ABCG2)*

*3190435 (PSIP1)*

*1090551 (CXCL11)*

*4760273 (BC010801)*

*4070301 (BC068281)*

*2690524 (PAP)*

*5910102 (DOCK4)*

*6860204 (ITGBL1)*

*5270341 (4930555F03RIK)*

*6520072 (PADI3)*

*1190551 (LNP)*

*2450458 (IL21R)*

*3830170 (TPRT)*

*2360167 (TRIM50)*

*450551 (1700093K21RIK)*

*4210563 (PNMT)*

*380113 (BMP4)*

*4280129 (RRAD)*

*3290333 (4930563E19RIK)*

*7100494 (OLFR195)*

*2350309 (CAMK1D)*

*1410433 (GRP)*

*3520093 (3110001A13RIK)*

*130079 (ATP6V1G2)*

*6350377 (ACIN1)*

*2970056 (MMP3)*

*6420441 (EDA)*

*3360609 (OLFR918)*

*6760010 (PLSCR4)*

*2260286 (EPB4.1L4A)*

*3800369 (A930010I20RIK)*

*5860215 (OLFR1355)*

*630017 (BPNT1)*

*6420059 (SLC1A1)*

*110348 (CNKSR2)*

*6620592 (TRAM1)*

*630403 (CGA)*

*5290309 (OLFR616)*

*6100551 (2310042P20RIK)*

*4050204 (2810432O22RIK)*

*6980605 (0610008A10RIK)*

*6900026 (FOXN2)*

*2190086 (NFKBIE)*

*5690594 (NDUFB4)*

*4730373 (DCLRE1B)*

*2810128 (P2RY2)*

*4670164 (PDLIM2)*

*2940400 (SIAT7E)*

*7040458 (SEC14L4)*

*3130746 (9930027N05RIK)*

*1570592 (2410080H04RIK)*

*1980592 (SLC12A3)*

*460138 (2810049G06RIK)*

*3440112 (PRR3)*

*2650594 (MS4A10)*

*6200672 (PIRA3)*

*5550435 (1110007A13RIK)*

*360239 (C920006C10RIK)*

*460278 (ATOH1)*

*6130577 (2810457M08RIK)*

*6400133 (GP38)*

*3800537 (MIB1)*

*2030494 (AMACO)*

*6760451 (TLR3)*

*2340138 (H2-M10.1)*

*4060193 (KRT20)*

*7000128 (FTL2)*

*870168 (PDGFRL)*

*6290047 (GPR124)*

*4810593 (PGF)*

*3610369 (IFI203)*

*2650451 (YME1L1)*

*3840128 (CADPS2)*

*4120609 (PSX1)*

*3450164 (N/A)*

*4480110 (CRTAM)*

*130156 (VDR)*

*4540128 (GP6)*

*5900601 (2610528M18RIK)*

*3360010 (RASD2)*

*6760632 (SLC16A13)*

*3440215 (FBLN2)*

*2640369 (SMARCA3)*

*730112 (3110002L15RIK)*

*4480288 (KRT2-8)*

*3060195 (A430107P09RIK)*

*3190253 (OLFR429)*

*6040725 (PLA2G2C)*

*2570300 (PRPF4B)*

*2650348 (6720407G21RIK)*

*6400131 (DEDD)*

*1850528 (ZFP92)*

*630520 (MAPKAPK3)*

*5390687 (C1QA)*

*1850671 (TRF3)*

*1740750 (NRBP)*

*3060494 (TEX2)*

*5570711 (4631426H08RIK)*

*870022 (NIT2)*

*5290735 (KCTD5)*

*5860528 (HEYL)*

*1170400 (PKLR)*

*2260014 (DHX9)*

*2030162 (BC018285)*

*5720075 (TRIK)*

*4010044 (MARE)*

*6760440 (6530413N01RIK)*

*450520 (4930424G05RIK)*

*3130332 (EIF2C3)*

*1090408 (5830446M03RIK)*

*1580438 (B3GAT3)*

*3060605 (PLA2G10)*

*4850273 (4930550B20RIK)*

*4210450 (UBTF)*

*3990408 (KEO4)*

*5080446 (PDE8A)*

*5550017 (1700008P20RIK)*

*2360072 (PPL)*

*2340368 (TM7SF3)*

*3800048 (OLFR1333)*

*2810546 (SLC22A19)*

*7040132 (SYVN1)*

*3140050 (RANBP6)*

*2690168 (SYP)*

*5340138 (5730438N18RIK)*

*1660347 (OLFR975)*

*1580594 (DPYSL3)*

*2900398 (AI481750)*

*510524 (2310010G13RIK)*

*2260731 (SERF1)*

*1690139 (MAT2B)*

*5550162 (CD53)*

*4230463 (PAPPA)*

*5910551 (A730041O15RIK)*

*580286 (RNF4)*

*2360358 (TINF2)*

*5690242 (POU2AF1)*

*4280164 (0710001P18RIK)*

*3440537 (E430036I04RIK)*

*460377 (ELK3)*

*1450441 (0610030H11RIK)*

*840593 (DEF6)*

*1340601 (4933421E11RIK)*

*4230161 (3300001K11RIK)*

*3780075 (GP1BB)*

*2650497 (TACC2)*

*2340176 (GPNMB)*

*3800575 (EIF4G2)*

*2230280 (TRIM33)*

*3190070 (SRCASM)*

*3060035 (SLC18A3)*

*3520164 (MLR1)*

*6900112 (TGFBI)*

*5290484 (PDZX)*

*2850292 (V1RC22)*

*2190170 (ADARB2)*

*3450601 (PRSS11)*

*4560170 (1700122O11RIK)*

*4850082 (GDF10)*

*4560750 (4930429O20RIK)*

*1570348 (HOOK2)*

*6980592 (CDH24)*

*4070037 (GUCY1A3)*

*6220154 (ATP4A)*

*2650673 (2900057K09RIK)*

*3170025 (LAT)*

*2370022 (LOC193676)*

*940242 (MYO9A)*

*6400114 (THOC4)*

*3440687 (C730048E16RIK)*

*3290292 (MCM7)*

*1400014 (SDCBP2)*

*2810279 (4932441K18)*

*6860048 (4933429I20RIK)*

*1340008 (ONECUT2)*

*4920484 (BC033606)*

*6650403 (C230097I24RIK)*

*1660717 (BCAR3)*

*510347 (LOC384415)*

*6110184 (HNRPH1)*

*6590112 (TSSC1)*

*7100300 (1110028E10RIK)*

*3450082 (6530418L21RIK)*

*5860035 (SEC23A)*

*4050600 (NFATC1)*

*3450180 (1200003E16RIK)*

*1410280 (GAB2)*

*4570603 (BC035954)*

*5050537 (ZFP238)*

*460014 (CDH15)*

*6860324 (SNAPC1)*

*6940092 (AI591476)*

*110427 (CAPZA2)*

*5420176 (RAB3IL1)*

*3120440 (MAPK11)*

*3130113 (UCK1)*

*3990450 (0710001C05RIK)*

*4230497 (COPS4)*

*2630577 (PNP)*

*2970463 (HCRT)*

*2690021 (ABAT)*

*4280086 (H2-OA)*

*3870193 (PRKCQ)*

*6380332 (2610301B20RIK)*

*3780364 (BLOC1S3)*

*2940048 (5430439G14RIK)*

*4540053 (A730027B03RIK)*

*5910433 (5330431N24RIK)*

*6130563 (KRTAP16-4)*

*5390746 (4732435N03RIK)*

*450164 (4930418G15RIK)*

*6350632 (ZFP108)*

*2260035 (BC061928)*

*5220162 (4632409L19RIK)*

*1340270 (MKRN3)*

*6370014 (GLCCI1)*

*6110711 (1110001A05RIK)*

*3450121 (2900016D05RIK)*

*6860397 (2310007F12RIK)*

*2690500 (PRP2)*

*1570056 (MCF2L)*

*6040041 (PLEKHA6)*

*3850400 (EIF2S3X)*

*6840403 (UBTF)*

*4850746 (4932411K12)*

*610097 (ACAT3)*

*60575 (JARID1D)*

*2640736 (LOC381783)*

*2570020 (2310046K10RIK)*

*360594 (0610012D14RIK)*

*3140504 (MYO1E)*

*3360064 (DUSP10)*

*4670072 (WFDC1)*

*50142 (B3GNT4)*

*5670021 (D630040G17RIK)*

*5700471 (A730069N07RIK)*

*1190711 (CTSG)*

*5290750 (ACHE)*

*1740484 (LMCD1)*

*2320040 (2300002G24RIK)*

*450064 (4921525D22RIK)*

*110739 (C1QTNF1)*

*4570121 (CHES1)*

*460133 (0610039P13RIK)*

*4560048 (KTN1)*

*510673 (4930532D21RIK)*

*540494 (TK1)*

*4850075 (OLFR980)*

*4050546 (OLFR1471)*

*2120148 (BAD)*

*1190039 (TRPV4)*

*5270446 (CNN2)*

*1340369 (RBP7)*

*4730440 (1600029D21RIK)*

*6380056 (MGC7717)*

*6550605 (6030423D04RIK)*

*6020348 (B930076A02)*

*2450347 (SVIL)*

*4120605 (TRIB2)*

*1570164 (2510040D07RIK)*

*460736 (NEDD4)*

*5670577 (DLGH3)*

*3850671 (1110057H19RIK)*

*5690121 (KIFC5B)*

*4050524 (ATM)*

*3360167 (KLRA16)*

*3060487 (E130115J16RIK)*

*1090075 (ATP2A2)*

*2850280 (GPR151)*

*3610142 (PIGS)*

*840725 (2310057D15RIK)*

*6980722 (1700010M22RIK)*

*2470647 (PUNC)*

*3390064 (PTEN)*

*2350154 (5430404L10RIK)*

*5420091 (GKN1)*

*5390014 (TISP78)*

*670484 (CYCT)*

*2030035 (SMR2)*

*6350025 (KBTBD5)*

*5860170 (PFN4)*

*2760131 (PVRL2)*

*5900091 (MOGAT1)*

*6100537 (SENP1)*

*6130673 (RABEP2)*

*3840458 (FBXL8)*

*1240546 (5930406N14RIK)*

*3450193 (RASSF5)*

*4480546 (SLC35A5)*

*4920070 (MMP12)*

*1240093 (WRN)*

*3390609 (A630038E17RIK)*

*2260142 (GM443)*

*4670563 (KLF13)*

*4920519 (D230007K08RIK)*

*2350056 (SLC12A3)*

*5860446 (BOK)*

*4150014 (WNT8A)*

*4610239 (HES7)*

*6590114 (CXCR3)*

*4070440 (DEFCR-RS12)*

*3450619 (6230425C22RIK)*

*6370010 (2810417D08RIK)*

*1500451 (TNFRSF10B)*

*3800471 (RXRA)*

*1660070 (KRTCAP1)*

*460288 (IGH-VS107)*

*2030164 (CRYAA)*

*3190215 (A230079K17RIK)*

*2100040 (LOC383540)*

*3190068 (V1RC13)*

*4560546 (A)*

*1190292 (USP2)*

*2940435 (BIRC2)*

*4780092 (HIST1H3B)*

*630075 (0610030E20RIK)*

*6380142 (1810033M07RIK)*

*6660593 (KCTD8)*

*6400538 (NTRK1)*

*4050575 (TAS2R104)*

*1340497 (PDCL2)*

*3060242 (ERO1L)*

*5390037 (COPB1)*

*6900168 (ABCC6)*

*6660128 (BC024537)*

*2350014 (SPRRL7)*

*2810095 (HIP2)*

*3940170 (HIST1H3F)*

*3060427 (PKHD1L1)*

*2480647 (6720487G11RIK)*

*4560471 (OLFR1102)*

*1450093 (STRN3)*

*5340332 (LY6H)*

*3190725 (CRIP3)*

*2760066 (CHST7)*

*5700050 (SERPINB6B)*

*430273 (GJA12)*

*6760044 (MRPS18C)*

*3990041 (PHF6)*

*6620088 (IQCF5)*

*6100093 (POR)*

*2760348 (ART3)*

*2850551 (MRPL13)*

*6550575 (PSG23)*

*1500053 (GPRK6)*

*380402 (E330021D16RIK)*

*540193 (2810458H16RIK)*

*380707 (FBXO23)*

*4560095 (BCL7A)*

*6200039 (0610012A05RIK)*

*6770494 (ELMO1)*

*5420114 (DNAJC3)*

*70048 (BC034204)*

*4050193 (ZFP2)*

*1090707 (4432412D15RIK)*

*6660739 (SLC16A2)*

*1980402 (NDFIP1)*

*840338 (DMRT3)*

*3520369 (4921537P18RIK)*

*6860270 (KLF2)*

*3450092 (IKBKG)*

*5900670 (A430093F15RIK)*

*2360672 (4931408L03RIK)*

*2470484 (G430055L02RIK)*

*2760750 (GTPBP2)*

*6290142 (OLFR410)*

*6400022 (2410003P15RIK)*

*2900048 (MIST1)*

*1850722 (TTBK1)*

*6900673 (ARHGEF10)*

*4010619 (PRDX2)*

*4010086 (OPN5)*

*3830253 (HRBL)*

*840373 (CLDN9)*

*6420504 (4930463G05RIK)*

*3850086 (CSE1L)*

*510390 (ADAT1)*

*1850358 (A630014H24RIK)*

*630500 (9930022N03RIK)*

*5860324 (EFEMP1)*

*1400193 (RUNX2)*

*5270040 (5730453I16RIK)*

*6590377 (DEFB9)*

*4200162 (PPFIBP2)*

*730687 (SFRS2IP)*

*6590039 (0610009H04RIK)*

*520398 (TXNDC1)*

*6520114 (D4WSU53E)*

*6510138 (ANPEP)*

*2640725 (5330439J01RIK)*

*2630471 (SSTR1)*

*2370397 (CTS3)*

*5890685 (OLFR44)*

*4730309 (SOX30)*

*1500168 (2810410P22RIK)*

*6420408 (CSF1R)*

*7000136 (MLPH)*

*2370059 (A030008J09)*

*2190050 (UCP2)*

*1190402 (1700020F09RIK)*

*4230647 (4933416E05RIK)*

*70309 (BC011426)*

*2850632 (4632411J06RIK)*

*1980128 (SLC7A5)*

*2060546 (DDX42)*

*2120056 (PPP2R5E)*

*2690102 (OLFR1377)*

*1450270 (4933416I08RIK)*

*60075 (ADAM28)*

*5420035 (AA589507)*

*6400438 (SFRS10)*

*840082 (CSNK1G1)*

*2360164 (RBPSUH)*

*2970372 (DHRS7)*

*510086 (ARHGAP10)*

*1780039 (TNC)*

*6370047 (FGFRL1)*

*4200670 (AW413431)*

*5390047 (1810009A16RIK)*

*5860605 (AI894139)*

*3870592 (MAK3)*

*380035 (ADRA1D)*

*6110717 (E4F1)*

*4070706 (BC033596)*

*2810097 (QPCTL)*

*610092 (C78409)*

*4570577 (MPV17)*

*4200402 (2310001N14RIK)*

*1400619 (STARD10)*

*6220333 (TRPC7)*

*1240053 (CFH)*

*1660022 (MTRF1)*

*6650129 (N/A)*

*1340184 (FGFR1OP2)*

*1850687 (GALNT3)*

*6350093 (CPT1A)*

*3060451 (SURF6)*

*5910577 (AKAP10)*

*3990164 (MGMT)*

*6620673 (RNF17)*

*2340139 (4921511F01RIK)*

*6860603 (CDH3)*

*2230133 (SNAP25)*

*2360438 (1110001K21RIK)*

*2450593 (DACH1)*

*1780333 (NCOA6)*

*5890520 (MMAA)*

*5860373 (2610024N24RIK)*

*540575 (B430203M17RIK)*

*4920156 (4933407H18RIK)*

*2970735 (RAB38)*

*5570128 (PVR)*

*3840711 (RUNX1)*

*2690487 (A630065K24RIK)*

*3870632 (PACSIN1)*

*6590440 (SMPX)*

*2760164 (TRP73)*

*6940086 (2310030G06RIK)*

*6180332 (6330404A07RIK)*

*2680601 (2010008E23RIK)*

*6020711 (SGOL1)*

*6180041 (THUMPD1)*

*5360242 (MTA2)*

*460168 (RUSC2)*

*580181 (GNPDA1)*

*3290022 (ZBTB16)*

*2340465 (F8)*

*7050692 (DMN)*

*460603 (SPG4)*

*5550440 (SERPING1)*

*6130129 (LIMS2)*

*6020671 (M6PRBP1)*

*6040091 (OLFR353)*

*7050280 (FBXW7)*

*6980041 (9130218E19RIK)*

*6980672 (BC022692)*

*1090072 (MILL1)*

*940086 (CPT1B)*

*6180707 (CTTN)*

*6130692 (B930007M17RIK)*

*2100215 (2410016F19RIK)*

*6220047 (ATP5K)*

*70605 (A630091E08RIK)*

*2690333 (SP6)*

*4050736 (RAD51AP1)*

*6980176 (PVRL3)*

*6130546 (6430598A04RIK)*

*2360563 (SNAPC3)*

*3780184 (PDC)*

*1230086 (ZFYVE27)*

*2510594 (2310031L18RIK)*

*6220019 (CCT8)*

*3450471 (CREB3L3)*

*3870129 (RNF138)*

*4010364 (OLFR491)*

*6350717 (2410089E03RIK)*

*6200341 (TIFP39)*

*6660204 (OLFR692)*

*460746 (GYG1)*

*940746 (PKN2)*

*1690180 (NR5A2)*

*1170253 (D930030D11RIK)*

*4010017 (MYH14)*

*3170685 (SLC6A12)*

*4560528 (GTPBP1)*

*2680102 (HIF3A)*

*4920242 (IL28RA)*

*6520487 (MFAP3)*

*4050332 (SYNGR2)*

*4480059 (SGPL1)*

*2760524 (LOC330097)*

*2030541 (CFH)*

*3450008 (ARL11)*

*6900170 (MUT)*

*580433 (5730589L02RIK)*

*5700020 (RYA3)*

*1450575 (1700013N18RIK)*

*5900132 (SHMT1)*

*6590593 (TMEM20)*

*3940403 (OLFR187)*

*6040131 (BTG1)*

*3800411 (MTHFD2)*

*450168 (2600017J23RIK)*

*510021 (1110031B11RIK)*

*6760110 (LIN7B)*

*6660112 (1700016K13RIK)*

*2360463 (NCL)*

*2970161 (RPS6KA2)*

*1770609 (PNPLA5)*

*4590372 (URP)*

*3800114 (VARS2)*

*5690019 (1110018J12RIK)*

*2360465 (THBS3)*

*1410440 (LY64)*

*2810369 (FGF15)*

*3290136 (FOXK1)*

*6620372 (GRIN2D)*

*4010519 (4933402N03RIK)*

*5290605 (RPTN)*

*4150722 (NPPB)*

*1190132 (AW456874)*

*70301 (AY498738)*

*50390 (KLF7)*

*3830239 (KLF4)*

*4230095 (DLGH2)*

*2470348 (2610041P08RIK)*

*3450133 (4930444P10RIK)*

*450739 (POU5F1)*

*5420390 (2410003P15RIK)*

*4010040 (3110070M22RIK)*

*3360181 (AV249152)*

*4060112 (KRT1-12)*

*7000008 (DTX2)*

*1990711 (SLC39A14)*

*2350692 (HDLBP)*

*510082 (LSM2)*

*1850603 (CD47)*

*70292 (OLFR1444)*

*540519 (LOC213233)*

*1780670 (SSX9)*

*3610288 (TCEB3BP1)*

*1450497 (GTL3)*

*2900278 (OLFR1115)*

*510161 (ZFP82)*

*1450195 (1110058A15RIK)*

*6860093 (2810046M22RIK)*

*2120369 (2610205H19RIK)*

*6650167 (SNAPC3)*

*670519 (5930434B04RIK)*

*4540047 (SLC25A18)*

*540528 (UBXD4)*

*1410541 (KLK21)*

*940601 (NCSTN)*

*4590673 (6030452D12RIK)*

*5910408 (GPC4)*

*3290484 (PLA2G12B)*

*3170181 (FBN1)*

*1090377 (OLFR714)*

*5910121 (DDX25)*

*2850095 (D15ERTD785E)*

*840161 (CLCN1)*

*4560400 (NIF3L1)*

*60161 (9930022D16RIK)*

*2230403 (SEMA5A)*

*3610400 (GALE)*

*6350471 (4921509B22RIK)*

*2340008 (E030025L21RIK)*

*2810008 (KLK27)*

*1690632 (SPRN)*

*3170064 (DAB2IP)*

*6770369 (OLFR1023)*

*2340324 (TADA2L)*

*2260735 (TNPO1)*

*6350524 (2610022K04RIK)*

*3830040 (PDLIM2)*

*4010450 (1110003P22RIK)*

*70465 (KBTBD4)*

*5550520 (ITGA5)*

*2650215 (ZFP239)*

*6520546 (PPP2CZ)*

*6290086 (RFNG)*

*870292 (BC002059)*

*6370168 (9130019O22RIK)*

*6350035 (OLFR126)*

*2360017 (GABPA)*

*6200068 (SOLT)*

*1980142 (UBE2H)*

*2680390 (INDO)*

*5270079 (4732452J19RIK)*

*2570092 (HNRPK)*

*5570301 (OLFR459)*

*50100 (APOC2)*

*870070 (OLFR10)*

*3710044 (SF3B3)*

*6350739 (1810057P16RIK)*

*6860020 (EPN2)*

*2060717 (MVD)*

*4230427 (H2-AB1)*

*4480164 (MFI2)*

*4200286 (PAPOLA)*

*1400286 (D5ERTD593E)*

*4480427 (BBS1)*

*5080497 (HAVCR1)*

*7100010 (B830021E24RIK)*

*4670324 (9430076A06RIK)*

*6510433 (FOXP1)*

*4210239 (1110025F24RIK)*

*4210500 (ARCN1)*

*4570082 (1190002H09RIK)*

*6980463 (1700012C15RIK)*

*6650113 (SULT5A1)*

*6130605 (EZH2)*

*6100176 (FCGR1)*

*6020019 (CLSTN1)*

*4230053 (NEDD9)*

*6220176 (TAX1BP3)*

*1340541 (TNR)*

*5570026 (NCOA6IP)*

*2470059 (HOOK3)*

*3060053 (CSDA)*

*6940022 (4930486L24RIK)*

*6040520 (KLHL12)*

*6020427 (4930415O20RIK)*

*3870400 (POLR2K)*

*6220132 (4921528O07RIK)*

*6220341 (GREM2)*

*670341 (SNX17)*

*1500672 (N/A)*

*1850692 (FOXP3)*

*5900176 (ALMS1)*

*2970593 (GALNT4)*

*6650592 (0610037P05RIK)*

*70347 (JAK3)*

*1990494 (RAB25)*

*6180537 (PKHD1)*

*3520435 (USP39)*

*6040594 (4930438A08RIK)*

*5900369 (4930511I11RIK)*

*540524 (2310002A05RIK)*

*4920593 (C730036B14RIK)*

*5130170 (NEGR1)*

*510050 (WNT10B)*

*110025 (KREMEN2)*

*5700039 (CSPG6)*

*5720435 (IGFBP1)*

*4010022 (MYCBPAP)*

*3390524 (1110007F12RIK)*

*460563 (NDG1)*

*2350095 (OLFR1153)*

*430603 (9330164H19RIK)*

*6100128 (MAPK7)*

*3520180 (1600021C16RIK)*

*2900164 (GRIA3)*

*1410288 (MLLT10)*

*2060204 (ZFP128)*

*5080014 (DPEP1)*

*1990070 (D830050J10RIK)*

*7040450 (MARCKS)*

*4120538 (KCTD3)*

*1190458 (SPRR1B)*

*1450059 (DDX50)*

*4010088 (CDK2)*

*1410520 (MYOHD1)*

*3850142 (6030490I01RIK)*

*6420070 (TUBA3)*

*6130746 (PTPN12)*

*1660348 (B930041F14RIK)*

*4200368 (ANP32B)*

*4050725 (9830160G03RIK)*

*70563 (TXNL2)*

*2120692 (5830411K18RIK)*

*4060026 (SNX12)*

*520537 (OLFR1048)*

*5670161 (1700074P13RIK)*

*6940056 (VRK2)*

*5910129 (PRSSL1)*

*1580452 (BCL2L1)*

*5670692 (DEFB15)*

*1980465 (GHSR)*

*2340286 (CDCA8)*

*6450286 (9330175B10RIK)*

*4810541 (CRYZ)*

*2360204 (9430004M15)*

*3940397 (CDCA3)*

*6380563 (NSDHL)*

*840348 (OLFR126)*

*3120148 (SON)*

*3450746 (FV1)*

*610504 (GPRC2A)*

*4070411 (4921511C04RIK)*

*6770053 (ICAM4)*

*430133 (NKX6-2)*

*2970014 (AW456874)*

*6350551 (IMPACT)*

*2650725 (2810439K08RIK)*

*5890500 (PVRL1)*

*3130041 (N/A)*

*5550180 (BC021381)*

*7040408 (2610024G14RIK)*

*870451 (ZFP109)*

*5910292 (C1QB)*

*5080670 (OLFR1079)*

*3060397 (OLFR495)*

*5890717 (VAV2)*

*6370129 (ORF61)*

*840180 (EDARADD)*

*4070497 (SMARCD3)*

*460605 (DACH2)*

*3850047 (DEFB5)*

*3450707 (MBNL2)*

*3440735 (TRAF1)*

*670332 (SCOTIN)*

*7000484 (AW046396)*

*6200671 (AASDHPPT)*

*2260563 (1700030E15RIK)*

*4120053 (ANKRD5)*

*4060746 (SUCNR1)*

*3140441 (SPRRL8)*

*4590121 (DAPK3)*

*60138 (3110001H15RIK)*

*5080019 (SMARCC1)*

*730095 (OLFR114)*

*7050068 (PAK2)*

*870746 (AF233884)*

*1980097 (KLRA3)*

*3130070 (WBSCR22)*

*5340039 (FDFT1)*

*3440129 (RALGPS1)*

*2470736 (5133401N09RIK)*

*5890746 (6530406M24RIK)*

*3990047 (FUT2)*

*6200603 (CRYBB2)*

*4150538 (OLFR74)*

*1940364 (PELI2)*

*2100341 (SLCO6C1)*

*3870164 (FXYD6)*

*6180687 (SLCO4C1)*

*2340193 (1700017I11RIK)*

*2350133 (4930511M11RIK)*

*7040373 (2900045N06RIK)*

*380164 (RORA)*

*3940292 (TCRB)*

*4480537 (STK22A)*

*2680692 (AQP3)*

*6380519 (TEAD4)*

*6020400 (GPR49)*

*1770632 (BIRC5)*

*4010403 (E430026E19RIK)*

*2320136 (5330439J01RIK)*

*730176 (NCALD)*

*6980369 (RNF36)*

*6420338 (2610002I17RIK)*

*7040128 (BC006662)*

*6290398 (PLEC1)*

*510706 (4930527B16RIK)*

*2350403 (CRYGN)*

*4280025 (THAP1)*

*4230059 (1300018K11RIK)*

*5570181 (SPEER4C)*

*1500520 (ARTN)*

*2470079 (CENTG3)*

*3450142 (FZD10)*

*1450717 (MVK)*

*2970390 (1110032D12RIK)*

*1980497 (1700016D06RIK)*

*6980270 (TRPM4)*

*6980112 (CACNG4)*

*3450390 (WDHD1)*

*6840670 (ADAMTS2)*

*2630270 (MRGPRA6)*

*2190451 (ATP2A3)*

*4670519 (VWF)*

*1850093 (GPR73)*

*2940427 (CACNB1)*

*4850102 (ANKRD25)*

*6760687 (MGC65590)*

*3450520 (OLFR1428)*

*6020168 (APBB3)*

*510500 (MRCL)*

*6590095 (CTBS)*

*6590338 (CD5)*

*780670 (2900016G23RIK)*

*5220131 (2900027G03RIK)*

*4570438 (ZFP295)*

*3130044 (BTK)*

*3360053 (TTC18)*

*6290053 (D230039L06RIK)*

*2940632 (THY28)*

*1770121 (ZFP61)*

*1170292 (SUCLA2)*

*4920154 (OLFR555)*

*4070273 (OLFR818)*

*5050154 (SPOCK1)*

*5360040 (ZMYND11)*

*5910731 (MBLDC1)*

*4210594 (STRBP)*

*610148 (1600020H07RIK)*

*2360273 (983000000000)*

*3940139 (PSMD5)*

*4050184 (RAD50)*

*2940162 (MBD3L1)*

*460528 (IARS)*

*4730070 (TAC4)*

*5910093 (TPT1H)*

*4760044 (GM253)*

*1410670 (COL6A1)*

*380093 (SKP2)*

*3190484 (RABGGTA)*

*1580538 (PKD1L3)*

*2690463 (GAPDS)*

*110017 (ETV5)*

*7050056 (CML4)*

*1940685 (UCKL1)*

*2360195 (BC060267)*

*2340707 (SLC22A4)*

*1340373 (B930067F20RIK)*

*5900592 (NLGN2)*

*2970091 (V2R6)*

*450450 (RBBP7)*

*6420176 (SFXN4)*

*4200278 (CUL2)*

*4230722 (1600020H07RIK)*

*6110026 (1810057B09RIK)*

*4150050 (9130012O13RIK)*

*2340092 (CORT)*

*2450333 (USP10)*

*3170021 (2610034E13RIK)*

*6980735 (STK11IP)*

*3990471 (2210409M21RIK)*

*460112 (ACADM)*

*6510059 (MRPS14)*

*4050411 (MOV10L1)*

*4760053 (PCNT2)*

*6550292 (4930563P21RIK)*

*6100358 (C330006K01RIK)*

*3060253 (EBF4)*

*70458 (LIPL2)*

*1500519 (TLE4)*

*1850575 (SLC1A6)*

*1230373 (ZFP114)*

*6900064 (MGA)*

*3170707 (ENAM)*

*3990056 (RPTN)*

*7050504 (MAPRE3)*

*2340148 (2900005J15RIK)*

*2370035 (HDAC5)*

*4210332 (RNF144)*

*4810605 (OLFR1440)*

*4230037 (2310002F18RIK)*

*6980204 (ARL12)*

*3710139 (B230380D07RIK)*

*2480037 (EPHB4)*

*4280184 (ADCYAP1R1)*

*2470292 (CLECSF6)*

*2360148 (AMY1)*

*6110286 (PPM1L)*

*7000605 (DAB1)*

*6550242 (ESRRA)*

*2340097 (CYYR1)*

*6510292 (PBX2)*

*4920575 (MNT)*

*1400687 (TRIM26)*

*460427 (2900011O08RIK)*

*2940204 (MAP2K6)*

*1740398 (IL11)*

*2350273 (AA162070)*

*450280 (4930488P06RIK)*

*3780537 (6720475J19RIK)*

*4540301 (INSIG1)*

*4150253 (0610009C03RIK)*

*450114 (ADPGK)*

*4730176 (BGN)*

*6370148 (OLFR358)*

*60279 (6330548O06RIK)*

*3190551 (4833427G06RIK)*

*3120707 (BC023957)*

*3140333 (BC010462)*

*4610110 (CDH11)*

*5360301 (SLC9A7)*

*4120026 (SURF2)*

*4590072 (SKB1)*

*2570156 (HIST1H2BC)*

*6650402 (FOXM1)*

*3990138 (2410004N11RIK)*

*5570332 (CARD12)*

*130524 (RUFY2)*

*5050687 (HNRPA1)*

*5360601 (GA_X5J8B7W2BV0-3116-4045)*

*770494 (ARL6IP2)*

*4070095 (ADPRTL1)*

*580039 (LRP1)*

*3120039 (PHR1)*

*7040541 (B930095G15RIK)*

*4070309 (SCN3A)*

*1230239 (FOXP2)*

*5910026 (NELF)*

*7050168 (SLC22A18)*

*3130465 (CAR5B)*

*6380450 (ARHGEF6)*

*1450458 (1700051E09RIK)*

*6770484 (A230102I05RIK)*

*4570142 (ATF1)*

*3840133 (PI15)*

*5080139 (MSC)*

*1990672 (4933434I06RIK)*

*1990091 (UNC5D)*

*4120176 (DENR)*

*6100341 (5730405I09RIK)*

*7100739 (SPRR2J)*

*4570195 (DUFD1)*

*1740161 (LOC380799)*

*3780348 (NDUFB9)*

*6370731 (CYP20A1)*

*4780338 (MT4)*

*6840020 (WDR17)*

*50494 (2410141M05RIK)*

*2810093 (D830014E11RIK)*

*770601 (RNPC1)*

*3780114 (ZFP592)*

*5570463 (ACSL4)*

*50671 (CBX8)*

*4200537 (BC002216)*

*6650136 (3010033K07RIK)*

*4730446 (4931406H21RIK)*

*1690176 (C430002N04RIK)*

*2100471 (GRB7)*

*3990458 (SHPRH)*

*2680288 (CD3G)*

*3440324 (KIF17)*

*5860687 (SLC39A11)*

*6100279 (HEBP1)*

*3440021 (DFNA5H)*

*3710035 (G431001I09RIK)*

*5570068 (MCM3)*

*6020280 (MAT2A)*

*5130458 (HCN1)*

*1850433 (2510016D11RIK)*

*130541 (4921507P07RIK)*

*3140242 (SH3BP4)*

*1570403 (OLFR411)*

*6110739 (E130303B06RIK)*

*2230736 (AARS)*

*5690114 (3830408P06RIK)*

*840044 (2010012F05RIK)*

*6760022 (SIX4)*

*6020095 (PDGFA)*

*4590551 (N/A)*

*380270 (MAP3K2)*

*6520687 (N/A)*

*2760133 (1700124P09RIK)*

*1690142 (C1RL)*

*6520100 (RG9MTD2)*

*1340026 (EWSH)*

*4280369 (IL17)*

*6550671 (GNA11)*

*6450369 (LIAS)*

*6040064 (B4GALT3)*

*5390324 (D10WSU93E)*

*5900438 (OLFR389)*

*3990600 (BCKDK)*

*1410450 (4833401D15RIK)*

*1660541 (LPP)*

*2570021 (TM4SF1)*

*3520672 (BC004004)*

*460341 (ACTL7B)*

*2760538 (PRDX6-RS1)*

*1940170 (MATR3)*

*5910605 (1810045K17RIK)*

*3610059 (TUBG2)*

*3850452 (TFF1)*

*6100037 (ONECUT3)*

*3130577 (2310005G13RIK)*

*6760091 (AI173486)*

*3830576 (MYO10)*

*1230279 (CACNA1H)*

*460273 (PLP)*

*4760458 (MID1)*

*4570605 (4933428A15RIK)*

*3940609 (LRRC15)*

*2970577 (KCMF1)*

*1780408 (PSCDBP)*

*1240403 (KHDRBS1)*

*3290044 (FNDC3)*

*6290156 (ABCA1)*

*7050368 (OLFR1184)*

*6130494 (A230046K03RIK)*

*5340156 (MRPL49)*

*4120278 (1110007L15RIK)*

*5270092 (MKNK2)*

*6350671 (HILS1)*

*1690750 (TXNL4B)*

*2570537 (CDC37)*

*3830377 (SLC26A7)*

*6590148 (CBR3)*

*4210288 (MGC25972)*

*2360735 (1700029H01RIK)*

*6760215 (MAPK13)*

*6660121 (ARRB1)*

*1570433 (4833431D13RIK)*

*2940647 (AMOT)*

*1940152 (ASB16)*

*5420465 (PROS1)*

*4210086 (ITM2A)*

*1940398 (SMOX)*

*60619 (C1QTNF4)*

*4610739 (C030038J10RIK)*

*2370162 (RAPSN)*

*2260465 (MS4A7)*

*4730577 (9030409G11RIK)*

*2030075 (OLFR969)*

*4810372 (PACS1)*

*1990358 (LY75)*

*1230019 (BC016076)*

*50110 (SLIT1)*

*6940066 (MPP7)*

*2030594 (KCNA1)*

*2340053 (V1RG8)*

*6940369 (4933416K23)*

*1190195 (ANXA9)*

*6760128 (B230315M08RIK)*

*580471 (BRD4)*

*4610368 (UGT1A6)*

*450128 (CNTF)*

*6770037 (CCL21C)*

*3450162 (ZFP28)*

*4280022 (COPS7B)*

*2900601 (HNRPR)*

*110593 (C330005L02RIK)*

*1980242 (ABCB10)*

*4670377 (GCET2)*

*4850133 (GJB4)*

*510494 (RB1CC1)*

*3940154 (SGTA)*

*4730162 (1700017G21RIK)*

*1770056 (LTB4R1)*

*1660706 (3010027A04RIK)*

*4060152 (B430201A12RIK)*

*2470139 (TMEM16B)*

*4560707 (ZFP291)*

*1190300 (STAG1)*

*5420739 (A930001M12RIK)*

*1850707 (SERPINA6)*

*6550132 (B630009I04RIK)*

*2370711 (SLC35E4)*

*870053 (TRNT1)*

*4610706 (AI987944)*

*2320113 (4930534B04RIK)*

*4070019 (TRIM41)*

*3130324 (N/A)*

*3120025 (LNPEP)*

*3170091 (0910001K20RIK)*

*2900706 (LOC260408)*

*2940450 (LOC381783)*

*1090215 (F12)*

*5890609 (2200002J24RIK)*

*4150706 (OLFR292)*

*3990433 (TRIM33)*

*1570373 (1500001A10RIK)*

*130551 (ADPRHL2)*

*6040161 (PLA2G6)*

*3140368 (CHRD)*

*1340168 (A530054K11RIK)*

*5890403 (DSPP)*

*6180347 (OLFR1209)*

*5130092 (FLT3L)*

*1770097 (EDG4)*

*7100411 (1200013A08RIK)*

*110064 (CCNE1)*

*630121 (CCL17)*

*1090440 (UBE2E1)*

*4920670 (4921505C17RIK)*

*5270577 (EIF4B)*

*2350068 (1700023A16RIK)*

*3850079 (2410018I08RIK)*

*840685 (WNT5A)*

*2810487 (EIF2S2)*

*2850195 (OLFR267)*

*6450095 (2210404O07RIK)*

*5890358 (COMMD8)*

*3440672 (CCR1)*

*770239 (RPS6KA2)*

*450632 (KIF1A)*

*6040102 (PLP)*

*5860577 (AA407526)*

*2900324 (BAG2)*

*6400647 (MASS1)*

*3610398 (6330527O06RIK)*

*4150292 (CAMK2A)*

*6100132 (PTGER1)*

*4210390 (AW552001)*

*3830647 (LRRN6D)*

*1580398 (KISS1)*

*6370632 (GPIHBP1)*

*6550619 (EAF1)*

*2970440 (4932412H11RIK)*

*3780148 (LOC56304)*

*1410068 (2810013E07RIK)*

*840088 (TMOD3)*

*3450056 (KIFC2)*

*2190180 (AB124611)*

*2190114 (OLFR907)*

*4850113 (GALR3)*

*3870093 (ECE1)*

*5910458 (MRPS22)*

*6980164 (FEM1C)*

*4280546 (CNTNAP2)*

*6100347 (PALD)*

*780278 (PRDM2)*

*4780288 (HRMT1L6)*

*6940044 (6230416J20RIK)*

*6590537 (CTLA4)*

*4060551 (BC049702)*

*7040152 (5133400G04RIK)*

*6220040 (TLL2)*

*3170524 (SYNJ2)*

*3710278 (PPP1R2)*

*5900133 (9430015G10RIK)*

*4590047 (9030409E16RIK)*

*3190082 (PLAGL1)*

*3780270 (PPP1CB)*

*2370717 (N/A)*

*4060672 (6030408C04RIK)*

*130411 (MRPS31)*

*1740619 (SOX7)*

*1850390 (ILVBL)*

*2230402 (PTPN13)*

*5890242 (POLR2H)*

*2190368 (GBE1)*

*2340048 (PVRL4)*

*6350722 (PCBP4)*

*4670707 (4921513O20RIK)*

*3850333 (TLR4)*

*6180324 (ZDHHC16)*

*2480463 (SIK2)*

*6100519 (INPP4A)*

*3850270 (ZIK1)*

*4280193 (JAM3)*

*6290403 (CACNA1C)*

*1990021 (IDH1)*

*6380059 (2900026A02RIK)*

*4670706 (PSMD2)*

*4150458 (RNF36)*

*2360427 (SLC24A2)*

*520687 (2810002O09RIK)*

*3990242 (LRRC16)*

*3170341 (HCCS)*

*1770309 (IGL-V1)*

*1410592 (HIST1H1B)*

*5900619 (PCSK2)*

*2630035 (TBX3)*

*5360632 (9030425E11RIK)*

*6040722 (PHKA1)*

*1850403 (DSCAML1)*

*1170280 (BC048937)*

*430519 (OLFR125)*

*3520537 (LOXL1)*

*1990270 (PABPC2)*

*1980114 (C1SB)*

*6770114 (PRSS34)*

*4920008 (ZFP536)*

*3610110 (ATM)*

*6220411 (AGXT2L1)*

*4010465 (CDK5RAP1)*

*4730184 (CCM1)*

*6510110 (MCM6)*

*1980053 (CHGB)*

*6130037 (TNRC6)*

*6590152 (HDGF)*

*4850706 (1210002B07RIK)*

*6200333 (NUMBL)*

*6200435 (2610507B11RIK)*

*3710019 (TLE1)*

*6550273 (TESC)*

*2480136 (OLFR1279)*

*2680593 (A630024B12RIK)*

*2350167 (LSM11)*

*4010722 (SLC22A1)*

*6350132 (SMO)*

*6180164 (PPP2R5C)*

*5270632 (VMD2)*

*2060114 (BC031575)*

*3450021 (4632413C14RIK)*

*1690017 (HSP105)*

*2030021 (BID3)*

*2510575 (FH1)*

*6520397 (CRX)*

*1850047 (IL21)*

*6420341 (LASP1)*

*3850717 (SV2B)*

*5390193 (FLNA)*

*4540121 (OLFR303)*

*4540400 (PACSIN3)*

*2360341 (GPR115)*

*6900154 (GRPEL2)*

*3390053 (RAD51L3)*

*5910040 (1500002O20RIK)*

*2350022 (D15WSU169E)*

*7050044 (1500041B16RIK)*

*3390403 (GPNMB)*

*2680487 (2410015B03RIK)*

*1660053 (RHOT2)*

*6220450 (DOCK5)*

*4070632 (CYP2A5)*

*4610408 (OLFR845)*

*6370484 (TBX5)*

*2030082 (FPR-RS4)*

*6650692 (LMTK2)*

*5910687 (CAPN9)*

*5890452 (MAP3K7IP1)*

*6980142 (THRSP)*

*2350021 (2210008I11RIK)*

*2970152 (C86987)*

*3450593 (CYP2C65)*

*4210601 (MDM1)*

*6130010 (DONSON)*

*2340280 (SMAD9)*

*2690450 (CALCB)*

*3190041 (HADHA)*

*6650008 (ARID3B)*

*5420059 (CHD4)*

*4570338 (COL18A1)*

*4050048 (PRKACA)*

*6130348 (SYNGR2)*

*2630408 (ACAT3)*

*360195 (TIAM2)*

*4120113 (MRPS26)*

*6860333 (CYP20A1)*

*110164 (THRA)*

*1940086 (N/A)*

*6940711 (IL17B)*

*7000333 (STK2)*

*130315 (PTPN2)*

*2190040 (B230113M03RIK)*

*3450465 (1700029F09RIK)*

*6650427 (CYP3A25)*

*3840156 (9130411I17RIK)*

*4010735 (2410129H14RIK)*

*6510156 (CASP14)*

*4920438 (FLT1)*

*6110369 (ACY3)*

*1690377 (A330066M24RIK)*

*4610722 (OLFR788)*

*4210035 (FOXD2)*

*5080091 (UHRF2)*

*3610524 (PPFIA1)*

*1050019 (ALCAM)*

*6040095 (TTC16)*

*6770056 (DEFCR5)*

*4200593 (PVRL3)*

*430242 (1110067D22RIK)*

*1340408 (CYP17A1)*

*6400181 (RBM16)*

*2360092 (A230074B11RIK)*

*1190086 (N/A)*

*4280577 (CLEC2)*

*3440736 (OLFR592)*

*510546 (3200001K10RIK)*

*3120066 (HMGA1)*

*5550138 (RRN3)*

*110053 (KCNC4)*

*1500041 (D4ERTD765E)*

*2850131 (1700027J05RIK)*

*2510154 (D130016K21RIK)*

*3360270 (PLG)*

*360711 (SKP2)*

*4210014 (ASPH)*

*4480593 (LATS2)*

*380092 (CIPP)*

*580600 (C130037N17RIK)*

*2230341 (E130304D01)*

*1240040 (2810403P18RIK)*

*2630070 (OTP)*

*3450435 (NOX1)*

*2190692 (ZFP207)*

*3830048 (5730596K20RIK)*

*4200332 (NETO1)*

*520494 (PSMD10)*

*6770156 (SLC35A2)*

*7100082 (CELSR3)*

*3190746 (GM711)*

*460735 (D2WSU81E)*

*5220010 (ZBTB1)*

*2850500 (MAPKAPK2)*

*2350377 (KRT2-20)*

*6590687 (9230117N10RIK)*

*430500 (ZFP50)*

*6020341 (L3MBTL2)*

*4610484 (D5ERTD708E)*

*5130706 (SULT1A1)*

*4590136 (LY75)*

*2640707 (D330038O06)*

*5890300 (1200015A19RIK)*

*2510605 (ACYP2)*

*4920072 (SLC25A13)*

*3850600 (DGKZ)*

*4150133 (ADPRHL1)*

*520133 (HS6ST2)*

*2350131 (8030462N17RIK)*

*2940138 (PPNR)*

*610215 (MOR253-5)*

*6420050 (1700008B15RIK)*

*6980746 (V1RC12)*

*510008 (WWOX)*

*2060441 (ARRB2)*

*540072 (OLFR1228)*

*2510138 (ZFP36L1)*

*5900292 (4930544G21RIK)*

*1580095 (ANP32A)*

*6840332 (GTPBP4)*

*870279 (9530080O11RIK)*

*6180273 (MSH2)*

*4230154 (OAS1C)*

*2120113 (4933437F05RIK)*

*2940403 (CATSPER2)*

*5290333 (6330503C03RIK)*

*1660136 (2210021A15RIK)*

*460204 (MAGEA9)*

*730097 (MRC1)*

*580204 (CHRNB2)*

*6550452 (IRS3)*

*1780093 (2610009I02RIK)*

*780494 (SVAL1)*

*6420315 (N/A)*

*580292 (N/A)*

*940075 (LUZP1)*

*3610162 (4732418C07RIK)*

*3060471 (SVS1)*

*110603 (ABCB9)*

*3120373 (4930418G15RIK)*

*4050746 (HESX1)*

*4010673 (LOC385253)*

*7100575 (8430437G11RIK)*

*670465 (NOS3)*

*6290152 (GGH)*

*6660364 (RGNEF)*

*4590435 (ZFPN1A4)*

*6130132 (PFN1)*

*2970692 (PZP)*

*1050435 (BC003324)*

*4570010 (CACNB3)*

*5860008 (TMEM16F)*

*3710136 (SUMO3)*

*4920746 (OLFR131)*

*4610121 (FBXO24)*

*1240022 (SCN1B)*

*2370139 (KIF1B)*

*1240019 (MAGEB2)*

*6940040 (ASTN2)*

*1980398 (D14ABB1E)*

*6110577 (1700108E19RIK)*

*4570397 (CORO1C)*

*3190750 (SLFN4)*

*3710746 (TAF12)*

*2450088 (ASB7)*

*2350687 (CREB3L1)*

*3140019 (GDAP1)*

*5340280 (FBP1)*

*4760338 (CPEB2)*

*5910086 (EHMT1)*

*7000576 (GAST)*

*1780494 (1110007F05RIK)*

*870458 (B930090D16RIK)*

*6620324 (OLFR395)*

*1940053 (1110020G09RIK)*

*3990372 (ARHGEF16)*

*6840301 (ADH4)*

*3940458 (COPS7A)*

*6660301 (PBX1)*

*2760671 (RYR2)*

*4920390 (9930116P15RIK)*

*5860593 (BC052066)*

*2190079 (OLFR508)*

*1980390 (0610012K18RIK)*

*4810239 (ISL1)*

*3440692 (SCFD1)*

*4200064 (FBXL20)*

*580725 (AI427122)*

*5910438 (1700123I01RIK)*

*7100068 (GNA11)*

*4540039 (HIST1H2AI)*

*3390632 (SEC23B)*

*6660180 (ACN9)*

*2760161 (8430417A20RIK)*

*5570672 (N/A)*

*2690750 (PCX)*

*6550020 (G0S2)*

*4480050 (VDRIP)*

*2680397 (IDH2)*

*7000397 (OLFR1038)*

*6620408 (SDK2)*

*6450400 (N/A)*

*1980195 (V1RG11)*

*5670398 (GARNL1)*

*2340463 (1700019P01RIK)*

*3870133 (SSFA2)*

*1980711 (A330008L17RIK)*

*6550632 (PANX1)*

*6840369 (3110050N22RIK)*

*2760408 (SFRS7)*

*2100315 (KRTAP3-2)*

*2690600 (CYP26A1)*

*2260164 (ABCC9)*

*2060494 (HSH2D)*

*2690253 (PABPC1)*

*5420021 (ITPKC)*

*3830358 (6030465E24RIK)*

*5720270 (PGA5)*

*6040010 (SHMT2)*

*3870731 (TNFRSF19)*

*1450047 (UQCRB)*

*7050465 (NR2E3)*

*1190050 (1700023F06RIK)*

*7000152 (1110051M20RIK)*

*2470066 (APG10L)*

*3190162 (SMYD1)*

*6370368 (CCK)*

*1190048 (ICOS)*

*1850039 (UTP14B)*

*6110086 (N/A)*

*4120102 (1210002B07RIK)*

*1980020 (TNC)*

*870131 (2310046K01RIK)*

*70253 (EMILIN2)*

*3360711 (KBTBD3)*

*4280333 (D830007F02RIK)*

*4200603 (PIWIL1)*

*5290433 (EHMT1)*

*7100270 (HIST1H3I)*

*1090358 (STYX)*

*2650021 (ZCCHC5)*

*6900253 (YWHAE)*

*5700100 (LMAN1L)*

*1400593 (V1RE2)*

*2340538 (PSMD11)*

*6380402 (ST14)*

*4760452 (SGK3)*

*1690504 (ZFP212)*

*1410746 (CACNG5)*

*510195 (CLCN3)*

*3710692 (OLFR645)*

*1190332 (CTH)*

*4480088 (0710001B24RIK)*

*610563 (SLC2A10)*

*5910692 (RNF44)*

*6900025 (ARIH1)*

*2100170 (2810021G02RIK)*

*4050537 (STARD5)*

*4610538 (GMPPB)*

*3610446 (ACVR1B)*

*3120161 (P2RY12)*

*4560066 (A830008O07)*

*3140450 (3110057O12RIK)*

*6370273 (1110014F12RIK)*

*6420039 (TULP2)*

*6590020 (4833412N02RIK)*

*4070685 (CD209B)*

*4120035 (9930021J03RIK)*

*3800452 (RFC5)*

*1780603 (SERPINA3C)*

*3840601 (RNF38)*

*4670338 (SPG4)*

*4670204 (V1RC3)*

*6380082 (RTKN)*

*4480577 (TRPC6)*

*3990288 (A830094I09RIK)*

*2360519 (SLC25A4)*

*3710167 (OLFR1324)*

*5550687 (2610315E15RIK)*

*1090390 (TNFRSF1A)*

*2100301 (TBX22)*

*2060279 (PPP2R1A)*

*6420332 (E030024M05RIK)*

*3840121 (GPHN)*

*510324 (CHKA)*

*6110059 (SERPIND1)*

*4050010 (LY6D)*

*110288 (EFS)*

*3610091 (SSTY1)*

*4480301 (LOC380797)*

*2900338 (9530090G24RIK)*

*3940288 (RAB3A)*

*6450364 (HIF3A)*

*3290707 (MAP3K9)*

*7000594 (GART)*

*1740670 (DNAJC12)*

*3940309 (BC024806)*

*60025 (IGH-VJ558)*

*4230070 (5730411O18RIK)*

*5270711 (TAS2R126)*

*4670170 (MYC)*

*2970008 (ASPH)*

*3520008 (FBXL12)*

*5700338 (RAB40B)*

*1660181 (9330155M09RIK)*

*3520603 (NCDN)*

*5220088 (ZFP365)*

*6200014 (ITM2B)*

*2760575 (ASB5)*

*5290253 (TMC7)*

*2630451 (4930546H06RIK)*

*4150129 (4930402E16RIK)*

*6450672 (KCNS2)*

*6100731 (2210023C10RIK)*

*7100711 (CDH20)*

*510180 (ZFP36L2)*

*1850609 (ELMO1)*

*6860563 (SVS2)*

*4540086 (OLFR1141)*

*6940093 (XLR5)*

*3390450 (CACNA1F)*

*2480324 (NR3C2)*

*3190600 (A330045H12RIK)*

*630112 (SUV39H1)*

*3120504 (TCP10A)*

*1770025 (DGKG)*

*2320195 (PEX11A)*

*2510040 (OLFR982)*

*6980600 (SNX7)*

*3940463 (OLFR716)*

*5890020 (RAB10)*

*4010020 (D630045E04RIK)*

*6760239 (AI450757)*

*2650687 (KCNF1)*

*1170070 (AMPD2)*

*6400433 (MDM1)*

*450047 (BC028440)*

*4050180 (1500032D16RIK)*

*5340672 (BC065120)*

*1850594 (9930023K05RIK)*

*130044 (ROCK1)*

*4610546 (IGHMBP2)*

*1980601 (ENTPD7)*

*5290280 (9130017C17RIK)*

*5130113 (IGF2BP1)*

*2450056 (ATP12A)*

*2940725 (SPEER1-PS1)*

*1850647 (FOLR4)*

*2340592 (MCRS1)*

*1990280 (1700029I01RIK)*

*6620300 (STK19)*

*4560022 (HNMT)*

*3870286 (OGN)*

*4280435 (C130090K23RIK)*

*5690465 (FGD3)*

*5290148 (PTPRC)*

*4150139 (UBE1X)*

*70068 (HSD3B2)*

*2510577 (BTBD3)*

*4810113 (MXD4)*

*430390 (V1RD12)*

*1240358 (MAD2L2)*

*6200364 (C130099A20RIK)*

*3450739 (ZFYVE16)*

*2450193 (SLC7A1)*

*580484 (ORF61)*

*4610348 (CALU)*

*2030632 (1300018P11RIK)*

*2480082 (2610528H13RIK)*

*7050286 (SPAG16)*

*5080463 (ETV1)*

*4210131 (GNAQ)*

*1090725 (ZFP532)*

*3610458 (DBH)*

*2260722 (SACY)*

*2340021 (ACTC1)*

*2680014 (HECTD2)*

*5860139 (FEV)*

*6220070 (2810408B13RIK)*

*5360440 (IL7)*

*50040 (NCDN)*

*4230148 (TAPBP)*

*2850128 (OLFR196)*

*3140110 (A330019N05RIK)*

*5220239 (FGA)*

*5270458 (NPC1L1)*

*6980079 (ACBD4)*

*6650075 (PTPRCAP)*

*2810113 (2310035C23RIK)*

*4780022 (ANXA8)*

*2970167 (BC052360)*

*6520239 (EXT2)*

*6380300 (PPP1CC)*

*4540168 (SPAG4L)*

*3060736 (N/A)*

*510441 (SCARB1)*

*7040037 (9130005N14RIK)*

*6760372 (9230112E08RIK)*

*940152 (HOXA4)*

*3390440 (1110013G13RIK)*

*7050082 (GJC1)*

*5700672 (BC020025)*

*2120403 (ADA)*

*1660735 (RNF146)*

*6900403 (DNMBP)*

*3780035 (BC027246)*

*2680215 (4930524B15RIK)*

*1740687 (HMOX1)*

*2470735 (NFATC4)*

*2680524 (TEBP)*

*4070097 (5730427C23RIK)*

*2850575 (N/A)*

*5890148 (1110001A05RIK)*

*4730279 (TNK1)*

*2810451 (NBR1)*

*1770154 (C6)*

*6350563 (2510005D08RIK)*

*6940019 (SLC25A27)*

*5900019 (9030605E16RIK)*

*5290600 (0610012H03RIK)*

*460725 (SEPTIN 5)*

*2510148 (HGFAC)*

*6400372 (E130016E03RIK)*

*6650397 (YPEL3)*

*6130450 (GABPB2)*

*3450301 (BCL7B)*

*2230242 (SLC34A3)*

*1410088 (4632418H02RIK)*

*7000706 (LARS2)*

*4280008 (1110020M19RIK)*

*2940524 (2310003P10RIK)*

*450746 (B230354K17RIK)*

*3290600 (ACVRL1)*

*2570433 (PCDHGB6)*

*6450594 (DLC1)*

*2680504 (DHRS8)*

*2370110 (CYP4A14)*

*450129 (PLCZ1)*
